# HIV Prevention Via Mobile Messaging for Men Who Have Sex With Men (M-Cubed): Protocol for a Randomized Controlled Trial

**DOI:** 10.2196/16439

**Published:** 2019-11-15

**Authors:** Patrick Sean Sullivan, Ryan J Zahn, Sarah Wiatrek, Cristian J Chandler, Sabina Hirshfield, Rob Stephenson, Jose A Bauermeister, Mary Ann Chiasson, Martin J Downing Jr, Deborah J Gelaude, Aaron J Siegler, Keith Horvath, Erin Rogers, Ana Alas, Evelyn J Olansky, Heather Saul, Eli S Rosenberg, Gordon Mansergh

**Affiliations:** 1 Department of Epidemiology Rollins School of Public Health Emory University Atlanta, GA United States; 2 Department of Medicine SUNY Downstate Health Sciences University Brooklyn, NY United States; 3 Center for Sexuality and Health Disparities School of Nursing University of Michigan Ann Arbor, MI United States; 4 Mailman School of Public Health Columbia University New York, NY United States; 5 Division of Infectious Diseases Columbia University Medical School New York, NY United States; 6 Lehman College City University of New York Bronx, NY United States; 7 Division of HIV/AIDS Prevention Centers for Disease Control and Prevention Atlanta, GA United States; 8 Department of Behavioral Sciences and Health Education Rollins School of Public Health Emory University Atlanta, GA United States; 9 Department of Psychology San Diego State University San Diego, CA United States; 10 Health, Science and Human Services Division ICF Atlanta, GA United States; 11 Department of Epidemiology and Biostatistics University at Albany - SUNY Albany, NY United States

**Keywords:** men who have sex with men, HIV prevention, HIV care

## Abstract

**Background:**

Men who have sex with men (MSM) continue to be the predominately impacted risk group in the United States HIV epidemic and are a priority group for risk reduction in national strategic goals for HIV prevention. Modeling studies have demonstrated that a comprehensive package of status-tailored HIV prevention and care interventions have the potential to substantially reduce new infections among MSM. However, uptake of basic prevention services, including HIV testing, sexually transmitted infection (STI) testing, condom distribution, condom-compatible lubricant distribution, and preexposure prophylaxis (PrEP), is suboptimal. Further, stronger public health strategies are needed to promote engagement in HIV care and viral load suppression among MSM living with HIV. Mobile health (mHealth) tools can help inform and encourage MSM regarding HIV prevention, care, and treatment, especially among men who lack access to conventional medical services. This protocol details the design and procedures of a randomized controlled trial (RCT) of a novel mHealth intervention that comprises a comprehensive HIV prevention app and brief, tailored text- and video-based messages that are systematically presented to participants based on the participants’ HIV status and level of HIV acquisition risk.

**Objective:**

The objective of the RCT was to test the efficacy of the Mobile Messaging for Men (M-Cubed, or M3) app among at least 1200 MSM in Atlanta, Detroit, and New York. The goal was to determine its ability to increase HIV testing (HIV-negative men), STI testing (all men), condom use for anal sex (all men), evaluation for PrEP eligibility, uptake of PrEP (higher risk HIV-negative men), engagement in HIV care (men living with HIV), and uptake of and adherence to antiretroviral medications (men living with HIV). A unique benefit of this approach is the HIV serostatus-inclusiveness of the intervention, which includes both HIV-negative and HIV-positive MSM.

**Methods:**

MSM were recruited through online and venue-based approaches in Atlanta, Detroit, and New York City. Men who were eligible and consented were randomized to the intervention (immediate access to the M3 app for a period of three months) or to the waitlist-control (delayed access) group. Outcomes were evaluated immediately postintervention or control period, and again three and six months after the intervention period. Main outcomes will be reported as period prevalence ratios or hazards, depending on the outcome. Where appropriate, serostatus/risk-specific outcomes will be evaluated in relevant subgroups. Men randomized to the control condition were offered the opportunity to use (and evaluate) the M3 app for a three-month period after the final RCT outcome assessment.

**Results:**

M3 enrollment began in January 2018 and concluded in November 2018. A total of 1229 MSM were enrolled. Data collection was completed in September 2019.

**Conclusions:**

This RCT of the M3 mobile app seeks to determine the effects of an HIV serostatus–inclusive intervention on the use of multiple HIV prevention and care-related outcomes among MSM. A strength of the design is that it incorporates a large sample and broad range of MSM with differing prevention needs in three cities with high prevalence of HIV among MSM.

**Trial Registration:**

ClinicalTrials.gov NCT03666247; https://clinicaltrials.gov/ct2/show/NCT03666247

**International Registered Report Identifier (IRRID):**

DERR1-10.2196/16439

## Introduction

### Background

Men who have sex with men (MSM) face the highest burden of HIV in the United States [1], and there is a paucity of efficacious or promising mobile health (mHealth), HIV-prevention interventions tailored specifically for MSM [2]. New HIV prevention tools are needed that can address the needs of MSM, especially young MSM aged 15-24 for whom yearly HIV incidence doubled from 2002 to 2014 [3], and for young MSM of color for whom these burdens are most severe [4-6]. Currently, HIV prevention services are underutilized by MSM, with just over half (56%) reporting being tested in the past 12 months, high proportions (76% of HIV-positive and 66% of HIV-negative MSM) reporting recent condomless, receptive anal intercourse, and few (<20%) reporting utilization of pre-exposure prophylaxis (PrEP) [7]. Statistical models of MSM epidemics parameterized to represent US epidemics demonstrate that high levels of prevention service coverage will be required to substantially reduce HIV incidence [8,9], and increased utilization of routine prevention activities, like frequent HIV testing, may enhance the uptake of biomedical interventions like PrEP [10]. At the same time, engaging MSM living with HIV in mHealth HIV-prevention efforts is critical and addresses the two most important factors that determine cost-effectiveness, namely the HIV prevalence of the target population and the cost per person reached [11].

Researchers in the past decade have been exploring the most effective ways to engage MSM in a package of HIV prevention services. A growing body of research has suggested that mobile phone apps provide a dynamic environment for intervention and can offer on-demand prevention services for MSM [12-17]. This research indicates that MSM are open to receiving prevention information and resources via apps [18]. Electronic messages communicated through apps, known as mobile messaging, are an appealing approach to enhance intervention uptake because they allow for messaging that can reach a wide audience of MSM, including rural MSM [16]. Further, younger MSM might be especially interested in using mobile technology to receive health information [19]. This study builds on an existing HIV prevention app designed for MSM, HealthMindr, to add and evaluate tailored prevention messaging.

### The HealthMindr App

The HealthMindr app is a comprehensive HIV prevention app for MSM [20]. Developed using social cognitive theory [20,21], HealthMindr features basic prevention services, including: screening for HIV and sexually transmitted infection (STI) risk; a scheduling and reminder system for routine HIV testing; a PrEP eligibility screener; a nonoccupational postexposure prophylaxis (nPEP) risk assessment tool; an ordering platform for delivery of at-home HIV and STI screening kits, condoms, and lubricant; and service locators for HIV and STI testing, and PrEP, nPEP, and HIV treatment and care. The app was built based on extensive input from MSM, public health leaders, and staff from community-based organizations [22,23]. The basic app (before the addition of messages and videos) was pilot tested with 121 MSM in Atlanta and Seattle, and the results indicated high levels of acceptability, use of tools to develop and maintain a consistent HIV testing routine, use of PrEP screening and referrals, and ordering of at-home HIV test kits and condoms [20].

### Study Intervention: HealthMindr Plus Brief Messages

The current study utilized the HealthMindr platform with the addition of social cognitive theory–based, sexual health messaging components. For this randomized controlled trial (RCT), we developed a series of brief text-based (n=63) and sourced a series of video (n=12) messages designed designed to promote health-seeking behavior and further the adoption of sexual health services recommended by HealthMindr. The messages were delivered through the HealthMindr platform and aligned with the prevention tools offered in the app [20]. The purpose of this study was to evaluate the use and efficacy of the mobile-messaging platform as a public health strategy for improving sexual health outcome measures among MSM by determining whether exposure to the message-delivery platform resulted in improvements in participants’ self-reported sexual health and prevention behaviors, beliefs, and attitudes. Specific aims to accomplish this purpose included two phases: (1) formative research (focus groups and in-depth interviews) for app message development; and (2) an RCT for testing efficacy of the app. This paper describes the methods for this study in more detail.

## Methods

### Overview

**Figure 1:**
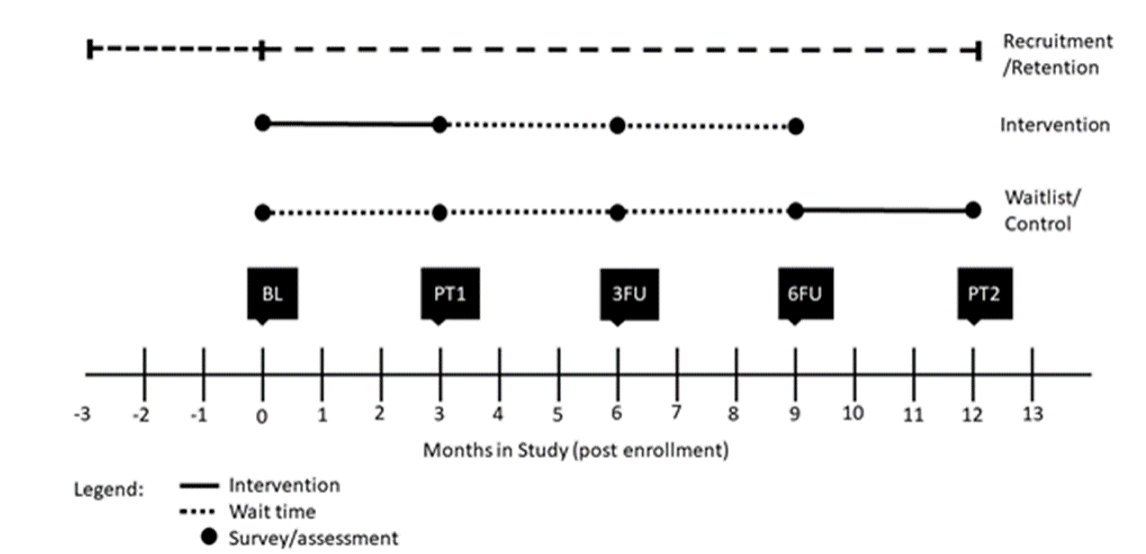
Timeline of participant activities and assessments. Only participants from the waitlist/control group who opt to participate in the intervention will be assessed in PT2. BL: baseline survey; PT: postintervention assessment; FU: follow-up survey.

The Mobile Messaging for Men (M3) study was conducted in two phases. The first phase consisted of a series of focus groups and in-depth interviews to ensure that written messages, videos, and app features presented in the trial were appropriate for the audience. The second phase was an RCT, with MSM in three serostatus/risk groups randomized to receive either the intervention condition (M3 app) for a three-month exposure period or a waitlist-control condition with the option to receive the app for three months at the end of the study. The serostatus/risk groups were defined by self-report as: (1) HIV seropositive; (2) HIV seronegative at higher risk (condomless anal sex and not taking PrEP as prescribed in the past 3 months); and (3) HIV seronegative at lower risk (no condomless anal sex in the past 3 months, or condomless anal sex while taking PrEP as prescribed in the past 3 months). Postintervention data collection occurred at three time points: 3 months (immediately postintervention), 6 months (three-months postintervention), and 9 months (six-months postintervention) (see Figure 1). Participant follow-up will be completed in September 2019, with primary outcome analyses to follow. The study was reviewed and approved by the Emory University Institutional Review Board (Protocol #87684) and registered at ClinicalTrials.gov (NCT03666247).

### Formative Research for Online Messages

#### Message Development Process

Message development for qualitative input from the target audience took two forms: brief written message and video message development. Two committees of subject matter experts (written messages: JB, GM, PS, KH, MD, EO, RZ; video messages: SH, MAC, EO, DG, BB, RZ, RS) were established to implement written and video message creation. First, the committees determined six domains of prevention messages and related study outcomes: (1) condom use; (2) HIV testing; (3) STI testing; (4) PrEP continuum outcomes; (5) antiretroviral therapy (ART) use; and (6) engagement in care.

The written message committee reviewed the literature on HIV prevention messages in the six domain areas, as well as messages used in their ongoing research, and developed 63 messages that were 2-3 sentences each and were organized into the 6 prevention domains. Messages within each domain were mapped onto Social Cognitive Theory constructs (eg, information, relevance, norms, barriers, and self-efficacy). Through the mobile app, each participant received a common core of 36 messages (approximately 6 messages per domain) and another set of 9 secondary messages specific to each of the 3 serostatus/risk groups. For HIV-positive men, these secondary messages focused on ART uptake and adherence, for HIV-negative men who reported condomless anal sex in the past three months, additional messages pertained to PrEP, and for HIV-negative men who did not report recent condomless anal sex, additional messages were about condom use and HIV/STI testing. Participants were sent a written message every 1-2 days and a video message once per week.

The video messages were selected after identifying existing videos in the field of recent HIV and STI prevention related to the six domain areas, with the goal of identifying 12, approximately one-minute video clips for delivery on the mobile app. About two videos per domain area were delivered to all study participants at a rate of one per week. Through online searches and common, prevention video–development funding agencies (eg, the Centers for Disease Control and Prevention and the Kaiser Family Foundation), an initial pool of several hundred videos was identified, and they were systematically and iteratively reduced in number by the subject matter expert committee through several rounds of reviews and ratings.

### Phase 1: Formative Research

#### Qualitative Message Development Process

Two qualitative methods were used to elicit feedback from MSM who were potential participants in the RCT: focus group discussions and in-depth interviews. The goals of the qualitative phase were to: (1) develop knowledge around possible topics for HIV prevention messaging, preferred modes of receiving mobile prevention messages, and messaging frequency; and (2) develop HIV status/risk level-specific prevention messages intended for dissemination to MSM, cognitively test the messages, and finalize messages for inclusion in the RCT.

Nine focus group discussions, one per risk group per study city, were conducted. Focus group discussions focused on message delivery aspects, including mode, format, length, framing, delivery source, and other non-content related characteristics. Participants were prompted to describe notable HIV prevention messages they had encountered prior to their engagement with this study, describing both the content and format of the message as well as their thoughts on its efficacy and applicability. Participants were then asked to complete a pile-sorting activity [24], where participants were shown cards with HIV prevention messages and were asked to sort them into piles representing how they would like those messages to be received (eg, a card contained a message about regular condom use, and choices for delivery of message included video, scientific facts, or fun messaging). The aim was to understand which delivery and content factors were important for the delivery of each type of prevention message.

Two rounds of in-depth interviews were conducted: one round for text-based messages and one round for video messages. These in-depth interviews assessed the extent to which the predeveloped text- or video-based messages were understood and believed, and how messages needed to be customized to address contextual differences and variations in prevention needs, such as local context, demographic contexts, risk group, or relationship contexts. For the written messages, 18 in-depth interviews were conducted, two per risk group per study city. Participants completed a ranking activity in which they were given cards on which predeveloped written messages were printed. Within each of the six domains listed above, participants ranked the messages from most effective to least effective. They were then asked about their reactions to each message, including their comprehension, willingness to read the message, appropriateness of the message and word choice, and their perception of their ability to enact behavioral change. For the video-based messages, 26 in-depth interviews were completed across the study sites. Participants were shown each video-based message and, after each, were asked about their reactions to the videos, including identification of intended messages, comprehension of the messages contained in each video, and willingness to view the message. Participants were also asked about specific elements of the video, such as length, style, language use, and how each video might be made more effective. Based on the feedback from participants in these focus group discussions and in-depth interviews, each written and video-based message was revised or edited by the subject matter experts to be made more suitable for inclusion in the RCT.

#### Intervention Messaging

The M3 intervention provided risk-customized written and video messages for participants, augmenting the core prevention services available in HealthMindr. Core messages were delivered to all participants regardless of HIV status and risk group. Secondary messages were delivered to participants based on their self-reported HIV status and risk group assignment at baseline; risk group status was dynamic during the trial and updated using data from monthly check-ins during the app exposure period or by participant self-initiation of check-in. Message domains for core and secondary messages are summarized in Table 1. Video messages were 60-80 second excerpts from existing videos related to HIV prevention and education. A total of 12 video messages were offered as part of the intervention for all participants regardless of risk group.

**Table 1:** M3 domains in the mobile app.

Domain	Core messages	Secondary messages		
	All risk groups	Lower risk HIV-negative	Higher risk HIV-negative	HIV-positive
Condoms	✓	✓	✓	
STI^a^ testing	✓	✓	✓	✓
HIV testing	✓	✓	✓	
PrEP^b^	✓		✓	
ART^c^	✓			✓
Engagement in care	✓			✓

^a^STI: sexually transmitted infection.

^b^PrEP: pre-exposure prophylaxis.

^c^ART: antiretroviral therapy.

**Figure 2:**
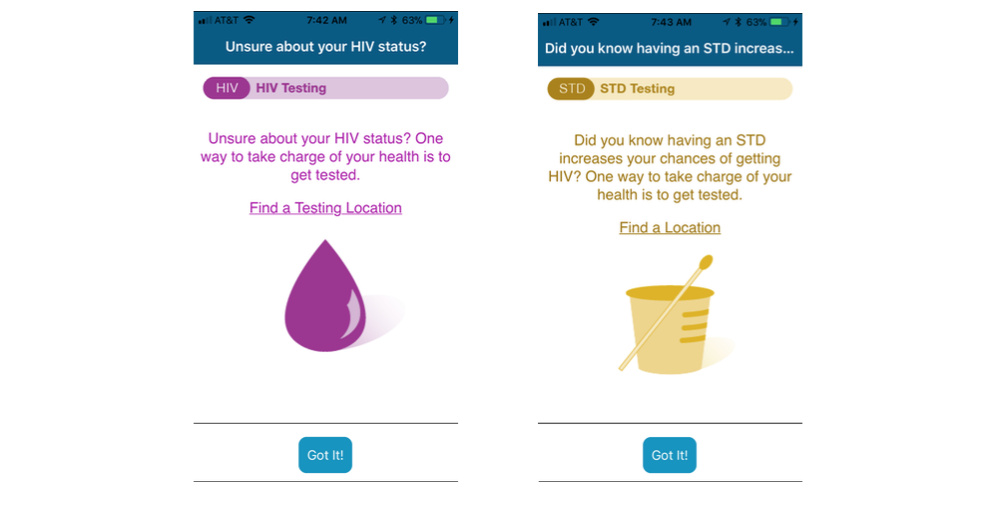
Example text-based messages from the M3 app.

Messages were provided to participants according to a predetermined schedule and in a consistent sequence that started on their date of enrollment. Most messages provided a hyperlink to relevant services referenced in the message. For example, a message to promote routine HIV screening was paired with a link to the app function for scheduling routine HIV screening (Figure 2).

#### Commodity Ordering

Participants assigned to the intervention group also had the ability to order prevention commodities (HIV at-home self-test kits, home specimen collection kits for STI testing, condom variety packages, and condom-compatible lubricant) directly through the app for delivery to an address of their choosing. The kits were purchased or built by Emory University, and delivered through Amazon Fulfillment Services. At-home HIV self-test kits were fulfilled with OraQuick kits (Orasure Technologies, Bethlehem, Pennsylvania, United States). At-home specimen collection kits (CareKits) were provided through the Emory Center for AIDS Research, and included instructions and materials for participants to collect urine, a rectal swab, a pharyngeal swab, and microtainer blood specimens sufficient to support testing for urethral, rectal, and pharyngeal gonorrhea and chlamydia, and for syphilis. Specimens were returned to the study laboratory by mail, and STI testing was performed in a Clinical Laboratory Improvement Amendments–certified laboratory using methods previously reported [25]. Negative STI results were provided to participants through emails or text messages; positive STI results were provided to participants by phone call with referrals to treatment. Positive STI test results were reported to state and local health departments, as required by law and as disclosed to participants during the informed consent process; HIV self-tests are sent by participants directly to the vendor and thus HIV self-test results during the study were unknown to staff.

### Phase 2: Randomized Controlled Trial

A total of 1229 MSM were recruited and enrolled into a randomized controlled trial in three US cities with substantial HIV incidence: Detroit, Michigan; New York City, New York; and Atlanta, Georgia [26]. The intervention of HIV prevention messages and HealthMindr services were delivered as a mobile phone app, available on both iOS and Android operating systems. At the time of randomization, all participants received standard of care referrals to HIV prevention and treatment services in the form of a paper list of local prevention resources.

### Study Design and Procedures

#### Overview

Among the 1229 men enrolled, 478 were from Atlanta, 335 from Detroit, and 416 from New York City. Men were randomly assigned to either the immediate intervention group or the waitlist-control group at a 1:1 ratio. All participants received the same surveys at baseline and at three-month intervals thereafter. At baseline, participants assigned to the intervention group installed the M3 app and study staff gave them an orientation on its use. These participants received access to the full mobile app and messages based on their risk profiles over a period of three months, after which the app was deactivated. Wait-list control participants were not provided access to the intervention app at the baseline visit but continued to receive quarterly surveys. At nine months postenrollment, participants in the waitlist-control group were given the option of accessing the intervention app. Those who opted to do so were given the app for three months and were asked to complete one final study visit and survey at the 12-month point. Figure 2 provides an overview of the study timeline and includes study visits, procedures, and the participants involved at each time point.

#### Recruitment

Recruitment activities were conducted over the course of 10 months in each study city from January-October 2018. To reflect the diversity of MSM, we used a multi-modal recruitment strategy with a goal of recruiting a sample of MSM who were diverse in terms of age, race/ethnicity, and HIV risk. Modes of recruitment included targeted banner advertisements (eg, Facebook), traditional print advertisements (eg, flyers, public transit), recruitment at venues, referrals from community service providers, and in-person outreach.

#### Eligibility

Men eligible to participate in this study were: (1) aged 18 years and older; (2) assigned a male sex at birth; (3) self-reported their current gender identity as male; (4) self-reported anal intercourse with a man in the past year; (5) were current residents of the study city metropolitan area (Atlanta, Detroit, or New York City); (6) planned to stay in the city area for the next nine months; (7) owned and used an Android or iOS smartphone; (8) were able to read and understand English without assistance; and (9) were included in one of the three groups of serostatus and risk groups described above. As of November 2018, all study participants provided written informed consent prior to participating in the study.

#### Enrollment

Men recruited in community venues were offered a brief interviewer-administered screening survey; men recruited through flyers or online venues were offered a brief online eligibility screener. Eligible men were invited to attend an in-person baseline enrollment visit. At the enrollment visit, research staff reviewed consent documentation with potential participants and reconfirmed eligibility criteria. Following consent and enrollment, all participants completed a baseline behavioral survey (see Multimedia Appendix 1) that collected information related to primary and secondary outcomes, including: (1) demographic characteristics; (2) HIV and STI status and testing history; (3) condom use; (4) PrEP use and adherence (for HIV-negative participants); (5) ART use and adherence (for HIV-positive participants); (6) knowledge, perceptions, beliefs, intents, and communication with sex partners about HIV status and risk reduction; (7) mobile phone and data usage; (8) access to Internet and information; and (9) psychosocial covariates. The initial visit took up to 90 minutes.

#### Randomization

Following completion of the behavioral survey, participants were randomized into either the intervention or the wait-list control group. Participants were successfully randomized within 18 strata based on the three serostatus/risk groups, three cities, and race/ethnicity (non-Hispanic white or not), ensuring balance in randomization for these three dimensions [27]. Within the strata of city, serostatus/risk group, and race/ethnicity, participants were randomly assigned to the next treatment allocation from a randomly permuted block sequence (block sizes were 2 and 4).

#### Follow-Up Assessments

To understand the effects of the intervention, follow-up surveys were administered to all participants in three-month intervals (a survey immediately postintervention, a three-month postintervention follow-up, and a six-month postintervention follow-up). The content of the postintervention surveys included study outcomes, which included sexual risk behaviors and use of prevention services. Participants took interim follow-up assessment remotely at 3 and 6 months and were given the option of taking the final 9-month outcome assessment remotely or in person as the baseline was done.

#### Incentives

Modest incentives were given to participants for completing assessments in the study. Participants in the intervention group could be compensated up to US $140 when completing all assessments over the nine-month study period. Participants in the waitlist-control group were compensated up to US $190 when opting to use the intervention app after nine months, and US $140 if they chose not to use the intervention app. All consenting participants who completed in-person site visits, baseline visits, and nine-month follow-up were compensated US $50, and an additional US $20 for each remotely completed follow-up survey (immediately postintervention, after three months, and after six months). Waitlist-control participants who opted to use the intervention app were eligible for an additional US $50 postintervention follow-up assessment incentive.

### Measures

#### Serostatus-Specific Measures

##### HIV-Negative Participants

For HIV-negative participants, HIV testing was assessed at baseline using a series of questions about HIV screening in the previous 12-month and three-month intervals, including reasons for screening behavior. Follow-up surveys asked about HIV screening behaviors in the past three months. HIV-negative participants were also asked a series of questions related to PrEP, including if they were aware of PrEP, had ever used PrEP, were currently using PrEP, or had discontinued PrEP. Reasons for use or nonuse of PrEP were also included at baseline and in three-month follow-up intervals. HIV-negative participants were also asked about the likelihood of using possible PrEP agents in the future. Additionally, participants were asked to rank their prevention preferences when considering PrEP options that might be available as future PrEP formulations.

##### HIV-Positive Participants

Participants living with HIV were asked at baseline about their previous 12 months of engagement with HIV care, and at follow-up about their previous 3 months of engagement with HIV care. Questions included missed appointments, reasons for nonengagement, and measures of viral suppression. Participants living with HIV were also asked at baseline about their use of ART in the previous 12-month period and previous 30-day adherence. Postintervention surveys asked about previous three-month initiation of ART and past 30-day adherence to ART, if applicable. Questions also included reasons for missed doses as well as the HIV Self-Efficacy Adherence Scale [28].

#### Questions Asked of All Participants

STI testing was assessed at baseline by asking participants if they had been screened in the previous 12-month period for non-HIV STIs, reasons for screening behavior, and any STI-related vaccinations. STIs for analysis included chlamydia, gonorrhea, syphilis, herpes, genital warts, hepatitis A, B, and C, and any other STI. Follow-up surveys asked information about screening and diagnoses in the previous three months. All participants were asked at baseline and in follow-up surveys about their recent experiences in accessing health care. All participants were asked about their beliefs on the efficacy of various HIV prevention methods (eg, condoms, PrEP) as well as the likelihood they would engage in HIV prevention behaviors in the next three-month period. All participants were asked about sexual behaviors in the previous three-month period at baseline and during three-month follow up surveys. Questions delineated main from casual partners, enumerating casual partners, and determining patterns of behavior for ongoing risk group classification. Participants were asked at baseline and in all postintervention surveys about substance use and dependency. Alcohol misuse was assessed using the validated Alcohol Use Disorders Identification Test (AUDIT) [29]. Participants were also asked which, if any, substances other than alcohol they used. Substance dependency was assessed with the Drug Use Dependency Identification Test (DUDIT) [30].

#### Covariates

Factors that might be associated with intervention efficacy were assessed at baseline and at three-month intervals. These included the Centers for Epidemiological Studies on Depression 10-item (CESD-10) scale [31] for past-week depressive symptoms, a modified technology use scale from a 2015 Pew report [32], lifetime and previous three-month sex work, previous three-month intimate partner violence (IPV), previous-month resilience, a subset of items from the HIV-related Stigma Scale, a modified HIV/AIDS Conspiracy Scale [33], Perceived HIV Severity Scale [34], and Health Care Mistrust Scale [35].

### Data Analysis Plan

We will conduct bivariate analyses, stratified by intervention group, for characteristics related to demographic factors, educational history, social determinants of health, city of enrollment, risk group, and baseline behaviors, to assess for failure of randomization. Failure of randomization will be defined by a significant (*P*<.05) difference in the distribution of a characteristic between the intervention and control groups.

The primary outcomes of interest in this study (Table 2) will be associated with the following alternative hypotheses: assignment to the intervention group will be associated with increased HIV testing among HIV-negative men, increased engagement with HIV care and ART use/adherence among people living with HIV, increased uptake and adherence to PrEP among higher risk HIV-negative men, sustained lack of condomless anal sex among lower risk HIV-negative men, increased condom use for those who reported prior condomless sex, and increased STI testing for all sexually active men. Assuming that there are no failures of randomization, we will use descriptive analyses to calculate the ratios of rates or prevalence between participants assigned to the intervention group and participants assigned to the control group. For HIV testing, PrEP uptake, and STI testing, we will consider using descriptive methods for time-to-event analysis and describing unadjusted hazard ratios if randomization does not fail. If a failure of randomization occurs, we will conduct a stratified analysis for the association between outcomes and randomization assignment by variable where the failure occurred and will consider adjusting estimates for that factor if indicated.

**Table 2:** Primary outcomes.

Outcome	Participant HIV risk group	
	Lower risk and higher risk HIV-negative	HIV-positive
HIV testing	✓	
Engagement in HIV preventative care	✓	
PrEP^a^ uptake	✓	
PrEP adherence	✓	
Engagement in HIV care		✓
ART^b^ uptake		✓
ART adherence		✓
Condom use	✓	✓
Condom use compliance	✓	✓
STI^c^ testing	✓	✓

^a^PrEP: pre-exposure prophylaxis.

^b^ART: antiretroviral therapy.

^c^STI: sexually transmitted infection.

Secondary outcomes will focus on participants’ self-reported intentions, such as increasing the frequency of preventive behaviors, decreasing risk behavior, seeking information and treatment, and partner communication, particularly around risk and prevention behaviors such as ART and PrEP. Secondary outcome analysis will include intervention and control group comparisons, similar to primary outcome analysis.

### Human Subjects

Study procedures and documents, including consent forms, eligibility screeners, assessments, recruitment advertisements, and sexual health messages, were submitted and approved by Emory University’s Institutional Review Board. All study staff were required to complete training in human subjects research ethics and data security before they were permitted to engage in research procedures and view participant identification information. Any third parties contracted in the data collection and management process have established Business Associates Agreements with Emory University, to ensure their adherence to the scientific and ethical standards of the university. A data safety monitoring board was not required for this study, as it poses no greater than minimal risk due to the limited safety concerns to the study population.

## Results

M3 enrollment began in January 2018 and concluded in November 2018. A total of 1229 MSM were enrolled. Data collection was completed in September 2019.

## Discussion

Eisinger and Fauci have recently written that we have the tools to end the HIV/AIDS pandemic, and that the remaining challenge is to aggressively implement the effective strategies that we have [36]. We agree and believe that the same premise applies to HIV prevention: that prevention tools, including HIV testing, condoms, STI testing, PrEP, and postexposure prophylaxis, offer significant promise to curb new infections among MSM if deployed at scale and aligned with the needs of MSM. Here, we describe an RCT to test the effects of a mobile phone app to help MSM select, coordinate, and manage their use of prevention tools. The design incorporates both the previously described HealthMindr app and a set of text and video messages that are tailored to the HIV status and the risk level of participants.

The resulting M3 mobile app provides tailored electronic messages along with service offerings in a unified platform, potentially increasing the uptake of primary prevention interventions. It features several characteristics of best practice in the development of mHealth tools: it is theoretically grounded, was developed through an iterative process with input from likely end users and current prevention providers, and uses tailoring of content based on the specific needs and circumstances of users. If assignment to the intervention is associated with higher use of prevention services, the M3 app could be used by public health agencies to reach MSM with a consistent, epidemiologically tailored package of HIV prevention interventions, and could provide an opportunity to reach men who might be geographically less accessible for existing, in-person prevention services. The inclusion of mail-out condoms and STI and HIV testing commodities also facilitates the provision of a full package of basic services to rural MSM, who are a group that are consistently less served with basic sexual health services and commodities than urban MSM [37]. A further potential benefit of this approach is the HIV serostatus-inclusiveness of the intervention: all men can potentially benefit from the M3 app, regardless of HIV serostatus.

Our study is subject to a number of possible limitations, typical of the potential biases associated with randomized prevention trials [38]. It is possible that we might have selection bias, manifested as a failure of randomization. We have taken steps to mitigate this possibility by implementing stratified randomization by site and risk group; stratified randomization minimizes the risk for failure of randomization across a domain that might be associated with efficacy. We will assess failure of randomization by comparing the distribution of key participant characteristics by randomization arm, using chi-square or Fisher exact tests as appropriate. We anticipate that there could be measurement bias because we are relying on self-reported outcomes, as is common in behavioral studies. We have attempted to minimize this bias by allowing options for completing assessments privately or remotely, by using previously established or validated items, and by measuring key outcomes with multiple measures (for example, adherence is assessed in both 7-day and 30-day recall periods, and assesses condom use in the past 3 months and at last sexual encounter). We could be subject to exclusion bias if there is differential loss to follow-up between the intervention and control arms. We mitigated this risk by using proven approaches to increase retention in both arms, including multiple modes of contact (phone, email, text message) and offering flexible options for completing surveys at home or in the research clinic. Because we set quotas to ensure adequate representation of men of color and high- and low-risk HIV-negative men, our sample may not have high external generalizability; however, we have designed the study to have power to analyze within specific, high-priority groups of participants, given their importance in HIV epidemics among MSM living in the United States. Because of the recruitment structure, cross-sectional analyses of the levels of prevention and risk behaviors from the baseline data should be interpreted in light of the sampling strategy.

Future study activities include completion of data collection for the RCT, analysis of RCT data, and identification of possible areas of improvement of the app for usability, including the assessment of use patterns of waitlist-control participants. In parallel, it is important that conversations with possible implementers of electronic health interventions (eg, local and state health departments and community-based prevention organizations) continue to anticipate possible implementation strategies and opportunities based on the principles of implementation science, in case the results of the RCT indicate improved outcomes for men randomized to the intervention group [39].

## Appendix

Multimedia Appendix 1 Baseline survey for the M3 (M-Cubed) study.

## Abbreviations

ART: antiretroviral therapy
AUDIT: Alcohol Use Disorders Identification Test
CESD-10: Centers for Epidemiological Studies on Depression 10-item scale
DUDIT: Drug Use Dependency Identification Test
IPV: intimate partner violence
M-Cubed/M3: Mobile Messaging for Men
mHealth: mobile health
MSM: men who have sex with men
nPEP: nonoccupational postexposure prophylaxis
PrEP: pre-exposure prophylaxis
RCT: randomized controlled trial
STI: sexually transmitted infection
