# Supplementary material for: HIV Prevention Via Mobile Messaging for Men Who Have Sex With Men (M-Cubed): Protocol for a Randomized Controlled Trial
Source: JMIR Res Protoc. 2019 Nov 15;8(11):e16439. doi: 10.2196/16439 (PMC6884718; doi:10.2196/16439)
Supplement: Multimedia Appendix 1 [file resprot_v8i11e16439_app1.pdf]

M3 Assessment - Baseline

ID and Initials

Hidden Value: sguid
Value: [url("sguid")]

Action: Custom Script: Split ID and Initials

Hidden Value: Study ID
Value:

Hidden Value: Initials
Value:

Hidden Value: City
Value:

Date Calculations

Action: Custom Script: Date Setup

Hidden Value: Date minus one year (DATE1YR)

Value:

Hidden Value: Date minus 3 months (DATE3MO)

Value:

Hidden Value: today_mo

Value:

Hidden Value: today_yr

Value:

Intro Instructions

ID: 845

Thank you for taking our survey today.

Helpful tips:

- **Questions marked with a red asterisk (*) are required questions that you must answer to move forward.**
- **You may skip any questions that you do not feel comfortable answering.**
- **This is a forward-only survey. When you finish a page, proceed to the next page by clicking the "Next" button. You may not go backwards to pages you already complete. Please do not use the back button on your browser as this may cause problems with your survey.**
- **We are going to be asking questions about your background and life experience, healthcare, HIV prevention, your sexual partners and relationships, alcohol and substance use, and more.**

Your confidentiality is important to us! All information you provide today in this survey will be held confidentially. Your answers will be used only for research purposes and we do not collect information to identify you and link you to your answers. Researchers will only see data that is anonymous.

Click the "Continue" button to get started with the survey.

Sociodemographic Variables (D1 - D5)

Logic: Show/hide trigger exists.

Shortname / Alias: origin

ID: 741

Were you born in the United States?

(1) Yes

(0) No

(7) I don't know

Logic: Hidden unless: Question "Were you born in the United States?" is one of the following answers ("No")

Shortname / Alias: origin_yr

ID: 743

What year did you first come to the United States?

( ) 2017

( ) 2016

( ) 2015

( ) 2014

( ) 2013

( ) 2012

( ) 2011
( ) 2010
( ) 2009
( ) 2008
( ) 2007
( ) 2006
( ) 2005
( ) 2004
( ) 2003
( ) 2002
( ) 2001
( ) 2000
( ) 1999
( ) 1998
( ) 1997
( ) 1996
( ) 1995
( ) 1994
( ) 1993
( ) 1992
( ) 1991
( ) 1990
( ) 1989
( ) 1988
( ) 1987
( ) 1986
( ) 1985
( ) 1984
( ) 1983
( ) 1982
( ) 1981
( ) 1980
( ) 1979

( ) 1978
( ) 1977
( ) 1976
( ) 1975
( ) 1974
( ) 1973
( ) 1972
( ) 1971
( ) 1970

Shortname / Alias: language
ID: 744

What language do you speak at home?

(1) English
(2) Spanish
(3) Arabic
(4) Chinese
(5) Italian
(6) Korean
(7) Russian
(8) Vietnamese
(9) Other, please specify:: ____

Sociodemographic Variables (D6 - D10)

Shortname / Alias: school
ID: 26

Are you currently enrolled in school?

(1) Yes, full-time

(2) Yes, part-time

(0) No

Shortname / Alias: educ

ID: 27

What is the highest level of education that you have completed?

(1) Less than high school

(2) High school graduate or obtained GED

(3) Some college, Associate's Degree, or Technical Degree

(4) Bachelor's Degree / College Degree

(5) Any post-graduate studies

Shortname / Alias: work

ID: 28

Which of the following best describes your employment status? Are you:

(1) Employed full-time

(2) Employed part-time

(3) On active duty in the U.S. Armed forces, Reserves, or National Guard

(4) Retired

(5) Unable to work for health reasons

(6) Unemployed

(7) Other, please specify:: ____

Shortname / Alias: income

ID: 29

What was your income last year from all sources before taxes?

(1) $0 to $4,999

(2) $5,000 to $9,999

(3) $10,000 to $14,999

(4) $15,000 to $19,999

(5) $20,000 to $29,999

(6) $30,000 to $39,999

(7) $40,000 to $49,999

(8) $50,000 to $74,999

(9) $75,000 or more

Sociodemographic (D11 - D14)

Shortname / Alias: orient

ID: 233

Which of the following best represents how you think of yourself?

(1) Gay or homosexual

(2) Straight or heterosexual

(3) Bisexual

(4) Other, please specify:: ____

Logic: Show/hide trigger exists.

Shortname / Alias: homeless

ID: 30

In the past 12 months, that is since [question('value'),id='759'], have you been homeless at any time?

By homeless, we mean living on the street, in a shelter, a Single Room Occupancy hotel (SRO), temporarily staying with friends or relatives, or living in a car.

(1) Yes

(0) No

Logic: Hidden unless: Question "In the past 12 months, that is since [question('value'),id='759'], have you been homeless at any time?

By homeless, we mean living on the street, in a shelter, a Single Room Occupancy hotel (SRO), temporarily staying with friends or relatives, or living in a car." is one of the following answers ("Yes")

Shortname / Alias: homeless_now

ID: 31

Are you currently homeless?

(1) Yes

(0) No

Sociodemographic (D15 - D19)

Logic: Show/hide trigger exists.

Shortname / Alias: ins

ID: 32

Do you currently have health insurance or health care coverage (including Medicare or Medicaid)?

(1) Yes

(0) No

Logic: Hidden unless: Question "Do you currently have health insurance or health care coverage (including Medicare or Medicaid)?" is one of the following answers ("Yes")

Shortname / Alias: ins_type

ID: 33

What kind of health insurance or coverage do you currently have?

(1) A private health plan (through an employer or purchased directly)

(2) Medicaid (for people with low incomes)

(3) Medicare (for elderly people and people with disabilities)

(4) Some other government plan (e.g., ADAP, a state government plan)

(5) TRICARE (CHAMPUS)

(6) Veteran Administration coverage

(7) Some other health insurance plan, please specify::

____

Logic: Show/hide trigger exists.

Shortname / Alias: jail

ID: 34

Have you ever been in jail or prison?

(1) Yes

(0) No

Logic: Hidden unless: Question "Have you ever been in jail or prison?" is one of the following answers ("Yes")

Shortname / Alias: jail_3m

ID: 35

In the past 3 months, that is since [question('value'),id='760'], have you been in jail or prison?

(1) Yes

(0) No

HIV Testing (HT3)

Shortname / Alias: evertest

ID: 37

Have you ever been tested for HIV?

(1) Yes

(0) No

(7) I don't know

Page entry logic: This page will show when: Question "Have you ever been tested for HIV?" is one of the following answers ("Yes")

HIV Testing (HT4 - HT7)

Shortname / Alias: firsttest_yr

ID: 501

When was your first HIV test?

Year:

( ) 2018

( ) 2017

( ) 2016

( ) 2015

( ) 2014

( ) 2013
( ) 2012
( ) 2011
( ) 2010
( ) 2009
( ) 2008
( ) 2007
( ) 2006
( ) 2005
( ) 2004
( ) 2003
( ) 2002
( ) 2001
( ) 2000
( ) 1999
( ) 1998
( ) 1997
( ) 1996
( ) 1995
( ) 1994
( ) 1993
( ) 1992
( ) 1991
( ) 1990
( ) 1989
( ) 1988
( ) 1987
( ) 1986
( ) 1985

**Logic: Show/hide trigger exists.**
Shortname / Alias: hivtest_12m

ID: 39

In the past 12 months, that is since [question('value'),id='759'], have you received an HIV test?

(1) Yes

(0) No

Logic: Hidden unless: Question "In the past 12 months, that is since [question('value'),id='759'], have you received an HIV test?" is one of the following answers ("Yes")

Shortname / Alias: hivtest_3m

ID: 41

In the past 3 months, that is since [question('value'),id='760'], have you received an HIV test?

(1) Yes

(0) No

Shortname / Alias: test_result

ID: 42

What was the result of your most recent HIV test?

(0) Negative

(1) Positive

(2) I never received the results

(3) Indeterminate/Inconclusive

(7) I don't know

HIV Status

Logic: Show/hide trigger exists.

Shortname / Alias: hivstatus

ID: 751

What is your HIV status?*

(0) HIV Negative

(1) HIV Positive

(2) Indeterminate

(7) I don't know

(9) I'd prefer not to answer

Page entry logic: This page will show when: Question "What is your HIV status?" is one of the following answers ("HIV Positive")

HIV Testing HT1

Shortname / Alias: hivpos_yr

ID: 476

In what year was your *first* positive test for HIV?

Year:

( ) 2020

( ) 2019

( ) 2018

( ) 2017

( ) 2016

( ) 2015

( ) 2014

( ) 2013

( ) 2012
( ) 2011
( ) 2010
( ) 2009
( ) 2008
( ) 2007
( ) 2006
( ) 2005
( ) 2004
( ) 2003
( ) 2002
( ) 2001
( ) 2000
( ) 1999
( ) 1998
( ) 1997
( ) 1996
( ) 1995
( ) 1994
( ) 1993
( ) 1992
( ) 1991
( ) 1990
( ) 1989
( ) 1988
( ) 1987
( ) 1986
( ) 1985

Script to calculate if 2 year HIV diag difference

Action: Custom Script: 2 previous years

Hidden Value: check_yr

Value:

Page entry logic: This page will show when: Question "What is your HIV status?" is one of the following answers ("HIV Positive")

HIV Testing (HT2)

Page exit logic: Skip / Disqualify Logic**IF:** Question "What is your HIV status?" is one of the following answers ("HIV Positive") **THEN:** Jump to page 18 - STI Testing (ST1 - ST4)

Logic: Hidden unless: check_yr is less than or equal to "2"

Shortname / Alias: hivpos_12m

ID: 483

In the past 12 months, that is since [question('value'), id='759'], did you receive your first positive test for HIV?

(1) Yes

(0) No

Page entry logic: This page will show when: (Question "What is your HIV status?" is one of the following answers ("HIV Negative","Indeterminate","I don't know") AND Question "Have you ever been tested for HIV?" is one of the following answers ("Yes"))

HIV Testing (HT8 - HT11)

Shortname / Alias: test_where

ID: 43

When you last got tested for HIV, where did you get tested?

(1) Private doctor's office

(2) Community health center/ public health clinic

(3) HIV counseling and testing site

(4) HIV/AIDS street outreach program/ Mobile testing unit

(5) Hospital (inpatient)

(6) Emergency Room

(7) Sexually transmitted disease clinic

(8) Drug treatment program

(9) Correctional facility (jail or prison)

(10) Blood bank/ plasma center

(11) Military

(12) At home

(13) Other, please specify:: ____

Shortname / Alias: test_why

ID: 410

When you last got tested for HIV, what prompted you to get tested? Select all that apply.

[1] The condom broke [TEST_WHY_BROKE]

[2] I did not use a condom the last time I had sex [TEST_WHY_CONDOM]

[3] I was drunk or high the last time I had sex and I don't remember exactly what happened [TEST_WHY_DRUGS]

[4] I found out a partner I had anal sex with was HIV-positive [TEST_WHY_POS]

[5] The HIV test was part of a research study [TEST_WHY_STUDY]

[6] I was already at the doctor's office [TEST_WHY_DOC]

[7] I was due for an HIV test [TEST_WHY_ROUTINE]

[8] I wanted to know my status [TEST_WHY_KNOW]

[9] My partner encouraged me to get tested [TEST_WHY_ENCOURAGE]

[10] My partner got tested, so I did too [TEST_WHY_PARTNER]

[11] It's a condition of my sexual agreement [TEST_WHY_AGREE]

[12] My partner and I wanted to be monogamous [TEST_WHY_MONO]

[13] I was beginning a relationship [TEST_WHY_RLSHIP]

[14] I was feeling sick [TEST_WHY_SICK]

[15] Someone I had sex with was feeling sick/got sick [TEST_WHY_PSICK]

[16] I shared a needle with someone else [TEST_WHY_NEEDLE]

[17] I had a lot of risky sex [TEST_WHY_RISKY]

[18] I had a lot of sex partners [TEST_WHY_LOTS]

[19] Other, please specify: ____
[TEST_WHY_OTHER, TEST_WHY_OTHERSP]

Page entry logic: This page will show when: Question "What is your HIV status?" is one of the following answers ("HIV Negative","Indeterminate","I don't know")

HIV Testing (HT12 - HT14)

Logic: Hidden unless: (Question "Have you ever been tested for HIV?" is not one of the following answers ("Yes") OR Question "In the past 12 months, that is since [question('value'),id='759'], have you received an HIV test?" is not one of the following answers ("Yes"))

Shortname / Alias: test_whynot_12m

ID: 413

Which of the following reasons best describes why you have not been tested for HIV in the past 12 months:

(1) I think I am at low risk for HIV infection

(2) I am afraid of finding out I have HIV

(3) I didn't have time

(4) I didn't know where to get tested

(5) My doctor hasn't brought it up

(6) I am worried about the cost

(7) No particular reason

(8) Other, please specify:: ____

Shortname / Alias: test_often

ID: 46

How often do you think you should be tested for HIV?

(1) Less than once a year

(2) Once a year

(3) Every 6 months

(4) Every 3 months

(5) Every month

(6) Other, please specify: ____

Page entry logic: This page will show when: Question "What is your HIV status?" is one of the following answers ("HIV Negative","Indeterminate","I don't know")

HIV Testing (HT15 - HT16)

Logic: Show/hide trigger exists.

Shortname / Alias: test_next

ID: 47

When do you plan to next get tested for HIV?

(1) Within the next month

(2) Within the next 1 to 3 months

(3) Within the next 4 to 6 months

(4) Within the next 7 to 12 months

(5) More than a year from now

(6) I'm not planning to get tested

(77) I don't know

Logic: Hidden unless: Question "When do you plan to next get tested for HIV?" is one of the following answers ("More than a year from now","I'm not planning to get tested")

Shortname / Alias: test_notnext12m

ID: 48

Which of the following reasons best describes why you do not plan to be tested in the next 12 months:

(1) I think I am at low risk for HIV infection

(2) I am afraid of finding out I have HIV

(3) I don't have time

(4) I don't know where to get tested

(5) I am worried about the cost

(6) No particular reason

(7) Other, please specify:: ____

Page entry logic: This page will show when: Question "What is your HIV status?" is one of the following answers ("HIV Negative","Indeterminate","I don't know")

HIV Testing (HT17-HT18)

Logic: Hidden unless: Question "When do you plan to next get tested for HIV?" is one of the following answers ("Within the next month","Within the next 1 to 3 months","Within the next 4 to 6 months","Within the next 7 to 12 months")

Shortname / Alias: test_next12m

ID: 49

Which of the following reasons best describes why you plan to be tested [question("option title"), id="47",case="lower"]:

(1) I have been feeling sick

(2) Someone I had sex with has been feeling sick/is sick

(3) I found out a partner I had anal sex with is HIV-positive

(4) I am due for an HIV test

(5) I want to know my status

(6) It's a condition of my sexual agreement

(7) My partner and I want to be monogamous

(8) I am beginning a relationship

(9) I often share needles

(10) I have a lot of risky sex

(11) I have a lot of sex partners

(12) Other, please specify:: ____

STI Testing (ST1 - ST4)

Shortname / Alias: stitest_3m

ID: 50

In the past 3 months, that is since [question('value'),id='760'], have you been tested by a doctor, nurse or other health care provider for any sexually transmitted diseases (i.e., STDs or STIs)? This does not include tests for HIV.

(1) Yes

(0) No

Shortname / Alias: vacc_hpv

ID: 61

A vaccine to prevent HPV infection is available and is called the HPV shot or GARDISIL. Have you ever received the HPV shot?

(1) Yes

(0) No

(7) I don't know

Logic: Show/hide trigger exists.

Shortname / Alias: vacc_hep

ID: 62

There are vaccines or shots that can prevent some types of hepatitis. Have you ever had a hepatitis vaccine?

(1) Yes

(0) No

(7) I don't know

Logic: Hidden unless: Question "There are vaccines or shots that can prevent some types of hepatitis. Have you ever had a hepatitis vaccine?" is one of the following answers ("Yes")

Shortname / Alias: hep_type

ID: 63

What type or types of hepatitis vaccine have you had?

(1) Hepatitis A vaccine

(2) Hepatitis B vaccine

(3) Both the Hepatitis A and B vaccines

(7) I don't know

STI Testing (ST5 - ST8)

Page exit logic: Skip / Disqualify Logic**IF:** Question "What is your HIV status?" is one of the following answers ("HIV Negative","Indeterminate","I don't know") **THEN:** Jump to page 31 - PrEP Uptake/ Adherence (PA1 - PA4)

Shortname / Alias: sti_

ID: 684

In the past 3 months, that is since [question('value'),id='760'], has a doctor, nurse or other health care provider told you that you had any of the following STDs?

	Yes (1)	No (0)	I don't know (7)
Chlamydia [STI_CHLAMYDIA]	( )	( )	( )
Gonorrhea [STI_GONORRHEA]	( )	( )	( )
Syphilis [STI_SYPHILIS]	( )	( )	( )
Herpes [STI_HERPES]	( )	( )	( )
Genital Warts [STI_WARTS]	( )	( )	( )
Hepatitis A [STI_HEPA]	( )	( )	( )
Hepatitis B [STI_HEPB]	( )	( )	( )
Hepatitis C [STI_HEPC]	( )	( )	( )

Some other STD [STI_OTHER]	( )	( )	( )

Logic: Hidden unless: Question "Some other STD" is one of the following answers ("Yes")

Shortname / Alias: sti_othersp

ID: 60

Please specify which other STD(s):

____

Shortname / Alias: sti_

ID: 64

When do you think you should be tested for an STD? Select all that apply.

[1] When I have symptoms of an STD

[2] When I've had sex without a condom

[3] When my partner tests positive for an STD

[4] When it's time for my routine test

[6] When a doctor or other healthcare provider tells me I should be tested

[7] Other, please specify:: ____

Page entry logic: This page will show when: Question "What is your HIV status?" is one of the following answers ("HIV Positive")

HIV Care Engagement (HC1 - HC3)

Logic: Show/hide trigger exists.

Shortname / Alias: hivcare_12m

ID: 66

In the past 12 months, that is since [question('value'),id='759'], have you seen a doctor, nurse or other health provider for outpatient HIV care?

(1) Yes

(0) No

Validation: Max = 999 Must be numeric Whole numbers only Positive numbers only

Logic: Hidden unless: Question "In the past 12 months, that is since [question('value'),id='759'], have you seen a doctor, nurse or other health provider for outpatient HIV care?" is one of the following answers ("Yes")

Shortname / Alias: appointnum

ID: 562

In the past 12 months, that is since [question('value'),id='759'], how many appointments have you scheduled with a doctor, nurse or other health provider for HIV care?

____

HIV Care Engagement (HC3)

Logic: Hidden by default

ID: 818

The total number of attended appointments in the past 12 months must be less than or equal to [question('value'), id='562'] (the number of scheduled appointments in the past 12 months). Please correct this.

Validation: Max = 999 Must be numeric Whole numbers only Positive numbers only

Logic: Hidden unless: Question "In the past 12 months, that is since [question('value'),id='759'], have you seen a doctor, nurse or other health provider for outpatient HIV care?" is one of the following answers ("Yes")

Shortname / Alias: attendnum

ID: 563

In the past 12 months, that is since [question('value'),id='759'], how many of your scheduled appointments did you attend?

____

Script to Determine if Attended Appts Greater than Scheduled Appts

Action: Custom Script: Attended Appts Greater than Scheduled Appts

---

Page entry logic: This page will show when: Question "In the **past 12 months**, that is since [question('value'),id='759'], have you seen a doctor, nurse or other health provider for outpatient HIV care?" is one of the following answers ("Yes")

Script to Calculate Missed Appointments

Action: Custom Script: missed appointments setup

Hidden Value: number of missed appointments [MISSEDAPPTS]

Value:

Page entry logic: This page will show when: number of missed appointments is not exactly equal to "0"

HIV Care Engagement (HC4 - HC5)

Logic: Hidden unless: number of missed appointments is greater than or equal to "1"

Shortname / Alias: attend_

ID: 419

Why did you not attend [question("value"),id="102"] of your scheduled appointments? Select all that apply.

[1] I was busy [ATTEND_BUSY]

[2] I simply forgot [ATTEND_FORGOT]

[3] I had a schedule change or traveling [ATTEND_SCHEDULE]

[4] My provider canceled our appointment [ATTEND_DOCTOR]

[5] I did not have access to transportation [ATTEND_TRANSPORT]

[6] I worried others would see/find out [ATTEND_FINDOUT]

[7] I did not think it was necessary [ATTEND_NECESSARY]

[8] I do not have insurance [ATTEND_INS]

[9] I was worried about receiving HIV care [ATTEND_CARE]

[10] I felt depressed and/or overwhelmed [ATTEND_SAD]

[11] I was drunk or high [ATTEND_DRUGS]

[12] Other, please specify:: ____ [ATTEND_OTHER] [ATTEND_OTHERSP]

HIV Care Engagement (HC6 - HC8)

Logic: Hidden unless: Question "In the past 12 months, that is since [question('value'),id='759'], have you seen a doctor, nurse or other health provider for outpatient HIV care?" is one of the following answers ("Yes")

Shortname / Alias: hivcare_3m

ID: 71

In the past 3 months, that is since [question('value'), id='760'], have you had a medical visit in an HIV care setting?

(1) Yes

(0) No

Logic: Show/hide trigger exists.

Shortname / Alias: vl_3m

ID: 72

To your knowledge, has your doctor or another provider ordered a viral load test for you in the past 3 months, that is since [question('value'), id='760']? This would have involved drawing your blood.

(1) Yes

(0) No

Validation: Must be numeric Whole numbers only Positive numbers only

Logic: Hidden unless: Question "To your knowledge, has your doctor or another provider ordered a viral load test for you in the past 3 months, that is since [question('value'), id='760']? This would have involved drawing your blood." is one of the following answers ("Yes")

Shortname / Alias: vl_

ID: 73

What was your viral load count the last time it was measured?

( ) # copies/ml: ____

(1) Undetectable

(7) I don't know

Page entry logic: This page will show when: Question "What is your HIV status?" is one of the following answers ("HIV Positive")

ART Uptake/Adherence (MA1 - MA2)

Page exit logic: Skip / Disqualify Logic**IF:** ((((((((((((Question "What are the reasons you have never taken any antiretroviral medications? Select all that apply." is exactly equal to ("My doctor advised to delay treatment") OR Question "What are the reasons you have never taken any antiretroviral medications? Select all that apply." is exactly equal to ("I recently entered medical care/haven't had time")) OR Question "What are the reasons you have never taken any antiretroviral medications? Select all that apply." is exactly equal to ("My CD4 and/or viral load are good")) OR Question "What are the reasons you have never taken any antiretroviral medications? Select all that apply." is exactly equal to ("I feel good and don't need them")) OR Question "What are the reasons you have never taken any antiretroviral medications? Select all that apply." is exactly equal to ("I'm worried about the side effects")) OR Question "What are the reasons you have never taken any antiretroviral medications? Select all that apply." is exactly equal to ("Because of my drinking or drug use")) OR Question "What are the reasons you have never taken any antiretroviral medications? Select all that apply." is exactly equal to ("I don't want to think about being HIV positive")) OR Question "What are the reasons you have never taken any antiretroviral medications? Select all that apply." is exactly equal to ("I don't have enough money")) OR Question "What are the reasons you have never taken any antiretroviral medications? Select all that apply." is exactly equal to ("I don't have health insurance")) OR Question "What are the reasons you have never taken any antiretroviral medications? Select all that apply." is exactly equal to ("I am worried about my ability to take my medicine, I often forget to take medications")) OR Question "What are the reasons you have never taken any antiretroviral medications? Select all that apply." is exactly equal to ("I'm living on the street")) OR Question "What are the reasons you have never taken any antiretroviral medications? Select all that apply." is exactly equal to ("I'm taking alternative or complementary medicine")) **THEN:** Jump to page 28 - ART Uptake/Adherence (MA4 - MA7)

Logic: Show/hide trigger exists.

Shortname / Alias: artever

ID: 74

Have you ever been prescribed and taken antiretroviral medications to treat your HIV?

(1) Yes

(0) No

(7) I don't know

Logic: Hidden unless: Question "Have you ever been prescribed and taken antiretroviral medications to treat your HIV?" is one of the following answers ("No")

Shortname / Alias: art_never

ID: 75

What are the reasons you have never taken any antiretroviral medications? Select all that apply.

[1] My doctor advised to delay treatment [ART_NEVER_DOC]

[1] I recently entered medical care/haven't had time [ART_NEVER_MEDCARE]

[1] My CD4 and/or viral load are good [ART_NEVER_CD4]

[1] I feel good and don't need them [ART_NEVER_GOOD]

[1] I'm worried about the side effects [ART_NEVER_EFFECTS]

[1] Because of my drinking or drug use [ART_NEVER_DRUGS]

[1] I don't want to think about being HIV positive [ART_NEVER_DENIAL]

[1] I don't have enough money [ART_NEVER_MONEY]

[1] I don't have health insurance [ART_NEVER_INS]

[1] I am worried about my ability to take my medicine, I often forget to take medications [ART_NEVER_ADHERE]

[1] I'm living on the street [ART_NEVER_HOMELESS]

[1] I'm taking alternative or complementary medicine [ART_NEVER_ALT]

[1] Other, please specify:: ____ [ART_NEVER_OTHER] [ART_NEVER_SPECIFY]

Page entry logic: This page will show when: (Question "What are the reasons you have never taken any antiretroviral medications? Select all that apply." is one of the following answers ("My doctor advised to delay treatment","I recently entered medical care/haven't had time","My CD4 and/or viral load are good","I feel good and don't need them","I'm worried about the side effects","Because of my drinking or drug use","I don't want to think about being HIV positive","I don't have enough money","I don't have health insurance","I am worried about my ability to take my medicine, I often forget to take medications","I'm living on the street","I'm taking alternative

or complementary medicine") AND Question "What is your HIV status?" is one of the following answers ("HIV Positive"))

ART Uptake/Adherence (MA3)

Shortname / Alias: art_never_main

ID: 421

Piping: Piped Values From Question (What are the reasons you have never taken any antiretroviral medications? Select all that apply.)

You said you've never taken any antiretroviral medications for the following reasons. Of these, which is the most important reason you've never taken antiretroviral medications?

Page entry logic: This page will show when: Question "What is your HIV status?" is one of the following answers ("HIV Positive")

ART Uptake/Adherence (MA4 - MA7)

Logic: Show/hide trigger exists. Hidden unless: Question "Have you ever been prescribed and taken antiretroviral medications to treat your HIV?" is one of the following answers ("Yes")

Shortname / Alias: art_current

ID: 76

Are you currently prescribed and taking any antiretroviral medicines to treat your HIV?

(1) Yes

(0) No

Logic: Hidden unless: Question "Are you currently prescribed and taking any antiretroviral medicines to treat your HIV?" is one of the following answers ("Yes")

Shortname / Alias: art_start

ID: 77

Did you start taking antiretroviral medicines to treat your HIV in the past 3 months? That is since [question('value'), id='760'].

(1) Yes

(0) No

Logic: Hidden unless: Question "Are you currently prescribed and taking any antiretroviral medicines to treat your HIV?" is one of the following answers ("Yes")

Shortname / Alias: art_stop

ID: 78

In the past 3 months, that is since [question('value'), id='760'], did you stop taking your antiretroviral medicines on purpose at any point in time? Do NOT include times when you accidentally missed one or more doses.

(1) Yes

(0) No

Logic: Hidden unless: Question "Are you currently prescribed and taking any antiretroviral medicines to treat your HIV?" is one of the following answers ("No")

Shortname / Alias: art_3m

ID: 79

In the past 3 months, that is since [question('value'), id='760'], have you taken any antiretroviral medicines to treat your HIV?

(1) Yes

(0) No

(7) I don't know

Page entry logic: This page will show when: (Question "Are you currently prescribed and taking any antiretroviral medicines to treat your HIV?" is one of the following answers ("Yes") AND Question "What is your HIV status?" is one of the following answers ("HIV Positive"))

ART Uptake / Adherence (MA8 - MA11)

Shortname / Alias: art_7d

ID: 80

In the past 7 days, on how many days did you take all of your doses of your HIV medicines?

( ) 7

( ) 6

( ) 5

( ) 4

( ) 3

( ) 2

( ) 1

( ) 0

Shortname / Alias: art_30d

ID: 81

In the past 30 days, on how many days did you take all all of your doses of your HIV medicines?

( ) 30

( ) 29

( ) 28

( ) 27

( ) 26

( ) 25

( ) 24

( ) 23

( ) 22

( ) 21

( ) 20

( ) 19

( ) 18

( ) 17

( ) 16

( ) 15

( ) 14

( ) 13

( ) 12

( ) 11

( ) 10

( ) 9

( ) 8

( ) 7

( ) 6

( ) 5

( ) 4

( ) 3

( ) 2

( ) 1

( ) 0

Shortname / Alias: art_good

ID: 82

In the past 30 days, how good a job did you do at taking your HIV medicines in the way you were supposed to?

(1) Very poor

(2) Poor

(3) Fair

(4) Good

(5) Very good

(6) Excellent

Shortname / Alias: art_often

ID: 83

In the past 30 days, how often did you take your HIV medicines in the way you were supposed to?

(1) Never

(2) Rarely

(3) Sometimes

(4) Usually

(5) Almost always

(6) Always

Page entry logic: This page will show when: (Question "What is your HIV status?" is one of the following answers ("HIV Positive") AND Question "Are you currently prescribed and taking any antiretroviral medicines to treat your HIV?" is one of the following answers ("Yes"))

ART Uptake/Adherence (MA12)

Page exit logic: Skip / Disqualify Logic**IF:** Question "What is your HIV status?" is one of the following answers ("HIV Positive") **THEN:** Jump to page 46 - General Care Engagement (GC1 - GC2)

Shortname / Alias: adherence_

ID: 84

In the next 30 days, please indicate how confident you feel that you will be able to carry out the following:

	Extremely Unconfident (1)	Somewhat Unconfident (2)	Uncertain (3)	Somewhat Confident (4)	Extremely Confident (5)
Stick with your treatment plan even when side effects begin to interfere with daily activities [ADHERENCE_1]	( )	( )	( )	( )	( )
Integrate your treatment into your daily routine [ADHERENCE_2]	( )	( )	( )	( )	( )
Integrate your treatment into your daily routine, even if it means taking medication or doing other things in front of people who don't know you have HIV [ADHERENCE_3]	( )	( )	( )	( )	( )
Stick to your treatment schedule even if your daily routine is disrupted [ADHERENCE_4]	( )	( )	( )	( )	( )

Stick to your treatment schedule when it means changing your eating habits [ADHERENCE_5]	( )	( )	( )	( )	( )
Continue with your treatment even if it interferes with daily activities [ADHERENCE_6]	( )	( )	( )	( )	( )
Continue with the treatment plan your doctor prescribed even if your t-cells drop significantly [ADHERENCE_7]	( )	( )	( )	( )	( )
Continue with your treatment even when you are feeling discouraged about your health [ADHERENCE_8]	( )	( )	( )	( )	( )
Continue with your treatment even when getting to the clinic appointments is a major hassle [ADHERENCE_9]	( )	( )	( )	( )	( )
Continue with your treatment	( )	( )	( )	( )	( )

even when people close to you tell you that they don't think that it is doing any good [ADHERENCE_10]					
Get something positive out of your participation in treatment, even if medication you are taking does not improve your health [ADHERENCE_11]	( )	( )	( )	( )	( )

Page entry logic: This page will show when: Question "What is your HIV status?" is one of the following answers ("HIV Negative","Indeterminate","I don't know")

PrEP Uptake/ Adherence (PA1 - PA4)

Logic: Show/hide trigger exists.

Shortname / Alias: prep

ID: 104

There is a pill HIV-negative men can take to reduce their risk of HIV. This is called pre-exposure prophylaxis, or PrEP. Have you ever heard of PrEP?

(1) Yes

(0) No

Logic: Show/hide trigger exists. Hidden unless: Question "There is a pill HIV-negative men can take to reduce their risk of HIV. This is called pre-exposure prophylaxis, or PrEP. Have you ever heard of PrEP?" is one of the following answers ("Yes")

Shortname / Alias: prep_current

ID: 105

Are you currently taking PrEP to prevent HIV?

(1) Yes

(0) No

(7) I don't know

Logic: Hidden unless: Question "Are you currently taking PrEP to prevent HIV?" is one of the following answers ("Yes")

Shortname / Alias: prep_start

ID: 106

Did you start taking PrEP in the past 3 months? That is since [question('value'), id='760'].

(1) Yes

(0) No

Logic: Hidden unless: Question "Are you currently taking PrEP to prevent HIV?" is one of the following answers ("Yes")

Shortname / Alias: prep_stop

ID: 107

In the past 3 months, did you stop taking PrEP on purpose for any period of time? Do not include times when you accidentally missed one or more doses of PrEP.

(1) Yes

(0) No

Page entry logic: This page will show when: (Question "Are you currently taking PrEP to prevent HIV?" is one of the following answers ("No") AND Question "What is your HIV status?" is one of the following answers ("HIV Negative","Indeterminate","I don't know"))

PrEP Update / Adherence (PA5 - PA8)

Logic: Show/hide trigger exists.

Shortname / Alias: prep_stop_3m

ID: 108

In the past 3 months, that is since [question('value'), id='760'], were you taking PrEP but stopped?

(1) Yes

(0) No

Logic: Hidden unless: Question "In the past 3 months, that is since [question('value'), id='760'], were you taking PrEP but stopped?" is one of the following answers ("No")

Shortname / Alias: prepever

ID: 109

Have you ever used PrEP to prevent HIV?

(1) Yes

(0) No

Logic: Hidden unless: (Question "In the past 3 months, that is since [question('value'), id='760'], were you taking PrEP but stopped?" is one of the following answers ("Yes") OR Question "Have you ever used PrEP to prevent HIV?" is one of the following answers ("Yes"))

Shortname / Alias: prep_stop_

ID: 110

What are some of the reasons you stopped using PrEP to prevent HIV? Select all that apply.

[1] I couldn't afford it [PREP_STOP_MONEY]

[1] My insurance wouldn't cover it [PREP_STOP_INS]

[1] My doctor decided PrEP was no longer appropriate for me [PREP_STOP_DOCTOR]

[1] I didn't like the side effects [PREP_STOP_EFFECTS]

[1] I was worried about the safety of PrEP [PREP_STOP_SAFE]

[1] My kidney and liver function began to suffer [PREP_STOP_LIVER]

[1] I started a committed relationship [PREP_STOP_RELATIONSHIP]

[1] I was using PrEP in a previous relationship with a person living with HIV [PREP_STOP_PARTPOS]

[1] I didn't think it was worth it [PREP_STOP_WORTH]

[1] I contracted HIV [PREP_STOP_HIV]

[1] It was too inconvenient/didn't fit my life [PREP_STOP_LIFE]

[1] I was worried someone would think I had HIV if they saw me take it [PREP_STOP_FINDOUT]

[1] I decided I didn't need it anymore [PREP_STOP_DONTNEED]

[1] Other, please specify:: ____ [PREP_STOP_OTHER] [PREP_STOP_OTHERSP]

Reasons Never Used PrEP

Logic: Hidden unless: (Question "Are you currently taking PrEP to prevent HIV?" is one of the following answers ("No") AND Question "Have you ever used PrEP to prevent HIV?" is one of the following answers ("No"))

Shortname / Alias: prep_never_

ID: 111

What are some of the reasons you've never used PrEP? Select all that apply.

[1] I couldn't afford it [PREP_NEVER_MONEY]

[1] My insurance wouldn't cover it [PREP_NEVER_INS]

[1] I didn't know where to get it [PREP_NEVER_ACCESS]

[1] My doctor did not recommend PrEP for me [PREP_NEVER_DOCTOR]

[1] I was worried about the side effects [PREP_NEVER_EFFECTS]

[1] I started a committed relationship [PREP_NEVER_RELATIONSHIP]

[1] I didn't think it was worth it [PREP_NEVER_WORTH]

[1] I contracted HIV [PREP_NEVER_HIV]

[1] It was too inconvenient/didn't fit my life [PREP_NEVER_LIFE]

[1] I was worried someone would think I had HIV if they saw me take it [PREP_NEVER_FINDOUT]

[1] I decided I didn't need it [PREP_NEVER_DONTNEED]

[1] I have never heard of it [PREP_NEVER_HEARD]

[1] Other, please specify:: ____ [PREP_NEVER_OTHER] [PREP_NEVER_OTHERSP]

(untitled)

Hidden Value: CheckboxTotal

Value: sgapiCheckboxTotalChecked(111)

Action: Custom Script: New Custom Script

Page entry logic: This page will show when: Question "What are some of the reasons you've never used PrEP? Select all that apply." is one of the following answers ("I couldn't afford it","My insurance wouldn't cover it","I didn't know where to get it","My doctor did not recommend PrEP for me","I was worried about the side effects","I started a committed relationship","I didn't think it was worth it","I contracted HIV","It was too inconvenient/didn't fit my life","I was worried someone would think I had HIV if they saw me take it","I decided I didn't need it","I have never heard of it","Other, please specify:")

PrEP Uptake/Adherence (PA12)

Page exit logic: Skip / Disqualify Logic**IF:** (((((((((((((((Question "What are some of the reasons you stopped using PrEP to prevent HIV? Select all that apply." is exactly equal to ("I couldn't afford it") OR Question "What are some of the reasons you stopped using PrEP to prevent HIV? Select all that apply." is exactly equal to ("My insurance wouldn't cover it")) OR Question "What are some of the reasons you stopped using PrEP to prevent HIV? Select all that apply." is exactly equal to ("My doctor decided PrEP was no longer appropriate for me")) OR Question "What are some of the reasons you stopped using PrEP to prevent HIV? Select all that apply." is exactly equal to ("I didn't like the side effects")) OR Question "What are some of the reasons you stopped using PrEP to prevent HIV? Select all that apply." is exactly equal to ("I was worried about the safety of PrEP")) OR Question "What are some of the reasons you stopped using PrEP to prevent HIV? Select all that apply." is exactly equal to ("I was worried about the safety of PrEP")) OR Question "What are some of the reasons you stopped using PrEP to prevent HIV? Select all that apply." is exactly equal to ("My kidney and liver function began to suffer")) OR Question "What are some of the reasons you stopped using PrEP to prevent HIV? Select all that apply." is exactly equal to ("I started a committed relationship")) OR Question "What are some of the reasons you stopped using PrEP to prevent HIV? Select all that apply." is exactly equal to ("I was using PrEP in a previous relationship with a person living with HIV")) OR Question "What are some of the reasons you stopped using PrEP to prevent HIV? Select all that apply." is exactly equal to ("I didn't think it was worth it")) OR Question "What are some of the reasons you stopped using PrEP to prevent HIV? Select all that apply." is exactly equal to ("I contracted HIV")) OR Question "What are some of the reasons you stopped using PrEP to prevent HIV? Select all that apply." is exactly equal to ("It was too inconvenient/didn't fit my life")) OR Question "What are some of the reasons you stopped using PrEP to prevent HIV? Select all that apply." is exactly equal to ("I was worried someone would think I had HIV if they saw me take it")) OR Question "What are some of the reasons you stopped using PrEP to prevent HIV? Select all that apply." is exactly equal to ("I decided I didn't need it anymore")) OR Question "What are some of the reasons you stopped using PrEP to prevent HIV? Select all that apply." is exactly equal to ("Other, please specify:")) **THEN:** Jump to page 39 - Future PrEP (FP1 - FP3)

Shortname / Alias: prep_never_main

ID: 427

Piping: Piped Values From Question (What are some of the reasons you've never used PrEP? Select all that apply.)

You said you've never used PrEP for the following reasons. Among these, which is the most important reason you've never used PrEP?

Page entry logic: This page will show when: Question "What are some of the reasons you stopped using PrEP to prevent HIV? Select all that apply." is one of the following answers ("I couldn't afford it","My insurance wouldn't cover it","My doctor decided PrEP was no longer appropriate for me","I didn't like the side effects","I was worried about the safety of PrEP","My kidney and liver function began to suffer","I started a committed relationship","I was using PrEP in a previous relationship with a person living with HIV","I didn't think it was worth it","I contracted HIV","It was too inconvenient/didn't fit my life","I was worried someone would think I had HIV if they saw me take it","I decided I didn't need it anymore","Other, please specify:")

PrEP Uptake / Adherence (PA9)

Page exit logic: Skip / Disqualify Logic**IF:** Question "Are you currently taking PrEP to prevent HIV?" is one of the following answers ("No") **THEN:** Jump to page 39 - Future PrEP (FP1 - FP3)

Shortname / Alias: prep_stop_main

ID: 426

Piping: Piped Values From Question (What are some of the reasons you stopped using PrEP to prevent HIV? Select all that apply.)

You said you stopped using PrEP for the following reasons. Among these, which is the most important reason you stopped using PrEP?

Page entry logic: This page will show when: (Question "What is your HIV status?" is one of the following answers ("HIV Negative","Indeterminate","I don't know") AND Question "Are you currently taking PrEP to prevent HIV?" is one of the following answers ("Yes"))

Currently taking PrEP (PA13 - PA16)

Shortname / Alias: prep_7d

ID: 112

In the past 7 days, on how many days did you take your dose of PrEP?

( ) 7
( ) 6
( ) 5
( ) 4
( ) 3
( ) 2
( ) 1
( ) 0

Shortname / Alias: prep_30d

ID: 113

In the past 30 days, on how many days did you take your dose of PrEP?

( ) 30
( ) 29
( ) 28
( ) 27
( ) 26
( ) 25
( ) 24
( ) 23
( ) 22
( ) 21
( ) 20
( ) 19
( ) 18
( ) 17
( ) 16
( ) 15
( ) 14

( ) 13

( ) 12

( ) 11

( ) 10

( ) 9

( ) 8

( ) 7

( ) 6

( ) 5

( ) 4

( ) 3

( ) 2

( ) 1

( ) 0

Shortname / Alias: prep_good

ID: 114

In the past 30 days, how good a job did you do at taking your PrEP medication in the way you were supposed to?

(1) Very poor

(2) Poor

(3) Fair

(4) Good

(5) Very good

(6) Excellent

Shortname / Alias: prep_often

ID: 115

In the past 30 days, how often did you take your PrEP medication in the way you were supposed to?

(1) Never

(2) Rarely

(3) Sometimes

(4) Usually

(5) Almost Always

(6) Always

Page entry logic: This page will show when: (Question "What is your HIV status?" is one of the following answers ("HIV Negative","Indeterminate","I don't know") AND Question "Are you currently taking PrEP to prevent HIV?" is one of the following answers ("Yes"))

Currently Taking PrEP (PA17 - PA22)

Shortname / Alias: prep_when

ID: 116

When do you take your PrEP medication?

(1) Before you have sex

(2) After you have sex

(3) Both before and after you have sex

(4) Other times, please specify:: ____

Shortname / Alias: prep_demand

ID: 117

Have you ever used PrEP "on-demand" or right before you knew you were about to have sex, as opposed to daily?

(1) Yes

(0) No

Logic: Show/hide trigger exists.

Shortname / Alias: prep_share

ID: 118

Have you ever shared your PrEP medication with others?

(1) Yes

(0) No

Logic: Hidden unless: Question "Have you ever shared your PrEP medication with others?" is one of the following answers ("Yes")

Shortname / Alias: prep_share_

ID: 119

Who did you share your PrEP medication with?

[1] Sex partner [PREP_SHARE_PARTNER]

[2] Friends [PREP_SHARE_FRIEND]

[3] Family [PREP_SHARE_FAMILY]

[4] Co-workers [PREP_SHARE_COWORKER]

[5] Other, please specify:: ____ [PREP_SHARE_OTHER] [PREP_SHARE_OTHERSP]

Page entry logic: This page will show when: Question "What is your HIV status?" is one of the following answers ("HIV Negative","Indeterminate","I don't know")

Future PrEP (FP1 - FP3)

Logic: Hidden unless: Question "Are you currently taking PrEP to prevent HIV?" is not one of the following answers ("Yes")

Shortname / Alias: prep_future

ID: 699

How likely would you be to use PrEP to reduce the risk of getting HIV?

(1) Very likely

(2) Somewhat likely

(3) Neither likely nor unlikely

(4) Somewhat unlikely

(5) Very unlikely

ID: 701

Researchers are also working on a form of PrEP that you would only take around the times you have sex. You would take two pills within 24 hours before sex and then two separate one-pill doses in the two days after sex.

Shortname / Alias: prep_pill

ID: 700

How likely would you be to use this type of PrEP that is taken only around the time you have sex?

(1) Very likely

(2) Somewhat likely

(3) Neither likely nor unlikely

(4) Somewhat unlikely

(5) Very unlikely

ID: 702

Researchers are working on a form of PrEP that a doctor would give as a shot into your arm. You would need to get the shot every 1 to 3 months to reduce your risk of getting HIV.

Shortname / Alias: prep_inject

ID: 704

How likely would you be to use this injectable form of PrEP to reduce the risk of getting HIV?

(1) Very likely

(2) Somewhat likely

(3) Neither likely nor unlikely

(4) Somewhat unlikely

(5) Very unlikely

Page entry logic: This page will show when: Question "What is your HIV status?" is one of the following answers ("HIV Negative","Indeterminate","I don't know")

Future PrEP (FP4 - FP7)

ID: 705

Researchers are also working on a form of PrEP that is a gel you would apply to the penis (like lubricant or lube) or put into the rectum with an applicator before and/or after sex to reduce the risk of getting HIV.

Shortname / Alias: prep_pa

ID: 831

Please select the best response to the questions below.

	Very likely (1)	Somewhat likely (2)	Neither likely nor	Somewhat unlikely (4)	Very unlikely (5)

			**unlikely (3)**		
How likely would you be to use a gel form of PrEP that you apply to your penis like lube **before** having insertive anal sex (topping) to reduce the risk of getting HIV? [PREP_PGEL_BF]	( )	( )	( )	( )	( )
How likely would you be to use a gel form of PrEP that you apply to your penis like lube **after** having insertive anal sex (topping) to reduce the risk of getting HIV? [PREP_PGEL_AF]	( )	( )	( )	( )	( )
How likely would you be to use a gel form of PrEP that you squeeze into your rectum using an applicator **before** receptive sex (bottoming) to reduce the risk of getting HIV? [PREP_AGEL_BF]	( )	( )	( )	( )	( )
How likely would you be to use a gel form of PrEP that you squeeze into your rectum using	( )	( )	( )	( )	( )

an applicator **after** receptive sex (bottoming) to reduce the risk of getting HIV? [PREP_AGEL_AF]					

Page entry logic: This page will show when: Question "What is your HIV status?" is one of the following answers ("HIV Negative","Indeterminate","I don't know")

Future PrEP (FP8 - FP9)

ID: 711

Researchers are also working on a form of PrEP that you would put in your rectum as a suppository. This would involve putting a pill the size of a daily vitamin pill into your rectum at least 30 minutes before having receptive anal sex (bottoming) to be sure it dissolved in time to reduce the risk of getting HIV.

Shortname / Alias: prep_supp_bf

ID: 712

How likely would you be to use a suppository form of PrEP that you insert into your rectum to dissolve 30 minutes *before* having receptive anal sex (bottoming) to reduce the risk of getting HIV?

(1) Very likely

(2) Somewhat likely

(3) Neither likely nor unlikely

(4) Somewhat unlikely

(5) Very unlikely

Shortname / Alias: prep_supp_af

ID: 713

How likely would you be to use a suppository form of PrEP that you insert into your rectum to dissolve within three hours *after* having receptive anal sex (bottoming) to reduce the risk of getting HIV?

(1) Very likely

(2) Somewhat likely

(3) Neither likely nor unlikely

(4) Somewhat unlikely

(5) Very unlikely

Page entry logic: This page will show when: Question "What is your HIV status?" is one of the following answers ("HIV Negative","Indeterminate","I don't know")

Future PrEP (FP10)

Shortname / Alias: preprank_

ID: 714

Below is a list that includes the currently available form of PrEP and condoms, and all the different forms of PrEP that researchers are working on. Please rank in order of the methods you would use, from most preferred (1) to least preferred (8)? Drag the items to the next column, starting with your first choice of a prevention method.

________Daily PrEP (a pill that you take every day) [PREPRANK_PILL]

________Event-level PrEP (two pills before sex and one pill for two days after sex) [PREPRANK_EVENT]

________Injectable PrEP (a shot you receive every 1-3 months) [PREPRANK_INJECT]

________Rectal suppository PrEP that is used before sex (a pill-size dose you put in your rectum) [PREPRANK_SUPPBF]

________Rectal suppository PrEP that is used after sex (a pill-size dose you put in your rectum) [PREPRANK_SUPPAF]

________Rectal gel PrEP before sex (a gel that you squeeze into your rectum with an applicator) [PREPRANK_GELBF]

________Rectal gel PrEP after sex (a gel that you squeeze into your rectum with an applicator) [PREPRANK_GELAF]

________Condoms [PREPRANK_CONDOM]

Future PrEP (FP11)

Shortname / Alias: prevent_

ID: 715

Listed below are general qualities of prevention methods. Please rank in order of importance to you, from most important (1) to least important (7)? Drag the items to the next column, starting with the most important prevention method quality.

________Effective against HIV [PREVENT_HIV]

________Effective against other STDs [PREVENT_STD]

________Doesn't reduce physical pleasure [PREVENT_PLEASURE]

________Doesn't break the mood of sex [PREVENT_MOOD]

________You control it, not your partner [PREVENT_YOU]

________Your partner controls it, not you [PREVENT_PARTNER]

________Recommended by experts [PREVENT_RECOMMEND]

Future PrEP (FP12)

Shortname / Alias: condomorpill

ID: 716

If you had a choice of using condoms during anal sex or taking a pill every day to prevent HIV infection, both at no cost, which would you choose?

(1) Condoms only

(2) Daily pill only

(3) Both condoms and daily pill

Page entry logic: This page will show when: Question "What is your HIV status?" is one of the following answers ("HIV Negative","Indeterminate","I don't know")

PrEP Care Engagement (PC1 - PC2)

Logic: Hidden unless: Question "Are you currently taking PrEP to prevent HIV?" is one of the following answers ("Yes")

Shortname / Alias: prepcare

ID: 120

In the past 3 months, that is since [question('value'), id='760'], have you seen a doctor, nurse or other health care provider to receive tests or other care related to your PrEP use?

(1) Yes

(0) No

(7) I don't know

Logic: Hidden unless: Question "Are you currently taking PrEP to prevent HIV?" is one of the following answers ("No")

Shortname / Alias: prep_use

ID: 121

In the past 3 months, that is since [question('value'), id='760'], have you seen a doctor, nurse or other health care provider about using PrEP?

(1) Yes

(0) No

General Care Engagement (GC1 - GC2)

Logic: Hidden unless: ((Question "What is your HIV status?" is one of the following answers ("HIV Negative","Indeterminate","I don't know") AND Question "Are you currently taking PrEP to prevent HIV?" is one of the following answers ("No")) AND Question "In the past 3 months, that is since [question('value'), id='760'], have you seen a doctor, nurse or other health care provider about using PrEP?" is one of the following answers ("No"))

Shortname / Alias: medcare

ID: 123

In the past 3 months, that is since [question('value'), id='760'], have you seen a doctor, nurse or other health care provider for any other reason?

(1) Yes

(0) No

Logic: Hidden unless: ((Question "What is your HIV status?" is one of the following answers ("HIV Positive") AND Question "Are you currently prescribed and taking any antiretroviral medicines to treat your HIV?" is one of the following answers ("No")) OR (Question "What is your HIV status?" is one of the following answers ("HIV Negative","Indeterminate","I don't know") AND Question "Are you currently taking PrEP to prevent HIV?" is one of the following answers ("No")))

Shortname / Alias: med_

ID: 124

Think about times when you have been prescribed medicine by a doctor because you were sick or for a chronic condition (e.g., diabetes, high blood pressure).

	**Yes (1)**	**No (0)**

Did you ever forget to take your medicine? [MED_FORGET]	( )	( )
Did you ever have problems taking your medicine? [MED_PROBLEMS]	( )	( )
When you felt better, did you sometimes stop taking your medicine before you should? [MED_BETTERSTOP]	( )	( )
Sometimes, if you felt worse when taking your medicine, did you stop taking it? [MED_WORSESTOP]	( )	( )

Efficacy/Intentions (EI1)

Validation: Min = 0 Max = 100

Logic: Hidden unless: Question "What is your HIV status?" is one of the following answers ("HIV Negative","Indeterminate","I don't know")

Shortname / Alias: protect_

ID: 129

On a scale of 0 to 100, where 0 equals no protection and 100 equals complete protection, how much protection do you think each of the following strategies offers in preventing HIV?

Please slide the yellow dot on the scale to indicate how much protection you think each strategy offers.

Taking PrEP [PROTECT_PREP]	0 ______________________[__]________________________ ___ 100
Using condoms [PROTECT_CONDOM]	0 ______________________[__]________________________ ___ 100
An HIV-positive partner taking HIV medications [PROTECT_HIVMEDS]	0 ______________________[__]________________________ ___ 100
Getting tested for HIV [PROTECT_HIVTEST]	0 ______________________[__]________________________ ___ 100
Getting tested for STDs [PROTECT_STDTEST]	0 ______________________[__]________________________ ___ 100
Always using condoms with HIV-negative partners [PROTECT_HIVPOSCON D]	0 ______________________[__]________________________ ___ 100
Always using condoms with HIV-positive partners [PROTECT_HIVNEGCON D]	0 ______________________[__]________________________ ___ 100

Efficacy/Intentions (EI2)

Validation: Min = 0 Max = 100

Logic: Hidden unless: Question "What is your HIV status?" is one of the following answers ("HIV Positive")

Shortname / Alias: protectpos_

ID: 556

On a scale of 0 to 100, where 0 equals no protection and 100 equals complete protection, how much protection do you think each of the following strategies offers in preventing HIV?

Please slide the blue dot on the scale to indicate how much protection you think each strategy

offers.

Your partner taking PrEP [PROTECTPOS_PREP]	0 _________________________[__]_______________________________ 100
Using condoms [PROTECTPOS_CONDOM]	0 _________________________[__]_______________________________ 100
Taking medications to treat your HIV [PROTECTPOS_HIVMED]	0 _________________________[__]_______________________________ 100
Your partner getting tested for HIV [PROTECTPOS_HIVTEST]	0 _________________________[__]_______________________________ 100
Getting tested for STDs [PROTECTPOS_STDTEST]	0 _________________________[__]_______________________________ 100
Always using condoms with HIV-negative partners [PROTECTPOS_HIVNEGCOND]	0 _________________________[__]_______________________________ 100

Efficacy/Intentions (EI3)

Shortname / Alias: Likely_

ID: 130

In the next 3 months, how likely are you to do each of the following?

	**Definitely not likely (1)**	**Probably not likely (2)**	**Somewhat likely (3)**	**Probably likely (4)**	**Definitely likely (5)**	**Doesn't apply to me (77)**

Use condoms every time you have anal sex [LIKELYCONDOM]	( )	( )	( )	( )	( )	( )
Get tested for HIV [LIKELYHIVTEST]	( )	( )	( )	( )	( )	( )
Get tested for STDs [LIKELYSTDTEST]	( )	( )	( )	( )	( )	( )
Start taking antiretroviral medications to treat your HIV [LIKELYARTSTRT]	( )	( )	( )	( )	( )	( )
Take all of your HIV medications exactly as you are supposed to [LIKELYARTCONT]	( )	( )	( )	( )	( )	( )
Start taking PrEP [LIKELYPREPSTRT]	( )	( )	( )	( )	( )	( )
Take all of your PrEP doses exactly as you are supposed to [LIKELYPREPCONT]	( )	( )	( )	( )	( )	( )
See a doctor, nurse or other provider for HIV care	( )	( )	( )	( )	( )	( )

[LIKELYHIVCARE]						
See a doctor, nurse or other health care provider [LIKELYHLTHCARE]	( )	( )	( )	( )	( )	( )
Get a viral load test [LIKELYVIRAL]	( )	( )	( )	( )	( )	( )

Sex Behaviors (SX1 - SX2)

Shortname / Alias: mpartner

ID: 243

Have you had anal sex with a male primary partner in the past 3 months?
By “primary” partner we mean a man you have lived with or have seen a lot, to whom you have felt a special emotional commitment for at least 3 months. This includes times when you were a top or bottom, used or didn't use a condom, and whether or not there was ejaculation.

(1) Yes

(0) No

Validation: Must be numeric Whole numbers only Positive numbers only

Shortname / Alias: opartner_num

ID: 504

Now we want to ask you about all of the other men you have had any anal sex with in the past 3 months other than your most recent primary partner.

In the past 3 months, with how many men <u>other than your most recent primary partner</u> did you have <u>anal sex</u>?
By non-primary partner, we mean someone you had sex with but do not feel committed to or don't know very well. This includes times when you were a top or bottom, used or didn't use a condom, and whether or not there was ejaculation.

____

Page entry logic: This page will show when: (Question "Have you had anal sex with a male <u>primary</u> partner in the **past 3 months**?
By "primary" partner we mean a man you have lived with or have

seen a lot, to whom you have felt a special emotional commitment for at least 3 months. This includes times when you were a top or bottom, used or didn't use a condom, and whether or not there was ejaculation.
" is one of the following answers ("No") AND (Question "Now we want to ask you about all of the other men you have had any anal sex with in the **past 3 months** other than your most recent primary partner.

In the **past 3 months**, with how many men <u>other than your most recent primary partner</u> did you have <u>anal sex</u>?
By non-primary partner, we mean someone you had sex with but do not feel committed to or don't know very well. This includes times when you were a top or bottom, used or didn't use a condom, and whether or not there was ejaculation." is exactly equal to "0" OR Question "Now we want to ask you about all of the other men you have had any anal sex with in the **past 3 months** other than your most recent primary partner.

In the **past 3 months**, with how many men <u>other than your most recent primary partner</u> did you have <u>anal sex</u>?
By non-primary partner, we mean someone you had sex with but do not feel committed to or don't know very well. This includes times when you were a top or bottom, used or didn't use a condom, and whether or not there was ejaculation."))

Sex Behaviors (SX3)

Page exit logic: Page Logic**IF:** Question "You indicated that you did not have any sex partners in the **past 3 months**, is this correct?" is one of the following answers ("Yes") **THEN:** Jump to page 103 - Substance Use and Substance Dependency (SU1)

Page exit logic: Page Logic**IF:** Question "You indicated that you did not have any sex partners in the **past 3 months**, is this correct?" is one of the following answers ("No, I want to change my answer") **THEN:** Jump to page 50 - Sex Behaviors (SX1 - SX2)

Shortname / Alias: nosex

ID: 236

You indicated that you did not have any sex partners in the past 3 months, is this correct?

(1) Yes

(0) No, I want to change my answer

(untitled)

Action: Custom Script: part_ver setup

Hidden Value: sex4/casual partner number

Value:

Hidden Value: Total partners

Value:

Hidden Value: main partner one/none

Value:

Sex Behavior (SX4)

Page exit logic: Skip / Disqualify Logic**IF:** Question "Thanks. So you indicated that in the **past 3 months** you had a total of **[question('value'), id='588']** male sex partners, including **[question('value'), id='586']** primary partner and **[question('value'), id='510']** non-primary partners. Is that correct?" is one of the following answers ("No, I want to change my answer") **THEN:** Jump to page 50 - Sex Behaviors (SX1 - SX2)

Shortname / Alias: partner_total

ID: 585

Thanks. So you indicated that in the past 3 months you had a total of [question('value'), id='588'] male sex partners, including [question('value'), id='586'] primary partner and [question('value'), id='510'] non-primary partners. Is that correct?

(1) Yes

(0) No, I want to change my answer

Page entry logic: This page will show when: Question "Have you had anal sex with a male primary partner in the **past 3 months**?
By "primary" partner we mean a man you have lived with or have seen a lot, to whom you have felt a special emotional commitment for at least 3 months. This includes times when you were a top or bottom, used or didn't use a condom, and whether or not there was ejaculation.
" is one of the following answers ("Yes")

Primary Partner (SXP1)

Validation: Min character count = 1

Shortname / Alias: mp_nicknm

ID: 245

Throughout this next group of questions, we will ask you about your recent male primary partner using a nickname you have provided to make the questions specific to your partner. Examples of a nickname that you might choose are: a partner's first name, a nickname you call the partner by, the place where you both met, or an online screen name.

Please choose a nickname that will best help you know to whom we are referring. The nickname is to make the survey easier to take and you should not reveal your partner's full identity. We do not wish to know who your partner actually is.

Enter the nickname for your recent male primary partner below. *

____

Page entry logic: This page will show when: Question "Have you had anal sex with a male primary partner in the **past 3 months**?
By "primary" partner we mean a man you have lived with or have seen a lot, to whom you have felt a special emotional commitment for at least 3 months. This includes times when you were a top or bottom, used or didn't use a condom, and whether or not there was ejaculation.
" is one of the following answers ("Yes")

Primary Partner (SXP2 - SXP6)

Validation: Min = 10 Max = 100 Must be numeric Whole numbers only Positive numbers only

Shortname / Alias: mp_age

ID: 500

How old is [question('value'), id='245',case='upper']? If you are not sure, give it your best guess.

____

Shortname / Alias: mp_race

ID: 248

What is [question('value'), id='245',case='upper']'s primary race/ethnicity? If you are not sure, give it your best guess.

(1) African American or Black

(2) Asian American or Pacific Islander

(3) Latino, Hispanic, or Chicano

(4) Native American, American Indian, or Alaska Native

(5) White or European American

(6) Mixed

(7) Other, please specify:: ____

Shortname / Alias: mp_met

ID: 249

Where did you first meet [question('value'), id='245',case='upper']? (Choose one)

(1) A dating or hookup app/website for gay men, like Grindr or Jack'd

(2) A social networking app or website for gay men (not for dating or hooking up)

(3) A social media app or website, like Facebook, Twitter, Instagram or Snapchat

(4) Some other app or website (such as tinder, okcupid, eharmony, etc.)

(5) Work or school

(6) Through family

(7) Through a friend

(8) A private party or social event

(9) A public festival or community event, like Pride

(10) A gay commercial venue, like a gay bar or dance club

(11) A straight commercial venue, like a straight bar or dance club

(12) A commercial sex venue, like a sex club or bath house

(13) A public sex venue, like a park, beach, bathroom or tearoom

(14) Other, please specify:: ____

Page entry logic: This page will show when: Question "Have you had anal sex with a male primary partner in the **past 3 months**?
By "primary" partner we mean a man you have lived with or have seen a lot, to whom you have felt a special emotional commitment for at least 3 months. This includes times when you were a top or bottom, used or didn't use a condom, and whether or not there was ejaculation.
" is one of the following answers ("Yes")

Primary Partner (SXP7 - SXP10)

Page exit logic: Skip / Disqualify Logic**IF:** Question "What is **[question('value'), id='245',case='upper']'s** HIV status?" is one of the following answers ("Negative") **THEN:** Jump to page 58 - Primary Partner (SXP15 - SXP17)

Shortname / Alias: mp_1sex

ID: 250

When was the first time you had any anal sex with [question('value'), id='245',case='upper']? That is, where you were a top or bottom, with or without a condom, and with or without ejaculation.

(1) Less than a week ago

(2) More than a week but less than a month ago

(3) One to three months ago

(4) Four to six months ago

(5) Seven to twelve months ago

(6) More than a year ago

Logic: Show/hide trigger exists.

Shortname / Alias: mp_status

ID: 251

What is [question('value'), id='245',case='upper']'s HIV status?

(1) Positive

(2) Negative

(7) I don't know

Shortname / Alias: mp_ptell

ID: 252

Did [question('value'), id='245',case='upper'] TELL you his HIV-status?

(1) Yes

(0) No

Shortname / Alias: mp_ytold

ID: 253

Have you told [question('value'), id='245',case='upper'] your current HIV status?

(1) Yes

(0) No

Page entry logic: This page will show when: (Question "Have you had anal sex with a male primary partner in the **past 3 months**?
By "primary" partner we mean a man you have lived with or have seen a lot, to whom you have felt a special emotional commitment for at least 3 months. This includes times when you were a top or bottom, used or didn't use a condom, and whether or not there was ejaculation.
" is one of the following answers ("Yes") AND Question "What is **[question('value'), id='245',case='upper']'s** HIV status?" is one of the following answers ("Positive"))

Primary Partner (SXP11 - SXP14)

Page exit logic: Skip / Disqualify Logic**IF:** Question "What is **[question('value'), id='245',case='upper']'s** HIV status?" is one of the following answers ("Positive") **THEN:** Jump to page 59 - Primary Partner (SXP18 - SXP21)

Logic: Show/hide trigger exists.

Shortname / Alias: mp_art

ID: 254

Have you ever talked to [question('value'), id='245',case='upper'] about whether he is taking antiretroviral medicines to treat his HIV?

(1) Yes

(0) No

Logic: Hidden unless: Question "Have you ever talked to [question('value'), id='245',case='upper'] about whether he is taking antiretroviral medicines to treat his HIV?" is one of the following answers ("Yes")

Shortname / Alias: mp_art_3m

ID: 255

In the past 3 months, that is since [question('value'),id='760'], have you talked to [question('value'), id='245',case='upper'] about whether he is taking antiretroviral medicines to treat his HIV?

(1) Yes

(0) No

Logic: Show/hide trigger exists. Hidden unless: Question "Have you ever talked to [question('value'), id='245',case='upper'] about whether he is taking antiretroviral medicines to treat his HIV?" is one of the following answers ("Yes")

Shortname / Alias: mp_art_current

ID: 257

Has [question('value'), id='245',case='upper'] told you he was currently taking antiretroviral medicines to treat his HIV?

(1) Yes, and I have no reason to doubt him

(2) Yes, but I have reason to doubt him

(0) No

Logic: Hidden unless: Question "Has [question('value'), id='245',case='upper'] told you he was currently taking antiretroviral medicines to treat his HIV?" is one of the following answers ("Yes, and I have no reason to doubt him","Yes, but I have reason to doubt him")

Shortname / Alias: mp_undetect

ID: 258

Has [question('value'), id='245',case='upper'] told you his viral load is undetectable?

(1) Yes, and I have no reason to doubt him

(2) Yes, but I have some reason to doubt him

(0) No

Commented [MS1]: This differs from the previous question by one word. Can we make these consistent?

Page entry logic: This page will show when: (Question "Have you had anal sex with a male primary partner in the **past 3 months**?
By "primary" partner we mean a man you have lived with or have seen a lot, to whom you have felt a special emotional commitment for at least 3 months. This includes times when you were a top or bottom, used or didn't use a condom, and whether or not there was ejaculation.
" is one of the following answers ("Yes") AND Question "What is **[question('value'), id='245',case='upper']'s** HIV status?" is one of the following answers ("Negative"))

Primary Partner (SXP15 - SXP17)

Logic: Show/hide trigger exists.

Shortname / Alias: mp_prep

ID: 259

Have you ever talked to [question('value'), id='245',case='upper'] about whether he is taking PrEP to prevent HIV?

(1) Yes

(0) No

Logic: Hidden unless: Question "Have you ever talked to [question('value'), id='245',case='upper'] about whether he is taking PrEP to prevent HIV?" is one of the following answers ("Yes")

Shortname / Alias: mp_prep_3m

ID: 260

In the past 3 months, that is since [question('value'),id='760'], have you talked to [question('value'), id='245',case='upper'] about whether he is taking PrEP to prevent HIV?

(1) Yes

(0) No

Logic: Hidden unless: Question "Have you ever talked to [question('value'), id='245',case='upper'] about whether he is taking PrEP to prevent HIV?" is one of the following answers ("Yes")

Shortname / Alias: mp_prep_current

ID: 261

Has [question('value'), id='245',case='upper'] told you he is currently taking PrEP to prevent HIV?

(1) Yes, and I have no reason to doubt him

(2) Yes, but I have reason to doubt him

(0) No

Page entry logic: This page will show when: Question "Have you had anal sex with a male primary partner in the **past 3 months**?
By "primary" partner we mean a man you have lived with or have seen a lot, to whom you have felt a special emotional commitment for at least 3 months. This includes times when you were a top or bottom, used or didn't use a condom, and whether or not there was ejaculation.
" is one of the following answers ("Yes")

Primary Partner (SXP18 - SXP21)

**Logic: Show/hide trigger exists.**
Shortname / Alias: mp_uai
ID: 262

In the past 3 months, that is since [question('value'),id='760'], have you had ANY anal sex with [question('value'), id='245',case='upper'] in which a condom was <u>not</u> used from start to finish?

(1) Yes

(0) No

(7) I don't know

**Logic: Hidden unless: Question "In the past 3 months, that is since [question('value'),id='760'], have you had ANY anal sex with [question('value'), id='245',case='upper'] in which a condom was <u>not</u> used from start to finish?" is one of the following answers ("Yes")**
Shortname / Alias: mp_uai_position
ID: 263

Think of the times in the past 3 months that you had anal sex with [question('value'), id='245',case='upper'] and did <u>not</u> use a condom from start to finish. Were you the top (you put your penis in his butt), the bottom (he put his penis in your butt), or both?

(1) I was always the top

(2) I was always the bottom

(3) I was both the top and the bottom

**Logic: Hidden unless: Question "In the past 3 months, that is since [question('value'),id='760'], have you had ANY anal sex with [question('value'), id='245',case='upper'] in which a condom was <u>not</u> used from start to finish?" is one of the following answers ("Yes")**
Shortname / Alias: mp_uai_drunk

ID: 264

Think of the times in the past 3 months that you had anal sex with [question('value'), id='245',case='upper'] and did not use a condom from start to finish. Were you ever drunk or buzzed on alcohol within 2 hours before or during sex?

(1) Yes

(0) No

Logic: Hidden unless: Question "In the past 3 months, that is since [question('value'),id='760'], have you had ANY anal sex with [question('value'), id='245',case='upper'] in which a condom was not used from start to finish?" is one of the following answers ("Yes")

Shortname / Alias: mp_uai_drugs

ID: 265

Think of the times in the past 3 months that you had anal sex with [question('value'), id='245',case='upper'] and did not use a condom from start to finish. Did you ever use non-prescription drugs within 2 hours before or during sex?

(1) Yes

(0) No

Page entry logic: This page will show when: Question "Have you had anal sex with a male primary partner in the **past 3 months**?
By "primary" partner we mean a man you have lived with or have seen a lot, to whom you have felt a special emotional commitment for at least 3 months. This includes times when you were a top or bottom, used or didn't use a condom, and whether or not there was ejaculation.
" is one of the following answers ("Yes")

Last Sex with Male Primary Partner (SXP22 - SXP23)

ID: 266

The following questions ask about the last time you had sex with your primary partner, [question('value'), id='245',case='upper'].

Shortname / Alias: mp_last_ai

ID: 267

How long ago was the last time you had any anal sex with [question('value'), id='245',case='upper']? That is, where you were a top or a bottom, with or without a condom, and with or without ejaculation.

(1) 1 day

(2) 2 days

(3) 3 days

(4) 4 days

(5) 5 days

(6) 6 days

(7) 1 week

(8) 2 weeks

(9) 3 weeks

(10) 1 month

(11) 2 months

(12) 3 months

(13) More than 3 months

Shortname / Alias: mp_last_where

ID: 268

During the last time you had anal sex with [question('value'), id='245',case='upper'], where did you have sex?

(1) Your house or apartment or his house or apartment

(2) Someone else's house or apartment

(3) Hotel

(4) Sex club or bathhouse

(5) Bar, night club, or dance club

(6) Health club or gym

(7) Porn theatre/video arcade

(8) Public bathroom

(9) Other public place, such as beach

(10) Park, woods, street, car or van

(11) Other, please specify:: ____

Page entry logic: This page will show when: Question "Have you had anal sex with a male primary partner in the **past 3 months**?
By "primary" partner we mean a man you have lived with or have seen a lot, to whom you have felt a special emotional commitment for at least 3 months. This includes times when you were a top or bottom, used or didn't use a condom, and whether or not there was ejaculation.
" is one of the following answers ("Yes")

Last Sex with Male Primary Partner (SXP24 - SXP27)

Page exit logic: Skip / Disqualify Logic**IF:** Question "Are you currently taking PrEP to prevent HIV?" is one of the following answers ("No") **THEN:** Jump to page 63 - Last Sex with Male Primary Partner - Partner PrEP (SXP31 - SXP33)

Logic: Show/hide trigger exists.

Shortname / Alias: mp_top

ID: 271

During the most recent time you had anal sex with [question('value'), id='245',case='upper'], were you the top (you put your penis in his butt)? This would be with or without a condom and with or without ejaculation.

(1) Yes

(0) No

Logic: Hidden unless: Question "During the most recent time you had anal sex with [question('value'), id='245',case='upper'], were you the top (you put your penis in his butt)? This would be with or without a condom and with or without ejaculation." is one of the following answers ("Yes")

Shortname / Alias: mp_top_condom

ID: 272

Was a condom used from start to finish when you were the top?

(1) Yes

(0) No

(7) I don't know

Logic: Show/hide trigger exists.

Shortname / Alias: mp_bottom

ID: 273

During the most recent time you had anal sex with [question('value'), id='245',case='upper'], were you the bottom (he put his penis in your butt)? This would be with or without a condom and whether or not you ejaculated.

(1) Yes

(0) No

Logic: Hidden unless: Question "During the most recent time you had anal sex with [question('value'), id='245',case='upper'], were you the bottom (he put his penis in your butt)? This would be with or without a condom and whether or not you ejaculated." is one of the following answers ("Yes")

Shortname / Alias: mp_bottom_condom

ID: 274

Was a condom used from start to finish when you were the bottom?

(1) Yes

(0) No

(7) I don't know

Page entry logic: This page will show when: ((Question "Have you had anal sex with a male primary partner in the **past 3 months**?
By "primary" partner we mean a man you have lived with or have seen a lot, to whom you have felt a special emotional commitment for at least 3 months. This includes times when you were a top or bottom, used or didn't use a condom, and whether or not there was ejaculation.
" is one of the following answers ("Yes") AND Question "What is your HIV status?" is one of the following answers ("HIV Negative","Indeterminate","I don't know")) AND Question "Have you ever used PrEP to prevent HIV?" is one of the following answers ("Yes"))

Last Sex with Male Primary Partner (SXP28 - SXP30)

Logic: Show/hide trigger exists.

Shortname / Alias: mp_yprep24

ID: 275

Were you taking PrEP medication to prevent HIV within 24 hours before or after the time you last had anal sex with [question('value'), id='245',case='upper']?

(1) Yes

(0) No

(7) I don't know

Logic: Show/hide trigger exists. Hidden unless: Question "Were you taking PrEP medication to prevent HIV within 24 hours before or after the time you last had anal sex with [question('value'), id='245',case='upper']?" is one of the following answers ("Yes")

Shortname / Alias: mp_yprep7

ID: 276

Were you taking your PrEP medication once a day as prescribed by your doctor, without missing doses, in the 7 days before the time you last had anal sex with [question('value'), id='245',case='upper']?

(1) Yes

(0) No

(7) I don’t know

Logic: Hidden unless: Question "Were you taking your PrEP medication once a day as prescribed by your doctor, without missing doses, in the 7 days before the time you last had anal sex with [question('value'), id='245',case='upper']?" is one of the following answers ("No")

Shortname / Alias: mp_yprepmiss

ID: 435

You mentioned that you were not taking your PrEP medication once a day as prescribed at the time of your last sexual encounter. In the 7 days before you last had anal sex with [question('value'), id='245',case='upper'], on how many days did you take your dose of PrEP?

(7) 7

(6) 6

(5) 5

(4) 4

(3) 3

(2) 2

(1) 1

(0) 0

(77) I don't know

(99) I'd prefer not to answer

Page entry logic: This page will show when: (Question "Have you had anal sex with a male primary partner in the **past 3 months**?
By “primary” partner we mean a man you have lived with or have seen a lot, to whom you have felt a special emotional commitment for at least 3 months. This includes times when you were a top or bottom, used or didn't use a condom, and whether or not there was ejaculation.
" is one of the following answers ("Yes") AND Question "What is **[question('value'), id='245',case='upper']'s** HIV status?" is one of the following answers ("Negative"))

Last Sex with Male Primary Partner - Partner PrEP (SXP31 - SXP33)

Page exit logic: Skip / Disqualify Logic**IF:** Question "What is your HIV status?" is not one of the following answers ("HIV Positive") **THEN:** Jump to page 65 - Last Sex with Male Primary Partner - Partner ART (SXP37 - SXP41)

Logic: Show/hide trigger exists.

Shortname / Alias: mp_pprep24

ID: 277

Was [question('value'), id='245',case='upper'] taking PrEP medication to prevent HIV within 24 hours before or after the time you last had anal sex?

(1) Yes

(0) No

(7) I don't know

Logic: Show/hide trigger exists. Hidden unless: Question "Was [question('value'), id='245',case='upper'] taking PrEP medication to prevent HIV within 24 hours before or after the time you last had anal sex?
" is one of the following answers ("Yes")

Shortname / Alias: mp_pprep7

ID: 278

To your knowledge, was [question('value'), id='245',case='upper'] taking his PrEP medication once a day as prescribed by his doctor, without missing doses, in the 7 days before you last had anal sex?

(1) Yes

(0) No

(7) I don’t know

Logic: Hidden unless: Question "To your knowledge, was [question('value'), id='245',case='upper'] taking his PrEP medication once a day as prescribed by his doctor, without missing doses, in the 7 days before you last had anal sex?" is one of the following answers ("No")

Shortname / Alias: mp_pprepmiss

ID: 436

You mentioned that [question('value'), id='245',case='upper'] was not taking his PrEP medication once a day as prescribed at the time of your last sexual encounter. To your knowledge, in the 7 days before you had anal sex, on how many days did [question('value'), id='245',case='upper'] take his dose of PrEP?

(7) 7

(6) 6

(5) 5

(4) 4

(3) 3

(2) 2

(1) 1

(0) 0

(77) I don't know

(99) I'd prefer not to answer

Page entry logic: This page will show when: ((Question "Have you had anal sex with a male primary partner in the **past 3 months**?
By "primary" partner we mean a man you have lived with or have seen a lot, to whom you have felt a special emotional commitment for at least 3 months. This includes times when you were a top or bottom, used or didn't use a condom, and whether or not there was ejaculation.
" is one of the following answers ("Yes") AND Question "What is your HIV status?" is one of the following answers ("HIV Positive")) AND Question "Have you ever been prescribed and taken antiretroviral medications to treat your HIV?" is one of the following answers ("Yes"))

Last Sex with Primary Partner - Recent ART (SXP34 - SXP36)

Logic: Show/hide trigger exists.

Shortname / Alias: mp_yart24

ID: 437

Were you taking antiretroviral medicines to treat your HIV infection within 24 hours before or after the time you last had anal sex with [question('value'), id='245',case='upper']?

(1) Yes

(0) No

(7) I don't know

Logic: Show/hide trigger exists. Hidden unless: Question "Were you taking antiretroviral medicines to treat your HIV infection within 24 hours before or after the time you last had anal sex with [question('value'), id='245',case='upper']?" is one of the following answers ("Yes")

Shortname / Alias: mp_yart7

ID: 438

Were you taking your HIV medicines exactly as prescribed by your doctor, without missing any doses, in the 7 days before you last had anal sex with [question('value'), id='245',case='upper']?

(1) Yes

(0) No

(7) I don't know

Logic: Hidden unless: Question "Were you taking your HIV medicines exactly as prescribed by your doctor, without missing any doses, in the 7 days before you last had anal sex with [question('value'), id='245',case='upper']?" is one of the following answers ("No")

Shortname / Alias: mp_yartmiss

ID: 439

You mentioned that you were not taking your HIV medicines exactly as prescribed at the time of your last sexual encounter. In the 7 days before you last had anal sex

with [question('value'), id='245',case='upper'], on how many days did you take all of the doses of your HIV medicines?

(7) 7

(6) 6

(5) 5

(4) 4

(3) 3

(2) 2

(1) 1

(0) 0

(77) I don't know

(99) I'd prefer not to answer

Page entry logic: This page will show when: (Question "Have you had anal sex with a male primary partner in the **past 3 months**?
By “primary” partner we mean a man you have lived with or have seen a lot, to whom you have felt a special emotional commitment for at least 3 months. This includes times when you were a top or bottom, used or didn't use a condom, and whether or not there was ejaculation.
" is one of the following answers ("Yes") AND Question "What is **[question('value'), id='245',case='upper']'s** HIV status?" is one of the following answers ("Positive"))

Last Sex with Male Primary Partner - Partner ART (SXP37 - SXP41)

Logic: Show/hide trigger exists. Hidden unless: Question "What is [question('value'), id='245',case='upper']'s HIV status?" is one of the following answers ("Positive")

Shortname / Alias: mp_part24

ID: 279

Was [question('value'), id='245',case='upper'] taking antiretroviral medicines to treat his HIV infection within 24 hours before or after the time you last had anal sex?

(1) Yes

(0) No

(7) I don't know

Logic: Hidden unless: (Question "Was [question('value'), id='245',case='upper'] taking antiretroviral medicines to treat his HIV infection within 24 hours before or after the time you last had anal sex?" is one of the following answers ("Yes") AND Question "What is [question('value'), id='245',case='upper']'s HIV status?" is one of the following answers ("Positive"))

Shortname / Alias: mp_part7

ID: 280

To your knowledge, was [question('value'), id='245',case='upper'] taking his medicines exactly as prescribed by his doctor, without missing any doses, in the 7 days before you last had anal sex?

(1) Yes

(0) No

(7) I don’t know

Logic: Hidden unless: (Question "To your knowledge, was [question('value'), id='245',case='upper'] taking his medicines exactly as prescribed by his doctor, without missing any doses, in the 7 days before you last had anal sex?" is one of the following answers ("No") AND Question "What is [question('value'), id='245',case='upper']'s HIV status?" is one of the following answers ("Positive"))

Shortname / Alias: mp_partmiss

ID: 440

You mentioned that [question('value'), id='245',case='upper'] was not taking his HIV medicines exactly as prescribed at the time of your last sexual encounter. To your knowledge, in the 7 days before you last had anal sex, on how many days did [question('value'), id='245',case='upper'] take all of the doses of his HIV medicines?

(7) 7

(6) 6

(5) 5

(4) 4

(3) 3

(2) 2

(1) 1

(0) 0

(77) I don't know

(99) I'd prefer not to answer

Logic: Show/hide trigger exists. Hidden unless: Question "Was [question('value'), id='245',case='upper'] taking antiretroviral medicines to treat his HIV infection within 24 hours before or after the time you last had anal sex?" is one of the following answers ("Yes")

Shortname / Alias: mp_ai_vl

ID: 281

Did [question('value'), id='245',case='upper'] tell you his viral load was undetectable before you last had anal sex?

(1) Yes

(0) No

Logic: Hidden unless: Question "Did [question('value'), id='245',case='upper'] tell you his viral load was undetectable before you last had anal sex?" is one of the following answers ("Yes")

Shortname / Alias: mp_aivl_when

ID: 441

When was the last time [question('value'), id='245',case='upper'] told you his viral load was undetectable?

(1) Less than a week ago

(2) More than a week but less than a month ago

(3) One to three months ago

(4) Four to six months ago

(5) Seven to twelve months ago

(6) More than a year ago

Page entry logic: This page will show when: Question "Have you had anal sex with a male primary partner in the **past 3 months**?
By "primary" partner we mean a man you have lived with or have seen a lot, to whom you have felt a special emotional commitment for at least 3 months. This includes times when you were a top or bottom, used or didn't use a condom, and whether or not there was ejaculation.
" is one of the following answers ("Yes")

Last Sex with Male Primary Partner - Substance Use (SXP42 - SXP44)

Logic: Show/hide trigger exists.

Shortname / Alias: mp_ydrunk

ID: 282

Were you drunk or buzzed on alcohol within two hours before or during the most recent time you had anal sex with [question('value'), id='245',case='upper']?

(1) Yes

(0) No

Validation: Must be numeric Whole numbers only Positive numbers only

Logic: Hidden unless: Question "Were you drunk or buzzed on alcohol within two hours before or during the most recent time you had anal sex with [question('value'), id='245',case='upper']?" is one of the following answers ("Yes")

Shortname / Alias: mp_ydrunk_num

ID: 675

How many drinks did you have? By a drink, I mean a 12 oz. can or glass of beer, a 4 oz. glass of wine, a 1-1/2 oz. shot of liquor, or a mixed drink with that amount of liquor.

____

Shortname / Alias: mp_pdrunk

ID: 284

Was [question('value'), id='245',case='upper'] drunk or buzzed on alcohol within two hours before or during the most recent time you had anal sex?

(1) Yes

(0) No

(7) I don't know

Page entry logic: This page will show when: Question "Have you had anal sex with a male primary partner in the **past 3 months**?
By "primary" partner we mean a man you have lived with or have seen a lot, to whom you have felt a special emotional commitment for at least 3 months. This includes times when you were a top or bottom, used or didn't use a condom, and whether or not there was ejaculation.
" is one of the following answers ("Yes")

Last Sex with Primary Partner - Substance Use (SXP45 - SXP48)

Logic: Show/hide trigger exists.

Shortname / Alias: mp_ydrugs

ID: 285

Did you use any drug that was not prescribed to you within two hours before or during the most recent time you had anal sex with [question('value'), id='245',case='upper']?

(1) Yes

(0) No

Logic: Hidden unless: Question "Did you use any drug that was not prescribed to you within two hours before or during the most recent time you had anal sex with [question('value'), id='245',case='upper']?" is one of the following answers ("Yes")

Shortname / Alias: mp_y_

ID: 286

Which drugs? Select all that apply.

[ ] Methamphetamine or other amphetamine, injected (meth, speed, crystal, crank, ice) [MP_Y_INJMETH]

[ ] Methamphetamine or other amphetamine, not injected (meth, speed, crystal, crank, ice) [MP_Y_METH]

[ ] Downers (Valium, Ativa, Xanax) [MP_Y_DOWN]

[ ] Pain killers (Oxycontin, Percocet) [MP_Y_PAIN]

[ ] Hallucinogens (LSD, mushrooms, Peyote, Mescaline) [MP_Y_LSD]

[ ] Ecstacy (E, X, MDMA) [MP_Y_ECSTACY]

[ ] Club drugs (GHB, ketamine, special K) [MP_Y_GHB]

[ ] Marijuana (pot, weed) [MP_Y_POT]

[ ] Poppers (amyl nitrate) [MP_Y_POPPERS]

[ ] PCP (angel dust, wet, wicky sticks) [MP_Y_PCP]

[ ] Synthetic marijuana (herbal incense, spice, K2) [MP_Y_SPICE]

[ ] Crack, injected [MP_Y_INJCRACK]

[ ] Crack, smoked or snorted [MP_Y_CRACK]

[ ] Cocaine, injected [MP_Y_INJCOKE]

[ ] Cocaine, smoked or snorted [MP_Y_COKE]

[ ] Heroin, injected [MP_Y_INJHEROIN]

[ ] Heroin, smoked or snorted [MP_Y_HEROIN]

[ ] Heroin and cocaine injected together (speedballs) [MP_Y_SPEED]

[ ] Other, please specify:: ____ [MP_Y_OTHER] [MP_Y_OTHERSP]

Logic: Show/hide trigger exists.

Shortname / Alias: mp_pdrugs

ID: 287

Did [question('value'), id='245',case='upper'] use any drug not prescribed to him within two hours before or during the most recent time you had sex?

(1) Yes

(0) No

(7) I don't know

Logic: Hidden unless: Question "Did [question('value'), id='245',case='upper'] use any drug not prescribed to him within two hours before or during the most recent time you had sex?" is one of the following answers ("Yes")

Shortname / Alias: mp_p

ID: 288

Which drugs? (Check all that apply)

[ ] Methamphetamine or other amphetamine, injected (meth, speed, crystal, crank, ice) [MP_P_INJMETH]

[ ] Methamphetamine or other amphetamine, not injected (meth, speed, crystal, crank, ice) [MP_P_METH]

[ ] Downers (Valium, Ativa, Xanax) [MP_P_DOWN]

[ ] Pain killers (Oxycontin, Percocet) [MP_P_PAIN]

[ ] Hallucinogens (LSD, mushrooms, Peyote, Mescaline) [MP_P_LSD]

[ ] Ecstacy (E, X, MDMA) [MP_P_ECSTACY]

[ ] Club drugs (GHB, ketamine, special K) [MP_P_GHB]

[ ] Marijuana (pot, weed) [MP_P_POT]

[ ] Poppers (amyl nitrate) [MP_P_POPPERS]

[ ] PCP (angel dust, wet, wicky sticks) [MP_P_PCP]

[ ] Synthetic marijuana (herbal incense, spice, K2) [MP_P_SPICE]

[ ] Crack, injected [MP_P_INJCRACK]

[ ] Crack, smoked or snorted [MP_P_CRACK]

[ ] Cocaine, injected [MP_P_INJCOKE]

[ ] Cocaine, smoked or snorted [MP_P_COKE]

[ ] Heroin, injected [MP_P_INJHEROIN]

[ ] Heroin, smoked or snorted [MP_P_HEROIN]

[ ] Heroin and cocaine injected together (speedballs) [MP_P_SPEED]

[ ] Other, please specify:: ____ [MP_P_OTHER] [MP_P_OTHERSP]

Page entry logic: This page will show when: Question "Now we want to ask you about all of the other men you have had any anal sex with in the **past 3 months** other than your most recent primary partner.

In the **past 3 months**, with how many men other than your most recent primary partner did you have anal sex?
By non-primary partner, we mean someone you had sex with but do not feel committed to or don't know very well. This includes times when you were a top or bottom, used or didn't use a condom, and whether or not there was ejaculation." is greater than or equal to "1"

Other Male Partners (SPS1 - SPS2)

ID: 392

The following questions ask you about the [question('value'), id='510'] non-primary male partners you have had in the past three months.

If you are unsure of any partner's HIV status, please do not include them in either group.

Logic: Hidden by default

ID: 568

Error! You reported more positive and negative partners than previously reported total other partners. If you are unsure of any partner's HIV status, please do not include them in either group.

Validation: Must be numeric Whole numbers only Positive numbers only

Shortname / Alias: other_pos_num

ID: 597

Of these [question('value'), id='510'] men, how many did you believe were HIV <u>positive</u>?

____

Validation: Must be numeric Whole numbers only Positive numbers only

Shortname / Alias: other_neg_num

ID: 598

Of these [question('value'), id='510'] men, how many did you believe were HIV <u>negative</u>?

____

Script for More Partners Than Previously Reported

Action: Custom Script: Error Message for more partners than previously reported

---

Script to calculate HIV positive and negative partners

Action: Custom Script:

Hidden Value: tot_p (# of total non-primary partners - # of non-primary partners believed to be HIV positive)

Value:

Hidden Value: tot_pn (# of total non-primary partners - # of non-primary partners believed to be HIV positive - # of non-primary partners believed to be HIV negative): # of partners with an unknown HIV status

Value:

Hidden Value: tot_pn2 (# of total non-primary partners - # of non-primary partners believed to be HIV positive - # of non-primary partners believed to be HIV negative): # of partners with an unknown HIV status

Value:

Hidden Value: sex46 (# of non-primary partners believed to be HIV positive) [TOTHIVPOSCASPART]

Value:

Hidden Value: sex47 (# of non-primary partners believed to be HIV negative) [TOTHIVNEGCASPART]

Value:

Other Male Partners (SPS3)

Page exit logic: Skip / Disqualify Logic**IF:** Question "So, you had [question("value"), id="295"] partners whose HIV status you did not know or were unsure of?" is one of the following answers ("No, I want to change my answer") **THEN:** Jump to page 68 - Other Male Partners (SPS1 - SPS2)

Shortname / Alias: CasPartHIVunk

ID: 298

So, you had [question("value"), id="295"] partners whose HIV status you did not know or were unsure of?

(1) Yes

(0) No, I want to change my answer

Page entry logic: This page will show when: sex46 (# of non-primary partners believed to be HIV positive)
is greater than or equal to "1"

Other Male Partners - HIV-Positive (SPS4)

Validation: Must be numeric Whole numbers only Positive numbers only

Shortname / Alias: pos_told_num

ID: 599

Of the [question('option value'), id='513'] HIV positive men, how many actually TOLD you they were HIV positive?

____

Page entry logic: This page will show when: sex47 (# of non-primary partners believed to be HIV negative) is greater than or equal to "1"

Other Male Partners - HIV-Negative (SPS5)

Page exit logic: Skip / Disqualify Logic**IF:** (Question "Of these [question('value'), id='510'] men, how many did you believe were HIV positive?" is exactly equal to "0" OR Question "Of these [question('value'), id='510'] men, how many did you believe were HIV positive?") **THEN:** Jump to page 81 - Other Male Partners - HIV-Negative (SXC11 - SXC12)

Validation: Must be numeric Whole numbers only Positive numbers only

Logic: Hidden unless: sex47 (# of non-primary partners believed to be HIV negative) is greater than or equal to "1"

Shortname / Alias: neg_told_num

ID: 600

Of the [question('option value'), id='514'] HIV negative men, how many actually TOLD you they were HIV negative?

____

Page entry logic: This page will show when: Question "Of these [question('value'), id='510'] men, how many did you believe were HIV positive?" is greater than or equal to "1"

Other Male Partners - HIV-Positive (SXC1 - SXC2)

ID: 305

The following questions ask about your HIV-POSITIVE partners who were NOT your primary partner.

Validation: Must be numeric Whole numbers only Positive numbers only

Shortname / Alias: pos_top_num

ID: 603

In the past 3 months, that is since [question('value'), id='760'], with how many of your [question('value'), id='513'] HIV-positive partner(s) were you the top (you put your penis in his butt)?

____

Validation: Must be numeric Whole numbers only Positive numbers only

Shortname / Alias: pos_bott_num

ID: 605

In the past 3 months, that is since [question('value'), id='760'], with how many of your [question('value'), id='513'] HIV-positive partner(s) were you the bottom (he put his penis in your butt)?

____

Page entry logic: This page will show when: Question "Of these [question('value'), id='510'] men, how many did you believe were HIV positive?" is greater than or equal to "1"

Other Male Partners - HIV-Positive (SXC3)

Page exit logic: Skip / Disqualify Logic**IF:** Question "In the **past 3 months**, that is since [question('value'), id='760'], with how many of your [question('value'), id='513'] HIV-positive partner(s) did you have ANY anal sex in which a condom was not used from start to finish?" is exactly equal to "0" **THEN:** Jump to page 79 - Other Male Partners - HIV-Positive - Partner ART Use (SXC8)

Validation: Must be numeric Whole numbers only Positive numbers only

Logic: Show/hide trigger exists.

Shortname / Alias: pos_uai_num

ID: 607

In the past 3 months, that is since [question('value'), id='760'], with how many of your [question('value'), id='513'] HIV-positive partner(s) did you have ANY anal sex in which a condom was not used from start to finish?

____

Page entry logic: This page will show when: Question "Of these [question('value'), id='510'] men, how many did you believe were HIV positive?" is greater than or equal to "1"

Other Male Partners - HIV-Positive (SXC4 - SXC7)

Validation: Must be numeric Whole numbers only Positive numbers only

Logic: Hidden unless: Question "In the past 3 months, that is since [question('value'), id='760'], with how many of your [question('value'), id='513'] HIV-positive partner(s) did you have ANY anal sex in which a condom was not used from start to finish?" is greater than or equal to "1"

Shortname / Alias: pos_uai_top

ID: 608

Of the [question('value'), id='607'] HIV-positive partner(s) you had anal sex with and did not use a condom from start to finish, with how many were you the top (you put your penis in his butt) when you had sex without a condom?

____

Validation: Must be numeric Whole numbers only Positive numbers only

Logic: Hidden unless: Question "In the past 3 months, that is since [question('value'), id='760'], with how many of your [question('value'), id='513'] HIV-positive partner(s) did you have ANY anal sex in which a condom was not used from start to finish?" is greater than or equal to "1"

Shortname / Alias: pos_uai_bot

ID: 614

Of those [question("value"), id="607"] men you had anal sex with and did not use a condom from start to finish, with how many were you the bottom (he put his penis in your butt) when you had sex without a condom?

____

Validation: Must be numeric Whole numbers only Positive numbers only

Logic: Hidden unless: Question "In the past 3 months, that is since [question('value'), id='760'], with how many of your [question('value'), id='513'] HIV-positive partner(s) did you have ANY anal sex in which a condom was not used from start to finish?" is greater than or equal to "1"

Shortname / Alias: pos_uai_drunk

ID: 616

Of the [question("value"), id="607"] men you had anal sex with and did not use a condom from start to finish, with how many did you have sex without a condom while you were drunk or buzzed on alcohol within 2 hours before or during sex?

____

Validation: Must be numeric Whole numbers only Positive numbers only

Logic: Hidden unless: Question "In the past 3 months, that is since [question('value'), id='760'], with how many of your [question('value'), id='513'] HIV-positive partner(s) did you have ANY anal sex in which a condom was not used from start to finish?" is greater than or equal to "1"

Shortname / Alias: pos_uai_drugs

ID: 618

Of the [question("value"), id="607"] men you had anal sex with and did not use a condom from start to finish, with how many did you have sex without a condom after using non-prescribed drugs within 2 hours before or during sex?

____

Page entry logic: This page will show when: sex46 (# of non-primary partners believed to be HIV positive)
is greater than or equal to "1"

Other Male Partners - HIV-Positive - Partner ART Use (SXC8)

Logic: Show/hide trigger exists.

Shortname / Alias: pos_art

ID: 316

In the past 3 months, that is since [question('value'), id='760'], have you talked to any of your HIV-positive male partners about whether they were using antiretroviral medicines to treat their HIV?

(1) Yes

(0) No

Page entry logic: This page will show when: Question "In the **past 3 months**, that is since [question('value'), id='760'], have you talked to any of your HIV-positive male partners about whether they were using antiretroviral medicines to treat their HIV?" is one of the following answers ("Yes")

Other Male Partners - HIV-Positive - Undetectable (SXC9 - SXC10)

Page exit logic: Skip / Disqualify Logic**IF:** Question "Of these [question('value'), id='510'] men, how many did you believe were HIV negative?" is exactly equal to "0" **THEN:** Jump to page 85 - Other Male Partners - HIV-Indeterminate (SXC20 - SXC21)

Validation: Must be numeric Whole numbers only Positive numbers only

Logic: Show/hide trigger exists. Hidden unless: Question "In the past 3 months, that is since [question('value'), id='760'], have you talked to any of your HIV-positive male partners about whether they were using antiretroviral medicines to treat their HIV?" is one of the following answers ("Yes")

Shortname / Alias: pos_art_current

ID: 754

Of your [question("value"), id="513"] HIV-positive partners, how many told you they were currently taking antiretroviral medicines to treat their HIV?

____

Validation: Must be numeric Whole numbers only Positive numbers only

Logic: Hidden unless: Question "Of your [question("value"), id="513"] HIV-positive partners, how many told you they were currently taking antiretroviral medicines to treat their HIV?" is greater than or equal to "1"

Shortname / Alias: pos_undetect

ID: 625

Of your [question("value"), id="513"] HIV-positive partners, how many told you they were undetectable or virally suppressed?

____

Page entry logic: This page will show when: sex47 (# of non-primary partners believed to be HIV negative) is greater than or equal to "1"

Other Male Partners - HIV-Negative (SXC11 - SXC12)

ID: 319

The following questions ask about your HIV-NEGATIVE partners who were NOT your primary partner.

Validation: Must be numeric Whole numbers only Positive numbers only

Shortname / Alias: neg_top_num

ID: 626

In the past 3 months, that is since [question('value'), id='760'], with how many of your [question('value'), id='514'] HIV-negative partners were you the top (you put your penis in his butt)?

____

Validation: Must be numeric Whole numbers only Positive numbers only

Shortname / Alias: neg_bot_num

ID: 628

In the past 3 months, that is since [question('value'), id='760'], with how many of your [question('value'), id='514'] HIV-negative partners were you the bottom (he put his penis in your butt)?

____

Page entry logic: This page will show when: sex47 (# of non-primary partners believed to be HIV negative) is greater than or equal to "1"

Other Male Partners - HIV-Negative (SXC13)

Validation: Must be numeric Whole numbers only Positive numbers only

Shortname / Alias: neg_uai_num

ID: 630

In the past 3 months, with how many of your [question('value'), id='514'] HIV-negative partners did you have ANY anal sex in which a condom was not used from start to finish?

____

Page entry logic: This page will show when: (Question "In the **past 3 months**, with how many of your [question('value'), id='514'] HIV-negative partners did you have ANY anal sex in which a condom was not used from start to finish?" is greater than or equal to "1" AND sex47 (# of non-primary partners believed to be HIV negative) is greater than or equal to "1")

Other Male Partners - HIV-Negative (SXC14 - SXC17)

Validation: Must be numeric Whole numbers only Positive numbers only

Shortname / Alias: neg_uai_top

ID: 633

Of those [question("value"), id="630"] men you had anal sex with and did not use a condom from start to finish, with how many were you the top (you put your penis in his butt) when you had sex without a condom?

____

Validation: Must be numeric Whole numbers only Positive numbers only

Shortname / Alias: neg_uai_bot

ID: 635

Of those [question("value"), id="630"] men you had anal sex with and did not use a condom from start to finish, with how many were you the bottom (he put his penis in your butt) when you had sex without a condom?

____

Validation: Must be numeric Whole numbers only Positive numbers only

Shortname / Alias: neg_uai_drunk

ID: 641

Of those [question("value"), id="630"] men you had anal sex with and did not use a condom from start to finish, with how many did you have sex without a

condom while you were drunk or buzzed on alcohol within 2 hours before or during sex?

____

Validation: Must be numeric Whole numbers only Positive numbers only

Shortname / Alias: neg_uai_drugs

ID: 643

Of those [question("value"), id="630"] men you had anal sex with and did not use a condom from start to finish, with how many did you have sex without a condom after using non-prescribed drugs within 2 hours before or during sex?

____

Page entry logic: This page will show when: sex47 (# of non-primary partners believed to be HIV negative) is greater than or equal to "1"

Other Male Partners - HIV-Negative - PrEP (SXC18 - SXC19)

Page exit logic: Skip / Disqualify Logic**IF:** tot_pn2 (# of total non-primary partners - # of non-primary partners believed to be HIV positive - # of non-primary partners believed to be HIV negative): # of partners with an unknown HIV status is exactly equal to "0" **THEN:** Jump to page 89 - Last Non-Primary Male Partner (SXCL1)

Logic: Show/hide trigger exists.

Shortname / Alias: neg_prep

ID: 327

In the past 3 months, that is since [question('value'), id='760'], have you talked to any of your HIV-negative partners about whether they are currently taking PrEP to prevent HIV?

(1) Yes

(0) No

Validation: Must be numeric Whole numbers only Positive numbers only

Logic: Hidden unless: Question "In the past 3 months, that is since [question('value'), id='760'], have you talked to any of your HIV-negative partners about whether they are currently taking PrEP to prevent HIV?" is one of the following answers ("Yes")

Shortname / Alias: neg_prep_current

ID: 646

Of your [question("value"), id="514"] HIV-negative partners, how many told you they were currently taking PrEP to prevent HIV?

____

Page entry logic: This page will show when: tot_pn2 (# of total non-primary partners - # of non-primary partners believed to be HIV positive - # of non-primary partners believed to be HIV negative): # of partners with an unknown HIV status is greater than or equal to "1"

Other Male Partners - HIV-Indeterminate (SXC20 - SXC21)

ID: 529

The following questions ask about your partners who were not your primary partner and whose HIV status you did not know.

Validation: Must be numeric Whole numbers only Positive numbers only

Shortname / Alias: unk_top_num

ID: 647

In the past 3 months, that is since [question('value'), id='760'], with how many of your [question('value'), id='295'] partners whose HIV status you did not know were you the top (you put your penis in his butt)?

____

Validation: Must be numeric Whole numbers only Positive numbers only

Shortname / Alias: unk_bot_num

ID: 649

In the past 3 months, that is since [question('value'), id='760'], with how many of your [question('value'), id='295'] partners whose HIV status you did not know were you the bottom (he put his penis in your butt)?

____

Page entry logic: This page will show when: tot_pn2 (# of total non-primary partners - # of non-primary partners believed to be HIV positive - # of non-primary partners believed to be HIV negative): # of partners with an unknown HIV status is greater than or equal to "1"

Non-Primary Partners - HIV-Indeterminate

Validation: Must be numeric Whole numbers only Positive numbers only

Shortname / Alias: unk_uai_num

ID: 651

In the past 3 months, that is since [question('value'), id='760'], with how many of your [question('value'), id='295'] partners whose HIV status you did not know did you have ANY anal sex in which a condom was not used from start to finish?

____

Page entry logic: This page will show when: Question "In the **past 3 months**, that is since [question('value'), id='760'], with how many of your [question('value'), id='295'] partners whose HIV status you did not know did you have ANY anal sex in which a condom was not used from start to finish?" is greater than or equal to "1"

Non-Primary Partners - HIV-Indeterminate

Validation: Must be numeric Whole numbers only Positive numbers only

Shortname / Alias: unk_uai_top

ID: 654

Of those [question("value"), id="651"] men you had anal sex with and did not use a condom from start to finish, with how many were you the top (you put your penis in his butt) when you had sex without a condom?

____

Validation: Must be numeric Whole numbers only Positive numbers only

Shortname / Alias: unk_uai_bot

ID: 656

Of those [question("value"), id="651"] men you had anal sex with and did not use a condom from start to finish, with how many were you the bottom (he put his penis in your butt) when you had sex without a condom?

____

Validation: Must be numeric Whole numbers only Positive numbers only

Shortname / Alias: unk_uai_drunk

ID: 658

Of those [question("value"), id="651"] men you had anal sex with and did not use a condom from start to finish, with how many did you have sex without a condom while you were drunk or buzzed on alcohol within 2 hours before or during sex?

____

Validation: Must be numeric Whole numbers only Positive numbers only
Shortname / Alias: unk_uai_drugs
ID: 660

Of those [question("value"), id="651"] men you had anal sex with and did not use a condom from start to finish, with how many did you have sex without a condom after using non-prescribed drugs within 2 hours before or during sex?

____

Page entry logic: This page will show when: Question "Now we want to ask you about all of the other men you have had any anal sex with in the **past 3 months** other than your most recent primary partner.

In the **past 3 months**, with how many men other than your most recent primary partner did you have anal sex?
By non-primary partner, we mean someone you had sex with but do not feel committed to or don’t know very well. This includes times when you were a top or bottom, used or didn't use a condom, and whether or not there was ejaculation." is greater than or equal to "1"

Last Non-Primary Male Partner (SXCL1)

ID: 336

You will now be asked more detailed questions about the last man you had any anal sex with in the past THREE months who was not your male primary partner.

That is, not someone you have lived with or have seen a lot, and to whom you have felt a special emotional commitment. Think about the very last man you had any anal sex with in the last THREE months who was not your primary partner.

Validation: Min character count = 1
Shortname / Alias: op_nicknm
ID: 337

Throughout this next group of questions, we will ask you about the last man you had any anal sex with in the past THREE months who was not your male primary partner using a nickname you have provided. Examples of a nickname that you might choose are: a partner's first name, a nickname you call the partner by, the place where you both met, or an online screen name.

Please choose a nickname that will best help you know to whom we are referring. The nickname is to make the survey easier to take and you should not reveal this man's full identity. We do not wish to know who your partner actually is.

Enter the nickname for the last man you had any anal sex with in the past THREE months who was not your male primary partner below. *

____

Page entry logic: This page will show when: Question "Now we want to ask you about all of the other men you have had any anal sex with in the **past 3 months** other than your most recent primary partner.

In the **past 3 months**, with how many men other than your most recent primary partner did you have anal sex?
By non-primary partner, we mean someone you had sex with but do not feel committed to or don't know very well. This includes times when you were a top or bottom, used or didn't use a condom, and whether or not there was ejaculation." is greater than or equal to "1"

Last Non-Primary Male Partner (SXCL2 - SXCL8)

Page exit logic: Skip / Disqualify Logic**IF:** Question "In the **past 3 months**, that is since [question('value'),id='760'], have you had ANY anal sex with **[question('value'), id='337',case='upper']** in which a condom was not used from start to finish?" is one of the following answers ("No") **THEN:** Jump to page 92 - Last Non-Primary Male Partner - HIV Status (SXCL12 - SXCL14)

Shortname / Alias: op_age

ID: 534

How old is [question('value'), id='337',case='upper']? If you are not sure, give it your best guess.

____

Shortname / Alias: op_race

ID: 340

What is [question('value'), id='337',case='upper']'s primary race/ethnicity? If you are not sure, give it your best guess.

(1) African American or Black

(2) Asian American or Pacific Islander

(3) Latino, Hispanic, or Chicano

(4) Native American, American Indian, or Alaska Native

(5) White or European American

(6) Mixed

(7) Other, please specify:: ____

Shortname / Alias: op_met

ID: 672

Where did you first meet [question('value'), id='337',case='upper']? (Choose one)

(1) A dating or hookup app/website for gay men, like Grindr or Jack'd

(2) A social networking app or website for gay men (not for dating or hooking up)

(3) A social media app or website, like Facebook, Twitter, Instagram or Snapchat

(4) Some other app or website

(5) Work or school

(6) Through family

(7) Through a friend

(8) A private party or social event

(9) A public festival or community event, like Pride

(10) A gay commercial venue, like a gay bar or dance club

(11) A straight commercial venue, like a straight bar or dance club

(12) A commercial sex venue, like a sex club or bath house

(13) A public sex venue, like a park, beach, bathroom or tearoom

(14) Other, please specify:: ____

Shortname / Alias: op_1sex

ID: 343

When was the first time you had any anal sex with [question('value'), id='337',case='upper']? That is, where you were a top or bottom, with or without a condom, and with or without ejaculation. (Choose one)

(1) Less than a week ago

(2) More than a week but less than one month ago

(3) One to three months ago

(4) Four to six months ago

(5) Seven to twelve months ago

(6) More than a year ago

Shortname / Alias: op_uai

ID: 344

In the past 3 months, that is since [question('value'),id='760'], have you had ANY anal sex with [question('value'), id='337',case='upper'] in which a condom was not used from start to finish?

(1) Yes

(0) No

(7) I don't know

Page entry logic: This page will show when: (Question "In the **past 3 months**, that is since [question('value'),id='760'], have you had ANY anal sex with **[question('value'), id='337',case='upper']** in which a condom was not used from start to finish?" is one of the following answers ("Yes") AND Question "Now we want to ask you about all of the other men you have had any anal sex with in the **past 3 months** other than your most recent primary partner.

In the **past 3 months**, with how many men other than your most recent primary partner did you have anal sex?
By non-primary partner, we mean someone you had sex with but do not feel committed to or don't know very well. This includes times when you were a top or bottom, used or didn't use a condom, and whether or not there was ejaculation." is greater than or equal to "1")

Last Non-Primary Male Partner (SXCL9 - SXCL11)

Shortname / Alias: op_uai_position

ID: 345

Think of the times in the past 3 months that you had anal sex with [question('value'), id='337',case='upper'] and did not use a condom from start to finish. Were you the top (you put your penis in his butt), the bottom (he put his penis in your butt), or both?

(1) I was always the top

(2) I was always the bottom

(3) I was both the top and the bottom

Shortname / Alias: op_uai_drunk

ID: 346

Think of the times in the past 3 months that you had anal sex with [question('value'), id='337',case='upper'] and did not use a condom from start to finish. Were you ever drunk or buzzed on alcohol within 2 hours before or during sex?

(1) Yes

(0) No

Shortname / Alias: op_uai_drugs

ID: 347

Think of the times in the past 3 months that you had anal sex with [question('value'), id='337',case='upper'] and did not use a condom from start to finish. Did you ever use non-prescription drugs within 2 hours before or during sex?

(1) Yes

(0) No

Page entry logic: This page will show when: Question "Now we want to ask you about all of the other men you have had any anal sex with in the **past 3 months** other than your most recent primary partner.

In the **past 3 months**, with how many men other than your most recent primary partner did you have anal sex?
By non-primary partner, we mean someone you had sex with but do not feel committed to or don’t know very well. This includes times when you were a top or bottom, used or didn't use a condom, and whether or not there was ejaculation." is greater than or equal to "1"

Last Non-Primary Male Partner - HIV Status (SXCL12 - SXCL14)

Logic: Show/hide trigger exists.

Shortname / Alias: op_status

ID: 348

What is [question('value'), id='337',case='upper']'s HIV status?

(1) Positive

(2) Negative

(7) I don't know

Shortname / Alias: op_ptell

ID: 349

Did [question('value'), id='337',case='upper'] actually TELL you his HIV status?

(1) Yes

(0) No

Shortname / Alias: op_ytold

ID: 351

Did you tell [question('value'), id='337',case='upper'] your current HIV status before the last time you had anal sex together?

(1) Yes

(0) No

Page entry logic: This page will show when: Question "Now we want to ask you about all of the other men you have had any anal sex with in the **past 3 months** other than your most recent primary partner.

In the **past 3 months**, with how many men other than your most recent primary partner did you have anal sex?
By non-primary partner, we mean someone you had sex with but do not feel committed to or don’t know very well. This includes times when you were a top or bottom, used or didn't use a condom, and whether or not there was ejaculation." is greater than or equal to "1"

Last Non-Primary Male Partner (SXCL15 - SXCL16)

Shortname / Alias: op_last_ai

ID: 350

How long ago was the last time you had any anal sex with [question('value'), id='337',case='upper']? That is, where you were a top or a bottom, with or without a condom, and with or without ejaculation.

(1) 1 day

(2) 2 days

(3) 3 days

(4) 4 days

(5) 5 days

(6) 6 days

(7) 1 week

(8) 2 weeks

(9) 3 weeks

(10) 1 month

(11) 2 months

(12) 3 months

(13) More than 3 months

Shortname / Alias: op_last_where

ID: 352

During the last time you had any anal sex with [question('value'), id='337',case='upper'], where did you have sex?

(1) Your house or apartment or his house or apartment

(2) Someone else's house or apartment

(3) Hotel

(4) Sex club or bathhouse

(5) Bar, night club, or dance club

(6) Health club or gym

(7) Porn theatre/video arcade

(8) Public bathroom

(9) Other public place, such as beach

(10) Park, woods, street, car or van

(11) Other, please specify:: ____

Page entry logic: This page will show when: Question "Now we want to ask you about all of the other men you have had any anal sex with in the **past 3 months** other than your most recent primary partner.

In the **past 3 months**, with how many men other than your most recent primary partner did you have anal sex?
By non-primary partner, we mean someone you had sex with but do not feel committed to or don’t know very well. This includes times when you were a top or bottom, used or didn't use a condom, and whether or not there was ejaculation." is greater than or equal to "1"

Last Non-Primary Male Partner (SXCL19 - SXCL22)

Page exit logic: Skip / Disqualify Logic**IF:** Question "What is your HIV status?" is one of the following answers ("HIV Positive") **THEN:** Jump to page 96 - Last Non-Primary Male Partner - Partner PrEP (SXCL26 - SXCL28)

Page exit logic: Skip / Disqualify Logic**IF:** Question "Have you ever used PrEP to prevent HIV?" is one of the following answers ("No") **THEN:** Jump to page 96 - Last Non-Primary Male Partner - Partner PrEP (SXCL26 - SXCL28)

Logic: Show/hide trigger exists.

Shortname / Alias: op_top

ID: 355

During the last time you had anal sex with [question('value'), id='337',case='upper'], were you the top (you put your penis in his butt)? This would be with or without a condom and with or without ejaculation.

(1) Yes

(0) No

**Logic: Hidden unless: Question "During the last time you had anal sex with [question('value'), id='337',case='upper'], were you the top (you put your penis in his butt)? This would be with or without a condom and with or without ejaculation." is one of the following answers ("Yes")**
Shortname / Alias: op_top_condom
ID: 356

Was a condom used from start to finish?

(1) Yes

(0) No

(7) I don't know

**Logic: Show/hide trigger exists.**
Shortname / Alias: op_bottom
ID: 357

During the last time you had anal sex with [question('value'), id='337',case='upper'], were you the bottom (he put his penis in your butt)? This would be with or without a condom and whether or not you ejaculated.

(1) Yes

(0) No

**Logic: Hidden unless: Question "During the last time you had anal sex with [question('value'), id='337',case='upper'], were you the bottom (he put his penis in your butt)? This would be with or without a condom and whether or not you ejaculated." is one of the following answers ("Yes")**

Shortname / Alias: op_bottom_condom

ID: 358

Was a condom used from start to finish?

(1) Yes

(0) No

(7) I don't know

Page entry logic: This page will show when: Question "Now we want to ask you about all of the other men you have had any anal sex with in the **past 3 months** other than your most recent primary partner.

In the **past 3 months**, with how many men other than your most recent primary partner did you have anal sex?
By non-primary partner, we mean someone you had sex with but do not feel committed to or don't know very well. This includes times when you were a top or bottom, used or didn't use a condom, and whether or not there was ejaculation." is greater than or equal to "1"

Last Non-Primary Male Partner - Recent PrEP (SXCL23 - SXCL25)

Page exit logic: Skip / Disqualify Logic**IF:** Question "What is **[question('value'), id='337',case='upper']'s** HIV status?" is one of the following answers ("Positive") **THEN:** Jump to page 97 - Last Non-Primary Male Partner - Recent ART (SXCL29 - SXCL31)

Logic: Show/hide trigger exists.

Shortname / Alias: op_yprep24

ID: 359

Were you taking PrEP medication to prevent HIV within 24 hours before or after the time you last had anal sex with [question('value'), id='337',case='upper']?

(1) Yes

(0) No

(7) I don’t know

Logic: Show/hide trigger exists. Hidden unless: Question "Were you taking PrEP medication to prevent HIV within 24 hours before or after the time you last had anal sex with [question('value'), id='337',case='upper']?" is one of the following answers ("Yes")

Shortname / Alias: op_yprep7

ID: 360

Were you taking your PrEP medication once a day as prescribed by your doctor, without missing doses, in the 7 days before the time you last had anal sex with [question('value'), id='337',case='upper']?

(1) Yes

(0) No

(7) I don’t know

Logic: Hidden unless: Question "Were you taking your PrEP medication once a day as prescribed by your doctor, without missing doses, in the 7 days before the time you last had anal sex with [question('value'), id='337',case='upper']?" is one of the following answers ("No")

Shortname / Alias: op_yprepmiss

ID: 662

You mentioned you were not taking your PrEP medication once a day as prescribed at the time of your last sexual encounter. In the 7 days before you last had anal sex with [question('value'), id='337',case='upper'], on how many days did you take your dose of PrEP?

(7) 7

(6) 6

(5) 5

(4) 4

(3) 3

(2) 2

(1) 1

(0) 0

(77) I don't know

(99) I'd prefer not to answer

Page entry logic: This page will show when: Question "Now we want to ask you about all of the other men you have had any anal sex with in the **past 3 months** other than your most recent primary partner.

In the **past 3 months**, with how many men other than your most recent primary partner did you have anal sex?
By non-primary partner, we mean someone you had sex with but do not feel committed to or don't know very well. This includes times when you were a top or bottom, used or didn't use a condom, and whether or not there was ejaculation." is greater than or equal to "1"

Last Non-Primary Male Partner - Partner PrEP (SXCL26 - SXCL28)

Page exit logic: Skip / Disqualify Logic**IF:** (Question "What is your HIV status?" is one of the following answers ("HIV Negative") OR Question "Have you ever been prescribed and taken antiretroviral medications to treat your HIV?" is one of the following answers ("No")) **THEN:** Jump to page 99 - Last Non-Primary Male Partner - Substance Use (SXCL37 - SXCL39)

Logic: Show/hide trigger exists. Hidden unless: Question "What is [question('value'), id='337',case='upper']'s HIV status?" is one of the following answers ("Negative")

Shortname / Alias: op_pprep24

ID: 361

Was [question('value'), id='337',case='upper'] taking PrEP medication to prevent HIV within 24 hours before or after the time you last had anal sex?

(1) Yes

(0) No

(7) I don't know

**Logic: Show/hide trigger exists. Hidden unless: Question "Was [question('value'), id='337',case='upper'] taking PrEP medication to prevent HIV within 24 hours before or after the time you last had anal sex?" is one of the following answers ("Yes")**
Shortname / Alias: op_pprep7
ID: 362

To your knowledge, was [question('value'), id='337',case='upper'] taking his PrEP medication once a day as prescribed by his doctor, without missing doses, in the 7 days before the time you last had anal sex?

(1) Yes

(0) No

(7) I don’t know

**Logic: Hidden unless: Question "To your knowledge, was [question('value'), id='337',case='upper'] taking his PrEP medication once a day as prescribed by his doctor, without missing doses, in the 7 days before the time you last had anal sex?" is one of the following answers ("No")**
Shortname / Alias: op_pprepmiss
ID: 665

You mentioned that [question('value'), id='337',case='upper'] was not taking his PrEP medication once a day as prescribed at the time of your last sexual encounter. To your knowledge, in the 7 days before you last had anal sex, on how many days did [question('value'), id='337',case='upper'] take his doses of PrEP?

(7) 7

(6) 6

(5) 5

(4) 4

(3) 3

(2) 2

(1) 1

(0) 0

(77) I don't know

(99) I'd prefer not to answer

Page entry logic: This page will show when: Question "Now we want to ask you about all of the other men you have had any anal sex with in the **past 3 months** other than your most recent primary partner.

In the **past 3 months**, with how many men other than your most recent primary partner did you have anal sex?
By non-primary partner, we mean someone you had sex with but do not feel committed to or don’t know very well. This includes times when you were a top or bottom, used or didn't use a condom, and whether or not there was ejaculation." is greater than or equal to "1"

Last Non-Primary Male Partner - Recent ART (SXCL29 - SXCL31)

Page exit logic: Skip / Disqualify Logic**IF:** Question "What is **[question('value'), id='337',case='upper']'s** HIV status?" is one of the following answers ("Negative") **THEN:** Jump to page 99 - Last Non-Primary Male Partner - Substance Use (SXCL37 - SXCL39)

Logic: Show/hide trigger exists.

Shortname / Alias: op_yart24

ID: 666

Were you taking antiretroviral medicines to treat your HIV infection within 24 hours before or after you last had anal sex with [question('value'), id='337',case='upper']?

(1) Yes

(0) No

(7) I don't know

Logic: Show/hide trigger exists. Hidden unless: Question "Were you taking antiretroviral medicines to treat your HIV infection within 24 hours before or after you last had anal sex with [question('value'), id='337',case='upper']?" is one of the following answers ("Yes")

Shortname / Alias: op_yart7

ID: 667

Were you taking your HIV medicines exactly as prescribed by your doctor, without missing doses, in the 7 days before the time you last had anal sex with [question('value'), id='337',case='upper']?

(1) Yes

(0) No

(7) I don't know

Logic: Hidden unless: Question "Were you taking your HIV medicines exactly as prescribed by your doctor, without missing doses, in the 7 days before the time you last had anal sex with [question('value'), id='337',case='upper']?" is one of the following answers ("No")

Shortname / Alias: op_yartmiss

ID: 668

You mentioned you were not taking your HIV medicines exactly as prescribed at the time of your last sexual encounter. In the 7 days before you last had sex with [question('value'), id='337',case='upper'], on how many days did you take all the doses of your HIV medicines?

(7) 7

(6) 6

(5) 5

(4) 4

(3) 3

(2) 2

(1) 1

(0) 0

(77) I don’t know

(99) I’d prefer not to answer

Page entry logic: This page will show when: Question "Now we want to ask you about all of the other men you have had any anal sex with in the **past 3 months** other than your most recent primary partner.

In the **past 3 months**, with how many men other than your most recent primary partner did you have anal sex?
By non-primary partner, we mean someone you had sex with but do not feel committed to or don't know very well. This includes times when you were a top or bottom, used or didn't use a condom, and whether or not there was ejaculation." is greater than or equal to "1"

Last Non-Primary Male Partner - Partner ART (SXCL32 - SXCL36)

Logic: Show/hide trigger exists. Hidden unless: Question "What is [question('value'), id='337',case='upper']'s HIV status?" is one of the following answers ("Positive")

Shortname / Alias: op_part24

ID: 363

Was [question('value'), id='337',case='upper'] taking antiretroviral medicines to treat his HIV infection within 24 hours before or after the time you last had anal sex?

(1) Yes

(0) No

(7) I don't know

Logic: Show/hide trigger exists. Hidden unless: Question "Was [question('value'), id='337',case='upper'] taking antiretroviral medicines to treat his HIV infection within 24 hours before or after the time you last had anal sex?" is one of the following answers ("Yes")

Shortname / Alias: op_part7

ID: 364

To your knowledge, was [question('value'), id='337',case='upper'] taking his HIV medicines exactly as prescribed by his doctor, without missing doses, in the 7 days before the time you last had anal sex?

(1) Yes

(0) No

(7) I don't know

Logic: Hidden unless: Question "To your knowledge, was [question('value'), id='337',case='upper'] taking his HIV medicines exactly as prescribed by his doctor, without missing doses, in the 7 days before the time you last had anal sex?" is one of the following answers ("No")

Shortname / Alias: op_partmiss

ID: 669

You mentioned [question('value'), id='337',case='upper'] was not taking his HIV medicines exactly as prescribed at the time of your last sexual encounter. To your knowledge, in the 7 days before you last had anal sex, on how many days did [question('value'), id='337',case='upper'] take all the doses of his HIV medicines?

(7) 7

(6) 6

(5) 5

(4) 4

(3) 3

(2) 2

(1) 1

(0) 0

(77) I don't know

(99) I'd prefer not to answer

Logic: Show/hide trigger exists. Hidden unless: Question "Was [question('value'), id='337',case='upper'] taking antiretroviral medicines to treat his HIV infection within 24 hours before or after the time you last had anal sex?" is one of the following answers ("Yes")

Shortname / Alias: op_ai_vl

ID: 365

Did [question('value'), id='337',case='upper'] tell you his viral load was undetectable prior to your last sexual encounter?

(1) Yes

(0) No

**Logic: Hidden unless: Question "Did [question('value'), id='337',case='upper'] tell you his viral load was undetectable prior to your last sexual encounter?" is one of the following answers ("Yes")**
Shortname / Alias: op_aivl_when
ID: 670

When was the last time [question('value'), id='337',case='upper'] told you his viral load was undetectable?

(1) Less than a week ago

(2) More than a week but less than a month ago

(3) One to three months ago

(4) Four to six months ago

(5) Seven to twelve months ago

(6) More than a year ago

Page entry logic: This page will show when: Question "Now we want to ask you about all of the other men you have had any anal sex with in the **past 3 months** other than your most recent primary partner.

In the **past 3 months**, with how many men other than your most recent primary partner did you have anal sex?
By non-primary partner, we mean someone you had sex with but do not feel committed to or don’t know very well. This includes times when you were a top or bottom, used or didn't use a condom, and whether or not there was ejaculation." is greater than or equal to "1"

Last Non-Primary Male Partner - Substance Use (SXCL37 - SXCL39)

**Logic: Show/hide trigger exists.**
Shortname / Alias: op_ydrunk

ID: 366

Were you drunk or buzzed on alcohol within two hours before or during the last time you had anal sex with [question('value'), id='337',case='upper']?

(1) Yes

(0) No

Validation: Must be numeric Whole numbers only Positive numbers only

Logic: Hidden unless: Question "Were you drunk or buzzed on alcohol within two hours before or during the last time you had anal sex with [question('value'), id='337',case='upper']?" is one of the following answers ("Yes")

Shortname / Alias: op_ydrunk_num

ID: 535

How many drinks did you have? By a drink, I mean a 12 oz. can or glass of beer, a 4 oz. glass of wine, a 1-1/2 oz. shot of liquor, or a mixed drink with that amount of liquor.

____

Shortname / Alias: op_pdrunk

ID: 368

Was [question('value'), id='337',case='upper'] drunk or buzzed on alcohol within two hours before or during the most recent time you had anal sex?

(1) Yes

(0) No

(7) I don't know

Page entry logic: This page will show when: Question "Now we want to ask you about all of the other men you have had any anal sex with in the **past 3 months** other than your most recent primary partner.

In the **past 3 months**, with how many men other than your most recent primary partner did you have anal sex?
By non-primary partner, we mean someone you had sex with but do not feel committed to or don't know very well. This includes times when you were a top or bottom, used or didn't use a condom, and whether or not there was ejaculation." is greater than or equal to "1"

Last Non-Primary Male Partner (SXCL40 - SXCL45)

Logic: Show/hide trigger exists.

Shortname / Alias: op_ydrugs

ID: 369

Did you use any drug that was not prescribed to you within two hours before or during the most recent time you had anal sex with [question('value'), id='337',case='upper']?

(1) Yes

(0) No

Logic: Hidden unless: Question "Did you use any drug that was not prescribed to you within two hours before or during the most recent time you had anal sex with [question('value'), id='337',case='upper']?" is one of the following answers ("Yes")

Shortname / Alias: op_y

ID: 370

Which drugs? (Check all that apply)

[ ] Methamphetamine or other amphetamine, injected (meth, speed, crystal, crank, ice) [OP_Y_INJMETH]

[ ] Methamphetamine or other amphetamine, not injected (meth, speed, crystal, crank, ice) [OP_Y_METH]

[ ] Downers (Valium, Ativa, Xanax) [OP_Y_DOWN]

[ ] Pain killers (Oxycontin, Percocet) [OP_Y_PAIN]

[ ] Hallucinogens (LSD, mushrooms, Peyote, Mescaline) [OP_Y_LSD]

[ ] Ecstacy (E, X, MDMA) [OP_Y_ECSTACY]

[ ] Club drugs (GHB, ketamine, special K) [OP_Y_GHB]

[ ] Marijuana (pot, weed) [OP_Y_POT]

[ ] Poppers (amyl nitrate) [OP_Y_POPPERS]

[ ] PCP (angel dust, wet, sicky sticks) [OP_Y_PCP]

[ ] Synthetic marijuana (herbal incense, spice, K2) [OP_Y_SPICE]

[ ] Crack, injected [OP_Y_INJCRACK]

[ ] Crack, smoked or snorted [OP_Y_CRACK]

[ ] Cocaine, injected [OP_Y_INJCOKE]

[ ] Cocaine, smoked or snorted [OP_Y_COKE]

[ ] Heroin, injected [OP_Y_INJHEROIN]

[ ] Heroin, smoke or snorted [OP_Y_HEROIN]

[ ] Heroin and cocaine injected together (speedballs) [OP_Y_SPEED]

[ ] Other, please specify:: ____
[OP_Y_OTHER] [OP_Y_OTHERSP]

**Logic: Show/hide trigger exists.**
Shortname / Alias: op_pdrugs
ID: 371

Did [question('value'), id='337',case='upper'] use any drug that was not prescribed to him within two hours before or during the most recent time you had sex?

(1) Yes

(0) No

(7) I don't know

**Logic: Hidden unless: Question "Did [question('value'), id='337',case='upper'] use any drug that was not prescribed to him within two hours before or during the most recent time you had sex?" is one of the following answers ("Yes")**
Shortname / Alias: op_p
ID: 372

Which drugs? (Check all that apply)

[ ] Methamphetamine or other amphetamine, injected (meth, speed, crystal, crank, ice) [OP_P_INJMETH]

[ ] Methamphetamine or other amphetamine, not injected (meth, speed, crystal, crank, ice) [OP_P_METH]

[ ] Downers (Valium, Ativa, Xanax) [OP_P_DOWN]

[ ] Pain killers (Oxycontin, Percocet) [OP_P_PAIN]

[ ] Hallucinogens (LSD, mushrooms, Peyote, Mescaline) [OP_P_LSD]

[ ] Ecstacy (E, X, MDMA) [OP_P_ECSTACY]

[ ] Club drugs (GHB, ketamine, special K) [OP_P_GHB]

[ ] Marijuana (pot, weed) [OP_P_POT]

[ ] Poppers (amyl nitrate) [OP_P_POPPERS]

[ ] PCP (angel dust, wet, sicky sticks) [OP_P_PCP]

[ ] Synthetic marijuana (herbal incense, spice, K2) [OP_P_SPICE]

[ ] Crack, injected [OP_P_INJCRACK]

[ ] Crack, smoked or snorted [OP_P_CRACK]

[ ] Cocaine, injected [OP_P_INJCOKE]

[ ] Cocaine, smoked or snorted [OP_P_COKE]

[ ] Heroin, injected [OP_P_INJHEROIN]

[ ] Heroin, smoke or snorted [OP_P_HEROIN]

[ ] Heroin and cocaine injected together (speedballs) [OP_P_SPEED]

[ ] Other, please specify:: ____ [OP_P_OTHER] [OP_P_OTHERSP]

Sex with Women (SXW1)

Page exit logic: Skip / Disqualify Logic**IF:** Question "In the **past 3 months**, that is since [question('value'),id='760'], with how many females have you had sex? Include only females with whom you had vaginal or anal sex, with or without a condom, and with or without ejaculation." is exactly equal to "0" **THEN:** Jump to page 103 - Substance Use and Substance Dependency (SU1)

Validation: Must be numeric Whole numbers only Positive numbers only

Shortname / Alias: fem_sex_num

ID: 536

In the past 3 months, that is since [question('value'),id='760'], with how many females have you had sex? Include only females with whom you had vaginal or anal sex, with or without a condom, and with or without ejaculation.

____

Page entry logic: This page will show when: Question "In the **past 3 months**, that is since [question('value'),id='760'], with how many females have you had sex? Include only females with whom you had vaginal or anal sex, with or without a condom, and with or without ejaculation." is greater than or equal to "1"

Sex with Women (SXW2 - SXW5)

Validation: Must be numeric Whole numbers only Positive numbers only

Shortname / Alias: fem_uai_num

ID: 374

Of the [question('value'), id='536'] females you have had sex with in the past 3 months, with how many did you have any sex in which a condom was not used from start to finish?

____

Shortname / Alias: fem_primary

ID: 375

Have you been in a primary relationship with a female in the last 3 months, that is since [question('value'), id='760']? This would be a female you have lived with or have seen a lot, and to whom you have felt a special emotional commitment in the last three months.

(1) Yes
(0) No

Shortname / Alias: fem_primary_now
ID: 376

Are you still in this primary relationship with your most recent primary female partner?

(1) Yes
(0) No

Shortname / Alias: fem_status
ID: 377

What is your most recent female primary partner's HIV status?

(1) HIV Positive
(2) HIV Negative
(7) I don't know

Substance Use and Substance Dependency (SU1)

ID: 147

The following questions ask about your use of alcohol in the past year. Please select the response that best describes your use of alcohol.

Shortname / Alias: drink_often
ID: 146

How often do you have a drink containing alcohol?

(0) Never

(1) Monthly or less

(2) 2-4 times per month

(3) 2-3 times per week

(4) 4 or more times per week

Page entry logic: This page will show when: Question "How often do you have a drink containing alcohol?" is not one of the following answers ("Never")

Substance Use and Substance Dependency (SU1)

Shortname / Alias: drink_num

ID: 149

How many drinks containing alcohol do you have on a typical day when you are drinking?

(0) 1 or 2

(1) 3 or 4

(2) 5 or 6

(3) 7 to 9

(4) 10 or more

Shortname / Alias: drink_

ID: 792

	Never (0)	Less than Monthly (1)	Monthly (2)	Weekly (3)	Daily or almost daily (4)

How often do you have six or more drinks on one occasion? [DRINK_6MORE]	( )	( )	( )	( )	( )
How often during the last year have you failed to do what was normally expected of you because of drinking? [DRINK_FAILED]	( )	( )	( )	( )	( )
How often during the last year have you needed a first drink in the morning to get yourself going after a heavy drinking session? [DRINK_HEAVY]	( )	( )	( )	( )	( )
How often in the last year have you had a feeling of guilt or remorse after drinking? [DRINK_GUILT]	( )	( )	( )	( )	( )
How often in the last year have you been unable to remember what happened the night before because of your drinking? [DRINK_FORGET]	( )	( )	( )	( )	( )

Shortname / Alias: drink_injury

ID: 156

Have you or someone else been injured because of your drinking?

(0) No

(1) Yes, but not in the last year

(2) Yes, during the last year

Shortname / Alias: drink_concern

ID: 157

Has a relative, friend, doctor or other health care worker been concerned about your drinking or suggested you cut down?

(0) No

(1) Yes, but not in the last year

(2) Yes, during the last year

Substance Use and Substance Dependency (SU2 - SU4)

Logic: Show/hide trigger exists.

Shortname / Alias: drug_use

ID: 158

In the past 3 months, that is since [question('value'), id='760'], have you used any drugs that were not prescribed to you?

(1) Yes

(0) No

Logic: Hidden unless: Question "In the past 3 months, that is since [question('value'), id='760'], have you used any drugs that were not prescribed to you?" is one of the following answers ("Yes")

Shortname / Alias: drug_

ID: 159

Which of the following drugs have you used in the past 3 months? Select all that apply.

[ ] Methamphetamine or other amphetamine, injected (meth, speed, crystal, crank, ice) [DRUG_INJMETH]

[ ] Methamphetamine or other amphetamine, not injected (meth, speed, crystal, crank, ice) [DRUG_METH]

[ ] Downers (Valium, Ativa, Xanax) [DRUG_DOWN]

[ ] Pain killers (Oxycontin, Percocet) [DRUG_PAIN]

[ ] Hallucinogens (LSD, mushrooms, Peyote, Mescaline) [DRUG_LSD]

[ ] Ecstacy (E, X, MDMA) [DRUG_ECSTACY]

[ ] Club drugs (GHB, ketamine, special K) [DRUG_GHB]

[ ] Marijuana (pot, weed) [DRUG_POT]

[ ] Poppers (amyl nitrate) [DRUG_POPPERS]

[ ] PCP (angel dust, wet, wicky sticks) [DRUG_PCP]

[ ] Synthetic marijuana (herbal incense, spice, K2) [DRUG_SPICE]

[ ] Crack, injected [DRUG_INJCRACK]

[ ] Crack, smoked or snorted [DRUG_CRACK]

[ ] Cocaine, injected [DRUG_INJCOKE]

[ ] Cocaine, smoked or snorted [DRUG_COKE]

[ ] Heroin, injected [DRUG_INJHEROIN]

[ ] Heroin, smoked or snorted [DRUG_HEROIN]

[ ] Heroin and cocain injected together (speedballs) [DRUG_SPEED]

[ ] Other, please specify:: ____ [DRUG_OTHER] [DRUG_OTHERSP]

Substance Use and Substance Dependency (SU5)

ID: 160

The following questions ask about your use of drugs in the past year. Please select the response that best describes your use of drugs.

Shortname / Alias: drug_

ID: 798

	**Never (0)**	**Monthly or less (1)**	**2-4 times per month (2)**	**2-3 times per week (3)**	**4 or more times per week (4)**
How often do you use drugs other than alcohol? [DRUG_FREQ]	( )	( )	( )	( )	( )

Page entry logic: This page will show when: Question "How often do you use drugs other than alcohol?" is not one of the following answers ("Never")

Substance Use and Substance Dependency (SU5)

Shortname / Alias: drug_multiple

ID: 822

	Never (0)	Monthly or less (1)	2-4 times per month (2)	2-3 times per week (3)	4 or more times per week (4)
Do you use more than one type of drug on the same occasion? [DRUG_MULTIPLE]	( )	( )	( )	( )	( )

Shortname / Alias: drug_num

ID: 162

How many times do you take drugs on a typical day when you do drugs?

(0) 1 or 2

(1) 3 or 4

(2) 5 or 6

(3) 7 to 9

(4) 10 or more

Shortname / Alias: drug_

ID: 801

	Never (0)	Less than monthly (1)	Mont hly (2)	Weekly (3)	Daily or almost daily (4)
How often are you influenced heavily by	( )	( )	( )	( )	( )

drugs? [DRUG_INFLUENCE]					
Over the past year, have you felt that your longing for drugs was so strong that you could not resist it? [DRUG_LONGING]	( )	( )	( )	( )	( )
Has it happened, over the past year, that you have not been able to stop taking drugs once you started? [DRUG_NOSTOP]	( )	( )	( )	( )	( )
How often over the past year have you taken drugs and then neglected to do something	( )	( )	( )	( )	( )

you should have done? [DRUG_NEGLECT]					
How often over the past year have you needed to take a drug in the morning? [DRUG_MORNING]	( )	( )	( )	( )	( )
How often over the past year have you had guilty feelings or a bad conscience because you used drugs? [DRUG_GUILTY]	( )	( )	( )	( )	( )

Shortname / Alias: drug_hurt

ID: 168

Have you or anyone else been hurt (mentally or physically) because you used drugs?

(0) No

(1) Yes, but not in the last year

(2) Yes, during the last year

Shortname / Alias: drug_concern

ID: 169

Has a relative or friend, doctor or nurse, or anyone else been worried about your drug use or said that you should stop using drugs?

(0) No

(1) Yes, but not in the last year

(2) Yes, during the last year

Mental Health (MH1 - MH2)

Shortname / Alias: mh_

ID: 172

For the following, please choose the answer that best fits how you behaved during the past week.

	**Rarely or none of the time (<1 day) (0)**	**Some or little of the time (1-2 days) (1)**	**Occasionally or a moderate amount of time (3-4 days) (2)**	**Most or all of the time (5-7 days) (3)**
I was bothered by things that usually don't bother me [MH_BOTHER]	( )	( )	( )	( )
I had trouble keeping my mind	( )	( )	( )	( )

on what I was doing [MH_FOCUS]				
I felt depressed [MH_DEPRESS]	( )	( )	( )	( )
I felt everything was an effort [MH_EFFORT]	( )	( )	( )	( )
I felt hopeful about the future [MH_FUTURE]	( )	( )	( )	( )
I felt fearful [MH_FEAR]	( )	( )	( )	( )
My sleep was restless [MH_RESTLESS]	( )	( )	( )	( )
I was happy [MH_HAPPY]	( )	( )	( )	( )
I felt lonely [MH_LONELY]	( )	( )	( )	( )
I could not get "going" [MH_GOING]	( )	( )	( )	( )

Shortname / Alias: mh_

ID: 717

In your life, have you ever had any experience that was so frightening, horrible, or upsetting that, in the past month, you:

	**Yes**	**No**

	**(1)**	**(0)**
Have had nightmares about it or thought about it when you did not want to? [MH_NIGHTMARE]	( )	( )
Tried hard not think about it or went out of your way to avoid situations that reminded you of it? [MH_AVOID]	( )	( )
Were constantly on guard, watchful, or easily startled? [MH_GUARD]	( )	( )
Felt numb or detached from others, activities, or your surroundings? [MH_NUMB]	( )	( )

Tech Use (TU1 - TU5)

Page exit logic: Skip / Disqualify Logic**IF:** (((((((((((((Question "Do you do any of the following online or on your smartphone (*e.g.* Android or iPhone)? Select all that apply." is exactly equal to ("Use social media, like Facebook or Twitter") OR Question "Do you do any of the following online or on your smartphone (*e.g.* Android or iPhone)? Select all that apply." is exactly equal to ("Send Email")) OR Question "Do you do any of the following online or on your smartphone (*e.g.* Android or iPhone)? Select all that apply." is exactly equal to ("Send text messages")) OR Question "Do you do any of the following online or on your smartphone (*e.g.* Android or iPhone)? Select all that apply." is exactly equal to ("Use messaging apps, like Whatsapp or Kik")) OR Question "Do you do any of the following online or on your smartphone (*e.g.* Android or iPhone)? Select all that apply." is exactly equal to ("Use health and fitness apps,

like MyFitnessPal or Fitbit")) OR Question "Do you do any of the following online or on your smartphone (*e.g*. Android or iPhone)? Select all that apply." is exactly equal to ("Use dating and hookup apps, like Grindr or Jack'd")) OR Question "Do you do any of the following online or on your smartphone (*e.g*. Android or iPhone)? Select all that apply." is exactly equal to ("Read or comment on discussion boards, like Reddit or Digg")) OR Question "Do you do any of the following online or on your smartphone (*e.g*. Android or iPhone)? Select all that apply." is exactly equal to ("Read the news or political blogs")) OR Question "Do you do any of the following online or on your smartphone (*e.g*. Android or iPhone)? Select all that apply." is exactly equal to ("Video call or chat")) OR Question "Do you do any of the following online or on your smartphone (*e.g*. Android or iPhone)? Select all that apply." is exactly equal to ("Use music apps, like Spotify or Pandora")) OR Question "Do you do any of the following online or on your smartphone (*e.g*. Android or iPhone)? Select all that apply." is exactly equal to ("Play video games")) OR Question "Do you do any of the following online or on your smartphone (*e.g*. Android or iPhone)? Select all that apply." is exactly equal to ("Watch videos, TV shows or movies")) OR Question "Do you do any of the following online or on your smartphone (*e.g*. Android or iPhone)? Select all that apply." is exactly equal to ("Use apps that automatically delete the messages you send, like Snapchat")) OR Question "Do you do any of the following online or on your smartphone (*e.g*. Android or iPhone)? Select all that apply." is exactly equal to ("Visit anonymous sharing or questions apps, like Whisper, YikYak or Ask.fm")) **THEN:** Jump to page 111 - Tech Use (TU7 - TU8)

Logic: Show/hide trigger exists.

Shortname / Alias: internet

ID: 378

Do you access the internet on a cell phone, tablet, laptop or computer at least occasionally?

(1) Yes

(0) No

Logic: Hidden unless: Question "Do you access the internet on a cell phone, tablet, laptop or computer at least occasionally?" is one of the following answers ("Yes")

Shortname / Alias: net_freq

ID: 379

On average, how often have you used the Internet in the past 3 months, that is since [question('value'), id='760']?

(1) Less than once a week

(2) Once a week

(3) Several times per week

(4) Once or twice per day

(5) 1-2 hours per day

(6) 2-3 hours per day

(7) 3-4 hours per day

(8) 4 or more hours per day

Shortname / Alias: own_

ID: 380

Do you personally own or have access to the following items? Select all that apply.

[ ] A smartphone like an Android or iPhone [OWN_SMARTPHONE]

[ ] A cell phone that is not a smartphone [OWN_CELLPHONE]

[ ] A desktop or laptop computer [OWN_COMPUTER]

[ ] A tablet like an iPad, Samsung Galaxy or Kindle Fire [OWN_TABLET]

[ ] A gaming console like an Xbox, Playstation or Wii [OWN_GAMING]

[ ] None of the above [OWN_NONE]

Shortname / Alias: phonehrs

ID: 381

On average, how many hours per day do you spend on your cellphone?

(1) Less than 1 hour per day

(2) 1-2 hours per day

(3) 2-3 hours per day

(4) 3-4 hours per day

(5) More than 4 hours per day

Shortname / Alias: tech_

ID: 382

Do you do any of the following online or on your smartphone (*e.g.* Android or iPhone)? Select all that apply.

[ ] Use social media, like Facebook or Twitter [TECH_FB]

[ ] Send Email [TECH_EMAIL]

[ ] Send text messages [TECH_TEXT]

[ ] Use messaging apps, like Whatsapp or Kik [TECH_KIK]

[ ] Use health and fitness apps, like MyFitnessPal or Fitbit [TECH_FITNESS]

[ ] Use dating and hookup apps, like Grindr or Jack'd [TECH_DATE]

[ ] Read or comment on discussion boards, like Reddit or Digg [TECH_REDDIT]

[ ] Read the news or political blogs [TECH_NEWS]

[ ] Video call or chat [TECH_VIDEO]

[ ] Use music apps, like Spotify or Pandora [TECH_MUSIC]

[ ] Play video games [TECH_GAMES]

[ ] Watch videos, TV shows or movies [TECH_TV]

[ ] Use apps that automatically delete the messages you send, like Snapchat [TECH_SNAPCHAT]

[ ] Visit anonymous sharing or questions apps, like Whisper, YikYak or Ask.fm [TECH_YIKYAK]

[ ] None of the above [TECH_NONE]

Tech Use (TU6)

Shortname / Alias: tech_main

ID: 383

Piping: Piped Values From Question (Do you do any of the following online or on your smartphone (*e.g.* Android or iPhone)? Select all that apply.)

Of the activities you selected, which do you spend most of your time online or on your smartphone doing?

Tech Use (TU7 - TU8)

Page exit logic: Skip / Disqualify Logic**IF:** ((((((((((((((((((((((((((((((((((((Question "In the **past 3 months**, that is since [question('value'), id='760'], which of the following apps and websites have you used? Select all that apply." is exactly equal to ("Facebook") OR Question "In the **past 3 months**, that is since [question('value'), id='760'], which of the following apps and websites have you used? Select all that apply." is exactly equal to ("Twitter")) OR Question "In the **past 3 months**, that is since [question('value'), id='760'], which of the following apps and websites have you used? Select all that apply." is exactly equal to ("Instagram")) OR Question "In the **past 3 months**, that is since [question('value'), id='760'], which of the following apps and websites have you used? Select all that apply." is exactly equal to ("Snapchat")) OR Question "In the **past 3 months**, that is since [question('value'), id='760'], which of the following apps and websites have you used? Select all that apply." is exactly equal to ("Facebook messenger")) OR Question "In the **past 3 months**, that is since [question('value'), id='760'], which of the following apps and websites have you used? Select all that apply." is exactly equal to ("Gmail")) OR Question "In the **past 3 months**, that is since [question('value'), id='760'], which of the following apps and websites have you used? Select all that apply." is exactly equal to ("Youtube")) OR Question "In the **past 3 months**, that is since [question('value'), id='760'], which of the following apps and websites have you used? Select all that apply." is exactly equal to ("Pandora")) OR Question "In the **past 3 months**, that is since [question('value'), id='760'], which of the following apps and websites have you used? Select all that apply." is exactly equal to ("Netflix")) OR Question "In the **past 3 months**, that is since [question('value'), id='760'], which of the following apps and websites have you used? Select all that apply." is exactly equal to ("Spotify")) OR Question "In the **past 3 months**, that is since [question('value'), id='760'], which of the following apps and websites have you used? Select all that apply." is exactly equal to ("Whatsapp")) OR Question "In the **past 3 months**, that is since [question('value'), id='760'], which of the following apps and websites have you used? Select all that apply." is exactly equal to ("Kik")) OR Question "In the **past 3 months**, that is since [question('value'), id='760'], which of the following apps and websites have you used? Select all that apply." is exactly equal to ("Match")) OR Question "In the **past 3 months**, that is since [question('value'), id='760'], which of the following apps and websites have you used? Select all that apply." is exactly equal to ("Ok Cupid")) OR Question "In the **past 3 months**, that is since [question('value'), id='760'], which of the following apps and websites have you used? Select all that apply." is exactly equal to ("Tinder")) OR Question "In the **past 3 months**, that is since [question('value'), id='760'], which of the following apps and websites have you used? Select all that apply." is exactly equal to ("Craigslist")) OR Question "In the **past 3 months**, that is since [question('value'), id='760'], which of the following apps and websites have you used? Select all that apply." is exactly equal to ("Adam4Adam")) OR Question "In the **past 3 months**, that is since [question('value'), id='760'], which of the following apps and websites have you used? Select all that apply." is exactly equal to ("Bareback Real Time (BBRT)")) OR Question "In the **past 3 months**, that is since [question('value'), id='760'],

which of the following apps and websites have you used? Select all that apply." is exactly equal to ("Daddyhunt")) OR Question "In the **past 3 months**, that is since [question('value'), id='760'], which of the following apps and websites have you used? Select all that apply." is exactly equal to ("Dudesnude")) OR Question "In the **past 3 months**, that is since [question('value'), id='760'], which of the following apps and websites have you used? Select all that apply." is exactly equal to ("Gay.com")) OR Question "In the **past 3 months**, that is since [question('value'), id='760'], which of the following apps and websites have you used? Select all that apply." is exactly equal to ("Grindr")) OR Question "In the **past 3 months**, that is since [question('value'), id='760'], which of the following apps and websites have you used? Select all that apply." is exactly equal to ("Hornet")) OR Question "In the **past 3 months**, that is since [question('value'), id='760'], which of the following apps and websites have you used? Select all that apply." is exactly equal to ("Manhunt")) OR Question "In the **past 3 months**, that is since [question('value'), id='760'], which of the following apps and websites have you used? Select all that apply." is exactly equal to ("Jack'd")) OR Question "In the **past 3 months**, that is since [question('value'), id='760'], which of the following apps and websites have you used? Select all that apply." is exactly equal to ("Growlr")) OR Question "In the **past 3 months**, that is since [question('value'), id='760'], which of the following apps and websites have you used? Select all that apply." is exactly equal to ("Scruff")) OR Question "In the **past 3 months**, that is since [question('value'), id='760'], which of the following apps and websites have you used? Select all that apply." is exactly equal to ("SilverDaddies")) OR Question "In the **past 3 months**, that is since [question('value'), id='760'], which of the following apps and websites have you used? Select all that apply." is exactly equal to ("Squirt")) OR Question "In the **past 3 months**, that is since [question('value'), id='760'], which of the following apps and websites have you used? Select all that apply." is exactly equal to ("Vine")) OR Question "In the **past 3 months**, that is since [question('value'), id='760'], which of the following apps and websites have you used? Select all that apply." is exactly equal to ("Tumblr")) OR Question "In the **past 3 months**, that is since [question('value'), id='760'], which of the following apps and websites have you used? Select all that apply." is exactly equal to ("Whisper")) OR Question "In the **past 3 months**, that is since [question('value'), id='760'], which of the following apps and websites have you used? Select all that apply." is exactly equal to ("YikYak")) OR Question "In the **past 3 months**, that is since [question('value'), id='760'], which of the following apps and websites have you used? Select all that apply." is exactly equal to ("Wickr")) OR Question "In the **past 3 months**, that is since [question('value'), id='760'], which of the following apps and websites have you used? Select all that apply." is exactly equal to ("MyFitnessPal")) OR Question "In the **past 3 months**, that is since [question('value'), id='760'], which of the following apps and websites have you used? Select all that apply." is exactly equal to ("Fitbit")) OR Question "In the **past 3 months**, that is since [question('value'), id='760'], which of the following apps and websites have you used? Select all that apply." is exactly equal to [NO OPTIONS SET]) **THEN:** Jump to page 113 - Tech Use (TU10 - TU15)

Shortname / Alias: app_

ID: 384

In the past 3 months, that is since [question('value'), id='760'], which of the following apps and websites have you used? Select all that apply.

[ ] Facebook [APP_FACEBOOK]

[ ] Twitter [APP_TWITTER]

[ ] Instagram [APP_INSTAGRAM]

[ ] Snapchat [APP_SNAPCHAT]

[ ] Facebook messenger [APP_MESSENGER]

[ ] Gmail [APP_GMAIL]

[ ] Youtube [APP_YOUTUBE]

[ ] Pandora [APP_PANDORA]

[ ] Netflix [APP_NETFLIX]

[ ] Spotify [APP_SPOTIFY]

[ ] Whatsapp [APP_WHATSAPP]

[ ] Kik [APP_KIK]

[ ] Match [APP_MATCH]

[ ] Ok Cupid [APP_OKCUPID]

[ ] Tinder [APP_TINDER]

[ ] Craigslist [APP_CRAIGSLIST]

[ ] Adam4Adam [APP_ADAM]

[ ] Bareback Real Time (BBRT) [APP_BBRT]

[ ] Daddyhunt [APP_DADDYHUNT]

[ ] Dudesnude [APP_DUDESNUDE]

[ ] Gay.com [APP_GAYCOM]

[ ] Grindr [APP_GRINDR]

[ ] Hornet [APP_HORNET]

[ ] Manhunt [APP_MANHUNT]

[ ] Jack'd [APP_JACKD]

[ ] Growlr [APP_GROWLR]

[ ] Scruff [APP_SCRUFF]

[ ] SilverDaddies [APP_SILVER]

[ ] Squirt [APP_SQUIRT]

[ ] Vine [APP_VINE]

[ ] Tumblr [APP_TUMBLR]

[ ] Whisper [APP_WHISPER]

[ ] YikYak [APP_YIKYAK]

[ ] Wickr [APP_WICKR]

[ ] MyFitnessPal [APP_FITNESS]

[ ] Fitbit [APP_FITBIT]

[ ] Other, please specify:: ____
[APP_OTHER] [APP_OTHERSP]

Page entry logic: This page will show when: Question "In the **past 3 months**, that is since [question('value'), id='760'], which of the following apps and websites have you used? Select all that apply." is one of the following answers ("Facebook","Twitter","Instagram","Snapchat","Facebook messenger","Gmail","Youtube","Pandora","Netflix","Spotify","Whatsapp","Kik","Match","Ok Cupid","Tinder","Craigslist","Adam4Adam","Bareback Real Time (BBRT)","Daddyhunt","Dudesnude","Gay.com","Grindr","Hornet","Manhunt","Jack'd","Growlr","Scruff","SilverDaddies","Squirt","Vine","Tumblr","Whisper","YikYak","Wickr","MyFitnessPal","Fitbit")

Tech Use (TU9)

Validation: Max. answers = 3 *(if answered)*

Shortname / Alias: app_main

ID: 445

Piping: Piped Values From Question (In the **past 3 months**, that is since [question('value'), id='760'], which of the following apps and websites have you used? Select all that apply.)

Of the apps and websites you selected, which have you used most frequently in the past 3 months, that is since [question('value'), id='760']? Please select up to 3.

Tech Use (TU10 - TU15)

Logic: Show/hide trigger exists.

Shortname / Alias: app_

ID: 385

In the past 3 months, that is since [question('value'), id='760'], did you do any of the following online or through an app on your smartphone? Select all that apply.

[ ] Chat with men [APP_CHAT]

[ ] Make friends [APP_FRIENDS]

[ ] Found long-term partnerships [APP_PARTNER]

[ ] Met men for casual sex [APP_SEX]

[ ] Had online sex (cyber sex) [APP_ONLINESEX]

[ ] None of the above [APP_NONEa]

Logic: Hidden unless: Question "In the past 3 months, that is since [question('value'), id='760'], did you do any of the following online or through an app on your smartphone? Select all that apply." is one of the following answers ("Chat with men","Make friends","Found long-term partnerships","Met men for casual sex","Had online sex (cyber sex)")

Shortname / Alias: tech_meetmen

ID: 386

In the past 3 months, that is since [question('value'), id='760'], how often have you used the internet or a smartphone app to meet other gay men?

(1) Once a month or less

(2) 2-3 times per month

(3) About once a week

(4) 2-6 times per week

(5) More than once a day

Logic: Hidden unless: (Question "In the past 3 months, that is since [question('value'), id='760'], did you do any of the following online or through an app on your smartphone? Select all that apply." is one of the following answers ("Chat with men","Make

friends","Found long-term partnerships","Met men for casual sex","Had online sex (cyber sex)") AND Question "In the past 3 months, that is since [question('value'), id='760'], which of the following apps and websites have you used? Select all that apply." is one of the following answers ("Match","Ok Cupid","Tinder","Craigslist","Adam4Adam","Bareback Real Time (BBRT)","Daddyhunt","Dudesnude","Gay.com","Grindr","Hornet","Manhunt","Jack'd","Growlr","Scruff","SilverDaddies","Squirt"))

Shortname / Alias: meetmen_app

ID: 387

Earlier, you mentioned that you used one of the following dating or hookup sites.

In the past 3 months, that is since [question('value'), id='760'], which of the following have you used most frequently for meeting other men?

(1) Match

(2) Ok Cupid

(3) Tinder

(4) Craigslist

(5) Adam4Adam

(6) Bareback Real Time (BBRT)

(7) Daddyhunt

(8) Dudesnude

(9) Gay.com

(10) Grindr

(11) Hornet

(12) Manhunt

(13) Jack'd

(14) Growlr

(15) Scruff

(16) SilverDaddies

(17) Squirt

(18) Other, please specify:: ____

Validation: Must be numeric Whole numbers only Positive numbers only

Shortname / Alias: internetsex

ID: 539

In the past 3 months, that is since [question('value'), id='760'], how many sex partners have you met through the internet?

____

Exchange Sex (EX1 - EX4)

Logic: Show/hide trigger exists.

Shortname / Alias: exch_give

ID: 186

Have you ever given things, like money, drugs, or a place to stay, in exchange for sex?

(1) Yes

(0) No

Logic: Hidden unless: Question "Have you ever given things, like money, drugs, or a place to stay, in exchange for sex?" is one of the following answers ("Yes")

Shortname / Alias: exch_give3m

ID: 188

In the past 3 months, that is since [question('value'), id='760'], have you given things, like money, drugs, or a place to stay, in exchange for sex?

(1) Yes

(0) No

Logic: Show/hide trigger exists.

Shortname / Alias: exch_receive

ID: 187

Has anyone ever given you things, like money, drugs, or a place to stay, in exchange for sex?

(1) Yes

(0) No

Logic: Hidden unless: Question "Has anyone ever given you things, like money, drugs, or a place to stay, in exchange for sex?" is one of the following answers ("Yes")

Shortname / Alias: exch_receive3m

ID: 189

In the past 3 months, that is since [question('value'), id='760'], has anyone given you things, like money, drugs, or a place to stay, in exchange for sex?

(1) Yes

(0) No

IPV (IPV1 - IPV5)

Shortname / Alias: ipv_sex

ID: 190

In the past 3 months, that is since [question('value'), id='760'], has a partner pressured or forced you to do something sexual that you didn't want to do? Examples may include any of the following: oral or anal sex, having sex with others, having sexual partners outside the relationship, or any other sexual activity that made you feel uncomfortable.

(1) Yes

(0) No

Shortname / Alias: ipv_hit

ID: 191

In the past 3 months, that is since [question('value'), id='760'], have arguments in your relationship escalated into any of the following: destruction of property, grabbing, restraining, pushing, kicking, slapping, punching, threats of violence or other acts of physical intimidation?

(1) Yes

(0) No

Shortname / Alias: ipv_words

ID: 192

In the past 3 months, that is since [question('value'), id='760'], has a partner insulted, criticized, threatened or yelled at you in any way? Examples may include the following: using slurs, calling you names, calling you fat, criticizing your sexual performance, criticizing your clothing, asking you to act more masculine or threatening to out you.

(1) Yes

(0) No

Shortname / Alias: ipv_prevent

ID: 193

In the past 3 months, that is since [question('value'), id='760'], has a partner prevented you from communicating with or seeing your friends, family, or coworkers?

(1) Yes

(0) No

Shortname / Alias: ipv_monitor

ID: 830

In the past 3 months, that is since [question('value'), id='760'], has a partner monitored or demanded access to your cell phone, email, social networking sites, finances or spending?

(1) Yes

(0) No

Shortname / Alias: ipv_afraid

ID: 194

In the past 3 months, that is since [question('value'), id='760'], have you ever felt afraid, threatened, isolated, trapped as a result of a relationship?

(1) Yes

(0) No

Shortname / Alias: ipv_famconcern

ID: 851

In the past 3 months, that is since [question('value'), id='760'], have your friends or family raised concerns about your safety within your relationship?

(1) Yes

(0) No

Resiliency (RS1)

Page exit logic: Skip / Disqualify Logic**IF:** Question "What is your HIV status?" is one of the following answers ("HIV Negative","Indeterminate","I don't know") **THEN:** Jump to page 118 - HIV/AIDS Conspiracy Scale (CONS1)

Shortname / Alias: resilient

ID: 389

How well do the following statements describe how you felt in the past month?

	Not at all true (0)	Rarely true (1)	Sometimes true (2)	Often true (3)	True nearly all of the time (4)
I am able to adapt to change [RESILIENT_1]	( )	( )	( )	( )	( )
I tend to bounce back after illness or hardship [RESILIENT_2]	( )	( )	( )	( )	( )

Page entry logic: This page will show when: Question "What is your HIV status?" is one of the following answers ("HIV Positive")

HIV-related Stigma (STG1)

Shortname / Alias: stigma_

ID: 195

Please indicate how much you agree with the following statements:

	Strongly Disagree (1)	Disagree (2)	Neutral (3)	Agree (4)	Strongly Agree (5)
I have lost friends by	( )	( )	( )	( )	( )

telling them I am HIV-positive [STIGMA_1]					
I am hurt by how people reacted to learning I am HIV-positive [STIGMA_2]	( )	( )	( )	( )	( )
People avoid touching me if they know I am HIV-positive [STIGMA_3]	( )	( )	( )	( )	( )
I have stopped socializing with some people due to their reactions to me being HIV-positive [STIGMA_4]	( )	( )	( )	( )	( )
People I care about stopped calling me after learning that I am HIV-positive [STIGMA_5]	( )	( )	( )	( )	( )
People have physically backed away from me because I am	( )	( )	( )	( )	( )

HIV-positive [STIGMA_6]					
Some people who know I am HIV-positive have grown more distant [STIGMA_7]	( )	( )	( )	( )	( )
People who know that I am HIV-positive ignore my good points [STIGMA_8]	( )	( )	( )	( )	( )
People don't want me around their children once they know that I am HIV-positive [STIGMA_9]	( )	( )	( )	( )	( )

HIV/AIDS Conspiracy Scale (CONS1)

Page exit logic: Skip / Disqualify Logic**IF:** Question "What is your HIV status?" is one of the following answers ("HIV Positive") **THEN:** Jump to page 120 - Perceived Severity of HIV (SEV1)

Shortname / Alias: consp_

ID: 718

Please answer the next questions by indicating how much you agree with each statement:

	**Strongly Disagree (1)**	**Disagree (2)**	**Neither (3)**	**Agree (4)**	**Strongly Agree (5)**
HIV is a manmade virus. [CONSP_1]	( )	( )	( )	( )	( )
AIDS was produced in a government laboratory. [CONSP_2]	( )	( )	( )	( )	( )
There is a cure for AIDS, but it is being withheld from the poor. [CONSP_3]	( )	( )	( )	( )	( )
AIDS was created by the government to control some populations. [CONSP_4]	( )	( )	( )	( )	( )
AIDS is a form of genocide, or planned destruction,	( )	( )	( )	( )	( )

against some groups. [CONSP_5]					
HIV was created and spread by the CIA. [CONSP_6]	( )	( )	( )	( )	( )
People who take the new medications for HIV are human guinea pigs for the government. [CONSP_7]	( )	( )	( )	( )	( )
The medication used to treat HIV causes people to get AIDS. [CONSP_8]	( )	( )	( )	( )	( )

Page entry logic: This page will show when: Question "What is your HIV status?" is one of the following answers ("HIV Negative","Indeterminate","I don't know")

Perceived Severity of HIV (SEV1)

Page exit logic: Skip / Disqualify Logic**IF:** Question "What is your HIV status?" is one of the following answers ("HIV Negative","Indeterminate","I don't know") **THEN:** Jump to page 121 - Healthcare Trust/Mistrust (HCTR1)

Shortname / Alias: severity_neg

ID: 727

Please indicate how much you agree with the following statement:

	Strongly Disagree (1)	Disagree (2)	Neither (3)	Somewhat Agree (4)	Strongly Agree (5)
"Because of medications for HIV, I am less concerned about becoming HIV-positive."	( )	( )	( )	( )	( )

Page entry logic: This page will show when: Question "What is your HIV status?" is one of the following answers ("HIV Positive")

Perceived Severity of HIV (SEV1)

Shortname / Alias: severity_pos

ID: 732

Please indicate how much you agree with the following statement:

	Strongly Disagree (1)	Disagree (2)	Neither (3)	Somewhat Agree (4)	Strongly Agree (5)

"Because of medications for HIV, I am less concerned about infecting someone"	( )	( )	( )	( )	( )

Healthcare Trust/Mistrust (HCTR1)

Shortname / Alias: healthcare_

ID: 219

Please indicate how much you agree with the following statements:

	**Strongly Disagree (1)**	**Disagree (2)**	**Neutral (3)**	**Agree (4)**	**Strongly Agree (5)**
Doctors and healthcare workers sometimes hide information from patients who belong to my race or ethnic group [HEALTHCARE_1]	( )	( )	( )	( )	( )
Doctors have the best interests of people of my race or ethnic group in mind [HEALTHCARE_2]	( )	( )	( )	( )	( )

People of my race or ethnic group should not confide in doctors and healthcare workers because it will be used against them [HEALTHCARE_3]	( )	( )	( )	( )	( )
People of my race or ethnic group should be suspicious of information from doctors and healthcare workers [HEALTHCARE_4]	( )	( )	( )	( )	( )
People of my race or ethnic group cannot trust doctors and healthcare workers [HEALTHCARE_5]	( )	( )	( )	( )	( )
People of my race or ethnic group should be suspicious of modern medicine [HEALTHCARE_6]	( )	( )	( )	( )	( )
Doctors and healthcare workers treat people of my race or ethnic group as "guinea pigs" [HEALTHCARE_7]	( )	( )	( )	( )	( )
People of my race or ethnic group receive the same medical care from doctors and healthcare workers as people from other groups [HEALTHCARE_8]	( )	( )	( )	( )	( )

Doctors and healthcare workers do not take the medical complaints of people of my race or ethnic group seriously [HEALTHCARE_9]	( )	( )	( )	( )	( )
People of my race or ethnic group are treated the same as people of other groups by doctors and healthcare workers [HEALTHCARE_10]	( )	( )	( )	( )	( )
In most hospitals, people of different race or ethnic groups receive the same kind of care [HEALTHCARE_11]	( )	( )	( )	( )	( )
I have personally been treated poorly or unfairly by doctors or healthcare workers because of my race or ethnicity [HEALTHCARE_12]	( )	( )	( )	( )	( )

Percent Completed Script

Action: Custom Script: Percent of Survey Completed

Hidden Value: percentcomplete

Value:

Hidden Value: Time Spent on Survey

Value: Populates with the **length of time** since the survey taker started the survey

---

Email Actions

Email action: Baseline Completion - Atlanta

To: mobilemessaging@emory.edu
From: SurveyGizmo (noreply@surveygizmo.com)
Subject: M3 Baseline Completion - PID [question("value"), id="842"]

Email action: Baseline Completion - Detroit

To: M3Project@umich.edu
From: SurveyGizmo (noreply@surveygizmo.com)
Subject: M3 Baseline Completion - PID [question("value"), id="842"]

Email action: Baseline Completion - New York

To: mobilemessaging@healthsolutions.org
From: SurveyGizmo (noreply@surveygizmo.com)
Subject: M3 Baseline Completion - PID [question("value"), id="842"]

Thank You!

ID: 1

Thank you for taking our survey!
Your responses have been very helpful.

Please tell study staff that you have completed the survey. Do not close out of this window.

ID: 850

[question("value"), id="829"]
